# Supplementary material for: Mental health and well-being of older adults living with HIV in sub-Saharan Africa: a systematic review
Source: BMJ Open. 2021 Sep 22;11(9):e052810. doi: 10.1136/bmjopen-2021-052810 (PMC8461287; doi:10.1136/bmjopen-2021-052810)
Supplement: Supplementary data [file bmjopen-2021-052810supp001.pdf]

Supplementary Files – Mental health and Wellbeing of older adults living with HIV in sub-Saharan Africa: a systematic review

Supplementary file 1. Search strategy

We adopted a stepwise approach that involves first combining shorter keywords for each part of the PICOS criteria with "OR" and later checking history for the list of searches and combining them with "AND." No filters were applied.

The following search terms were included:

- HIV OR HIV-1 OR HIV/AIDS OR HIV infections
- adult OR older adult OR older people OR older individual OR elderly
- Africa OR sub-Saharan Africa OR Africa South of the Sahara
- cognitive impairment OR neurocognitive impairment OR neurological complication OR HIV-associated neurocognitive disorder
- common mental disorder OR depression OR depressive symptoms OR depressive disorder OR anxiety OR anxiety disorder
- quality of life OR health-related quality of life
- grip strength OR hand strength OR frailty
-

<u>PubMed search</u> – 13/01/2021:

((((((HIV) OR (HIV-1)) OR (HIV/AIDS)) OR (HIV infections)) AND (((((Adult) OR (older adult)) OR (older people)) OR (older individual)) OR (elderly))) AND (((Africa) OR (sub-Saharan Africa)) OR (Africa South of the Sahara))) AND ((((((((((((((cognitive impairment) OR (neurocognitive impairment)) OR (neurological complication)) OR (HIV-associated neurocognitive disorder)) OR (common mental disorder)) OR (depression)) OR (depressive symptoms)) OR (depressive disorder)) OR (anxiety)) OR (anxiety disorder)) OR (quality of life)) OR (health-related quality of life)) OR (grip strength)) OR (hand strength)) OR (frailty))

Number of hits = 2,090

<u>PsycINFO search – 13/01/2021</u>:

((HIV or HIV-1 or HIV AIDS or HIV infections) and (adult or older adult or older people or older individual or elderly) and (Africa or sub-Saharan Africa or Africa South of the Sahara) and (cognitive impairment or neurocognitive impairment or neurological complication or HIV-associated neurocognitive disorder or common mental disorder or depression or depressive symptoms or depressive disorder or anxiety or anxiety disorder or quality of life or health-related quality of life or grip strength or hand strength or frailty)).af.

Number of hits = 3,691

Mwangala PN, *et al. BMJ Open* 2022; 11:e052810. doi: 10.1136/bmjopen-2021-052810

Supplementary Files – Mental health and Wellbeing of older adults living with HIV in sub-Saharan Africa: a systematic review

Embase search – 13/01/2021:

((HIV or HIV-1 or HIV AIDS or HIV infections) and (adult or older adult or older people or older individual or elderly) and (Africa or sub-Saharan Africa or Africa South of the Sahara) and (cognitive impairment or neurocognitive impairment or neurological complication or HIV-associated neurocognitive disorder or common mental disorder or depression or depressive symptoms or depressive disorder or anxiety or anxiety disorder or quality of life or health-related quality of life or grip strength or hand strength or frailty)).af.

Number of hits = 1284

Scopus search – 13/01/2021:

TITLE-ABS-KEY (((((((hiv) OR (hiv-1)) OR (hiv/aids)) OR (hiv AND infections)) AND (((((adult) OR (older AND adult)) OR (older AND people)) OR (older AND individual)) OR (elderly))) AND (((africa) OR (sub-saharan AND africa)) OR (africa AND south AND of AND the AND sahara))) AND ((((((((((((((cognitive AND impairment) OR (neurocognitive AND impairment)) OR (neurological AND complication)) OR (hiv-associated AND neurocognitive AND disorder)) OR (common AND mental AND disorder)) OR (depression)) OR (depressive AND symptoms)) OR (depressive AND disorder)) OR (anxiety)) OR (anxiety AND disorder)) OR (quality AND of AND life)) OR (health-related AND quality AND of AND life)) OR (grip AND strength)) OR (hand AND strength)) OR (frailty)))

Number of hits = 1295

CINAHL search – 13/01/2021:

((HIV OR HIV-1 OR HIV AIDS OR HIV infections)) AND ((adult OR older adult OR older people OR older individual OR elderly)) AND ((Africa OR sub-Saharan Africa OR Africa South of the Sahara)) AND ((cognitive impairment OR neurocognitive impairment OR neurological complication OR HIV-associated neurocognitive disorder OR common mental disorder OR depression OR depressive symptoms OR depressive disorder OR anxiety OR anxiety disorder OR quality of life OR health-related quality of life OR grip strength OR hand strength OR frailty))

Number of hits = 381

Total number of hits across the databases = 8,741 articles

Mwangala PN, *et al. BMJ Open* 2022; 11:e052810. doi: 10.1136/bmjopen-2021-052810

Supplementary Files – Mental health and Wellbeing of older adults living with HIV in sub-Saharan Africa: a systematic review

Supplementary Table 1: Summary of prevalence estimates and correlates for common mental disorders

Author, publication year & country	Study design	Sample size	Treatment status of PLWH	Cut-off score of the tool used	Information on local tool validation	Prevalence estimates/other results for PLWH50+	Correlates reported
Studies that used the 9-item Patient Health Questionnaire (PHQ-9) to assess depression							
Abadiga, M. (2019); Ethiopia	Cross-sectional	55 PLWH48+; 338 PLWH (18-48 years)	All on ART	≥5	Previously validated in Ethiopia	49.1%	Reported correlates not aggregated by age.
Eshetu, D. A., et al. (2015) ; Ethiopia	Cross-sectional	60 PLWH50+; 356 PLWH (20-49 years)	56.2% on ART	≥5	Sensitivity of 88% and Specificity of 88%	46.7%	Reported correlates not aggregated by age
Torgersen, J., et al. (2019); Botswana	Cross-sectional	125 PLWH50+; 789 PLWH (21-49 years)	ART naïve	≥10	NR	16.0%	NR
Cholera, R., et al. (2017); South Africa	Cross-sectional	28 PLWH50+; 312 PLWH (18-49 years)	ART naïve	≥10	Previously validated against the MINI yielding 78.7% sensitivity and 83.4% specificity in the study population	46.4%	NR
Asangbeh, S. L., et al. (2016) ; Cameroon	Cross-sectional	62 PLWH50+; 140 PLWH (21-49 years)	All on ART	≥10	Validated in a previous study in Cameroon	37.0%	Reported correlates not aggregated by age.
Duko, B., et al. (2018); Ethiopia	Cross-sectional	31 PLWH50+; 352 PLWH (18-54 years)	93.2% on ART	≥11	Previously validated yielding 86% sensitivity and 67% specificity	45.2%	Reported correlates not aggregated by age.
Studies that used the Center for Epidemiological Studies Depression scale (CES-D) to assess depression							
Bernard, C., et al. (2020); Coˆte d'Ivoire & Senegal	Cross-sectional	334 PLWH50+	All on ART	A total score ≥ 17 for men and ≥ 23 for women	Relied on previous validation of the tool from other African countries (Uganda, South Africa and Zambia)	17.9% (95% CI: 13.8-22).	PLWH with severe depressive symptoms were more likely to be **unemployed** (AOR = 2.8; 95% CI: 1.4–5.7); **current or former tobacco smokers** (AOR = 2.6; 95% CI: 1.3–5.4) but **were less likely to be overweight or obese** (AOR = 0.4; 95% CI: 0.2–0.8).
Rohr, J. K., et al. (2020); South Africa	Population survey	1048 PLWH40+; 3512 HIV uninfected older adults ≥40 years	68.6% on ART	≥4 symptoms	NR	8.0% among PLWH40+ versus 10.0% among HIV uninfected older adults ≥40 years.	NR
Moucheraud, C., et al. (2020); Malawi	Cross-sectional	74 PLWH50+ and 60 young PLWH [30-49] years.	All on ART	Mild/major depression symptoms = ≥16 CESD scores; Major depression symptoms = ≥27	NR	Symptoms of mild/major depression: 32.4% among PLWH50+; Major depression symptoms: 10.8% among PLWH50+	Older adults reported less few depressive symptoms (mild depression: AOR 0.23 p = 0.002; major depression: AOR 0.16, p = 0.004)

Mwangala PN, *et al. BMJ Open* 2022; 11:e052810. doi: 10.1136/bmjopen-2021-052810

Supplementary Files – Mental health and Wellbeing of older adults living with HIV in sub-Saharan Africa: a systematic review

Author, publication year & country	Study design	Sample size	Treatment status of PLWH	Cut-off score of the tool used	Information on local tool validation	Prevalence estimates/other results for PLWH50+	Correlates reported
Geldsetzer, P., et al. (2019); South Africa	Population-based survey	1,037 PLWH40+; 4,022 HIV uninfected older adults ≥40 years	63.9% on ART	NR	Not validated previously. The current version underwent intensive cultural adaptation	None of the chronic conditions was significantly associated with depressive symptoms in multivariable regressions.	Older age was positively associated with depression, whereas being married and employment was negatively associated.
Olley, B. O., et al. (2017) ; Nigeria	Cross-sectional	44 PLWH50+; 458 PLWH (18-50 years)	All on ART	≥15	Cronbach's alpha = 0.79. Item-total correlation coefficient ranged between 0.65 and 0.87	20.5%	Not aggregated by age
Kaharuza, F. M., et al. (2006) ; Uganda	Cross-sectional	94 PLWH50+; 923 PLWH (18-50 years)	ART naïve	≥23	Internally consistent (alpha = 0.9)	58.5%	Older age (>50 years) was positively associated with depressive symptoms; AOR 1.93 (95% CI: 1.09, 3.42).
Studies that used the Mini International Neuropsychiatric Interview (MINI) to assess depression							
Asrat, B., et al. (2020); Ethiopia	Cross-sectional	54 PLWH50+; 337 PLWH (18-49 years)	All on ART	NR	Previously adapted in Ethiopia	29.6%	Reported correlates not aggregated by age.
Musinguzi, K., et al. (2018); Uganda	Cross-sectional	21 PLWH50+; 180 PLWH (18-49 years)	ART naïve	NR	NR	14.3%	Reported correlates not aggregated by age.
Kinyanda, E., et al. (2016); Uganda	Cross-sectional	244 PLWH50+; 224 HIV affected but uninfected older adults ≥50 years	9.8% on ART	NR	NR	11.9% among PLWH50+; 6.3% in HIV affected but uninfected older adults ≥50 years	MDD was significantly associated with declining SES, increasing disability scores, decreasing mean grip strength, reported back pain, and not having hypertension.
Mugisha, J. O., et al. (2016) ; Uganda	Cross-sectional	244 PLWH50+; 227 HIV uninfected older adults ≥50 years	90.6% on ART	NR	Locally adapted	9.5% among PLWH50+; 5.8% in the uninfected older adults ≥50 years	NR
Akena, D., et al. (2012); Uganda	Cross-sectional	51 PLWH50+; 317 PLWH (18-49 years)	NR	≥5 of the 9 DSM-IV-TR symptoms for major depression	Not locally validated	11.8%	Reported correlates not aggregated by age.

Mwangala PN, et al. BMJ Open 2022; 11:e052810. doi: 10.1136/bmjopen-2021-052810

Supplementary Files – Mental health and Wellbeing of older adults living with HIV in sub-Saharan Africa: a systematic review

Author, publication year & country	Study design	Sample size	Treatment status of PLWH	Cut-off score of the tool used	Information on local tool validation	Prevalence estimates/other results for PLWH50+	Correlates reported
Studies that used the geriatric depression scale to assess depression							
Kalomo, E. N., et al. (2020); Namibia	Cross-sectional	147 PLWH50+	All on ART	≥3 symptoms	Cronbach alpha of 0.73 in the current study	46.1%	There was a positive association between HIV stigma (negative self-perceptions) and depression and a negative relationship between resilience and depression. Also, being female and higher education were protective factors and disclosure of HIV status was a risk factor
Eaton, P., et al. (2020); Tanzania	Cross-sectional	253 PLWH50+	94.8% on ART	≥5 symptoms	NR	17.4%	Not reported
Studies that used the Composite International Diagnostic Interview (CIDI) to assess depression							
Nyirenda, M., et al. (2013); South Africa	Cross-sectional	203 PLWH50+; 219 HIV uninfected older adults ≥50 years	23.7% on ART	ICD 10-DCR criteria	Locally adapted	14.8% among PLWH50+; 30.1 % in the uninfected older adults ≥50 years	Being female (aOR 3.04, 95% CI 1.73–5.36), receiving a government grant (aOR 0.34, 95% CI 0.15–0.75), urban residency (aOR 1.86, 95% CI 1.16–2.96) and adult caregiving (aOR 2.37, 95% CI 1.37–4.12) were significantly associated with any depressive episode
Negin, J., et al. (2012); South Africa	Cross-sectional	142 PLWH50+; 2,722 HIV uninfected older adults ≥50 years	NR	NR	Locally adapted	6.0% among PLWH50+; 4.8% in the uninfected older adults ≥50 years	NR
Studies that used other methods to assess depression							
Kellett-Wright, J., et al. (2020); Tanzania	Cross-sectional	235 PLWH50+	95.5% on ART	DSM-IV; cut-off NR	NR	16.6%	NR
Motumma, A., et al. (2019); Ethiopia	Cross-sectional	122 PLWH40+; 298 HIV infected young adults (18-40) years	All on ART	A score of ≥7 of Self-Reporting Questionnaire (SRQ-20)	Previously validated in Ethiopia, with high sensitivity (85.7%) and specificity (75.6%).	23.0%	Correlates not aggregated by age
Manne-Goehler, J., et al. (2019); Uganda	Cross-sectional	154 PLWH40+; 142 HIV uninfected older adults ≥40 years	All on ART	Mean score of ≥1.75 on the 15-Item Hopkins Checklist (HSCL)	Previously validated in the same population	21.4% among PLWH40+; 33.8% in uninfected older adults ≥40 years	PLWH on ART and those falling in the highest wealth quartile had a lower prevalence of probable depression. In comparison, women had a significantly higher prevalence of depression than men. Education and age were not significantly associated with depression.

Mwangala PN, *et al. BMJ Open* 2022; 11:e052810. doi: 10.1136/bmjopen-2021-052810

Supplementary Files – Mental health and Wellbeing of older adults living with HIV in sub-Saharan Africa: a systematic review

Author, publication year & country	Study design	Sample size	Treatment status of PLWH	Cut-off score of the tool used	Information on local tool validation	Prevalence estimates/other results for PLWH50+	Correlates reported
Tesfaw, G., et al. (2016); Ethiopia	Cross-sectional	59 PLWH50+; 358 PLWH (19-49 years)	All on ART	A cut-off ≥8 on the 7-item Hospital Anxiety and Depression Scale	Previously validated	39.0%	Not aggregated by age
Shumba, C., et al. (2013); Uganda	Cross-sectional	91 PLWH50+; 584 PLWH (19-49 years)	All on ART	A cut-off of ≥1 on a locally adapted 5-item symptom questionnaire	Cronbach's alpha of 0.87	53.9%	NR
Berhe, H. and A. Bayray (2013); Ethiopia	Cross-sectional	19 PLWH54+; 250 PLWH (18-54 years)	NR	A score of ≥8 on the 21-Item Hamilton Depression Scale	NR	57.9%	Reported correlates not aggregated by age.
Studies reporting anxiety							
Obimakinde, A. M., et al. (2020) ; Nigeria	Cross-sectional	62 PLWH60+; 162 HIV uninfected older adults ≥60 years	All on ART	Extracted from participants clinical notes	NR	3.2% among PLWH50+; 3.2% in HIV uninfected older adults	NR
Olagunju, A. T., et al. (2012) ; Nigeria	Cross-sectional	24 PLWH50+; 276 PLWH (18-50 years)	All on ART	Clinical Assessment in Neuropsychiatry (SCAN);	NR	20.8%	Not aggregated by age

Notes: ART – *Antiretroviral treatment*; **AOR –** *Adjusted odds ratio*; **CES-D –** *Center for Epidemiologic Studies Depression Scale*; **CI –** *Confidence interval*; **CIDI –** *Composite International Diagnostic Interview***; DSM-IV–** *Diagnostic and Statistical Manual of Mental Disorders, fourth edition;* **HSCL –** *Hopkins Symptoms Checklist*; **MDD –** *Major depressive* disorder; **MINI –** *Mini International Neuropsychiatric Interview*; **NR –** *Not reported*; **PLWH –** *People living with HIV*; **PLWH50+ –** *People living with HIV ≥50 years old*; **SCAN –** *Clinical Assessment in Neuropsychiatry*

Mwangala PN, *et al. BMJ Open* 2022; 11:e052810. doi: 10.1136/bmjopen-2021-052810

Supplementary Files – Mental health and Wellbeing of older adults living with HIV in sub-Saharan Africa: a systematic review

Supplementary Table 2. Modified Newcastle-Ottawa Risk of Bias Assessment

Author	Representativeness of the sample	Sample size	Comparability between respondents and non-respondents	Outcome ascertainment	Quality of Statistics reporting	Total quality score
**Abadiga, M. (2019).**	**1**	**0**	**1**	**1**	**1**	**4**
Akena, D., et al. (2012).	1	0	0	0	1	2
Asangbeh, S. L., et al. (2016)	0	0	0	1	1	2
Asiimwe, S. B., et al. (2020).	1	1	1	1	1	5
Asrat, B., et al. (2020).	1	0	1	1	1	4
Atashili, J., et al. (2013)	0	0	1	0	1	2
Berhe, H. and A. Bayray (2013)	0	0	0	0	1	1
Bernard, C., et al. (2020).	1	1	0	0	1	3
Bernard, C., et al. (2020).	1	1	0	0	1	3
Bristow, C., et al. (2021).	1	1	1	1	1	5
Cassimjee, N. and P. K. Motswai (2017)	0	0	0	1	1	2
Cholera, R., et al. (2017).	1	0	1	1	1	4
Duko, B., et al. (2018).	1	0	0	1	1	3
Eaton, P., et al. (2020)	1	1	0	1	1	4
Edwards, A., et al. (2020).	1	1	1	0	1	4
Eshetu, D. A., et al. (2015).	0	0	0	0	1	1
Filteau, S., et al. (2017).	1	1	0	1	1	4
Geldsetzer, P., et al. (2019).	1	1	1	0	1	4
Harding, R., et al. (2014).	0	0	0	1	1	2
Joska, J. A., et al. (2019).	1	1	0	0	1	3
Kaharuza, F. M., et al. (2006)	1	0	0	1	1	3
Kalomo, E. N., et al. (2020)	0	1	0	0	1	2
Kellett-Wright, J., et al. (2020)	1	1	0	1	1	4
Kinyanda, E., et al. (2016)	1	1	0	1	1	4
Kobayashi, L. C., et al. (2019)	1	1	1	1	1	5
Maniragaba, F., et al. (2018)	1	0	0	0	1	2
Manne-Goehler, J., et al. (2019).	0	1	0	1	1	3
Motumma, A., et al. (2019).	1	1	1	1	1	5
Moucheraud, C., et al. (2020)	0	0	0	1	1	2
Mugendi, A., et al. (2019).	0	0	0	0	1	1
Mugisha, J., et al. (2013)	1	1	0	1	1	4
Mugisha, J. O., et al. (2016).	1	1	0	1	1	4
Musinguzi, K., et al. (2018).	1	0	0	0	1	2
Negin, J., et al. (2012)	1	1	0	1	1	4
Nyirenda, M., et al. (2012).	1	1	0	1	1	4
Nyirenda, M., et al. (2013).	1	1	0	1	1	4
Nyirenda, M., et al. (2013)	1	1	0	1	1	4
Obimakinde, A. M., et al. (2020).	0	0	1	0	1	2
Olagunju, A. T., et al. (2012).	1	0	0	1	1	3
Olley, B. O., et al. (2017).	0	0	0	1	1	2
Oumar, G., et al. (2020).	0	1	0	0	1	2
Parcesepe, A. M., et al. (2020).	0	0	0	1	1	2
Rohr, J. K., et al. (2020)	1	1	1	0	1	4
Scholten, F., et al. (2011).	1	1	0	1	1	4
Shumba, C., et al. (2013).	1	0	0	1	1	3
Ssonko, M., et al. (2018).	1	1	0	0	1	3
Tesfaw, G., et al. (2016)	1	0	1	1	1	4
Torgersen, J., et al. (2019).	0	1	1	0	1	3
Tsegaw, M., et al. (2017)	0	0	1	0	1	2
Yaya, I., et al. (2019).	1	1	1	0	1	4

Mwangala PN, *et al. BMJ Open* 2022; 11:e052810. doi: 10.1136/bmjopen-2021-052810
