# Supplementary material for: Mental health and well-being of older adults living with HIV in sub-Saharan Africa: a systematic review
Source: BMJ Open. 2021 Sep 22;11(9):e052810. doi: 10.1136/bmjopen-2021-052810 (PMC8461287; doi:10.1136/bmjopen-2021-052810)
Supplement: Author's manuscript [file bmjopen-2021-052810.draft_revisions.pdf]

BMJ Open

BMJ Open is committed to open peer review. As part of this commitment we make the peer review history of every article we publish publicly available.

When an article is published we post the peer reviewers' comments and the authors' responses online. We also post the versions of the paper that were used during peer review. These are the versions that the peer review comments apply to.

The versions of the paper that follow are the versions that were submitted during the peer review process. They are not the versions of record or the final published versions. They should not be cited or distributed as the published version of this manuscript.

BMJ Open is an open access journal and the full, final, typeset and author-corrected version of record of the manuscript is available on our site with no access controls, subscription charges or pay-per-view fees (http://bmjopen.bmj.com).

If you have any questions on BMJ Open's open peer review process please email info.bmjopen@bmj.com

BMJ Open

A Systematic Review of Health and Wellbeing in older adults living with HIV in sub-Saharan Africa

Journal:	*BMJ Open*
Manuscript ID	bmjopen-2021-052810
Article Type:	Original research
Date Submitted by the Author:	27-Apr-2021

Complete List of Authors:	mwangala, patrick; KEMRI-Wellcome Trust Research Programme, neurosciences<br>Mabrouk, Adam; KEMRI-Wellcome Trust Research Programme, Neurosciences<br>Wagner, Ryan; University of the Witwatersrand, MRC/Wits Rural Public Health and Health Transitions Research Unit (Agincourt), School of Public Health, Faculty of Health Sciences<br>Newton, Charles; Centre for Geographic Medicine Research Coast, Psychiatry, Oxford University<br>Abubakar, Amina A.; Pwani University, Department of Public Health

Keywords:	HIV & AIDS < INFECTIOUS DISEASES, PSYCHIATRY, PUBLIC HEALTH

SCHOLARONE™
Manuscripts

I, the Submitting Author has the right to grant and does grant on behalf of all authors of the Work (as defined in the below author licence), an exclusive licence and/or a non-exclusive licence for contributions from authors who are: i) UK Crown employees; ii) where BMJ has agreed a CC-BY licence shall apply, and/or iii) in accordance with the terms applicable for US Federal Government officers or employees acting as part of their official duties; on a worldwide, perpetual, irrevocable, royalty-free basis to BMJ Publishing Group Ltd ("BMJ") its licensees and where the relevant Journal is co-owned by BMJ to the co-owners of the Journal, to publish the Work in this journal and any other BMJ products and to exploit all rights, as set out in our licence.

The Submitting Author accepts and understands that any supply made under these terms is made by BMJ to the Submitting Author unless you are acting as an employee on behalf of your employer or a postgraduate student of an affiliated institution which is paying any applicable article publishing charge ("APC") for Open Access articles. Where the Submitting Author wishes to make the Work available on an Open Access basis (and intends to pay the relevant APC), the terms of reuse of such Open Access shall be governed by a Creative Commons licence – details of these licences and which Creative Commons licence will apply to this Work are set out in our licence referred to above.

Other than as permitted in any relevant BMJ Author's Self Archiving Policies, I confirm this Work has not been accepted for publication elsewhere, is not being considered for publication elsewhere and does not duplicate material already published. I confirm all authors consent to publication of this Work and authorise the granting of this licence.

For peer review only

A Systematic Review of Health and Wellbeing in older adults living with HIV in sub-Saharan Africa

*Patrick N Mwangala[1,2], Adam Mabrouk[1], Ryan G Wagner[3], Charles R Newton[1, 4,] Amina Abubakar[1, 4 5,6]

Author Affiliations:

[1]Centre for Geographic Medicine Research Coast, Kenya Medical Research Institute (KEMRI), P.O Box 230-80108, Kilifi, Kenya

[2]School of Public Health, University of the Witwatersrand, 27 St Andrews Road, Parktown 2193, South Africa

[3]MRC/Wits Rural Public Health and Health Transitions Research Unit (Agincourt), University of the Witwatersrand, Faculty of Health Sciences, Johannesburg, South Africa

[4]Department of Psychiatry, University of Oxford, Warneford Hospital, Warneford Ln, Oxford OX3 7JX, United Kingdom

[5]Department of Public Health, Pwani University, P.O. BOX 195-80108, Kilifi, Kenya

[6]Institute for Human Development, Aga Khan University, P.O. BOX 30270-00100, Nairobi, Kenya

*Corresponding author: Patrick N Mwangala; Email: pmwangala27@gmail.com; Tel: +254 708 759457

For peer review only

For peer review only - http://bmjopen.bmj.com/site/about/guidelines.xhtml

Abstract

Objectives: To summarise the empirical evidence on common mental disorders (CMDs), cognitive impairment, frailty and health-related quality of life (HRQoL) among people infected with human immunodeficiency virus (HIV) aged ≥50 years (PLWH50+) residing in sub-Saharan Africa (SSA). Specifically, we document the prevalence and correlates of these outcomes.

Methods: A systematic search for publications was conducted in PubMed, CINAHL, PsycINFO, Embase, and Scopus databases. We reviewed articles on CMDs, cognitive impairment, frailty and HRQoL among PLWH50+ living in SSA, published until Jan 2021.

Results: A total of 50 studies from fifteen SSA countries met the inclusion criteria. About two-thirds of these studies emanated from Ethiopia, Uganda, and South Africa. Studies regarding depression predominated (*n=26)* followed by cognitive impairment (*n=13*). Overall, PLWH50+ exhibited varying prevalence of depression (6% to 59%), cognitive impairments (4% to 61%) and frailty (3% to 15%). The correlates of CMDs, cognitive impairment, frailty and HRQoL were rarely investigated, but those reported were sociodemographic variables, many of which were inconsistent.

Conclusions: This review documented an increasing number of published studies on HIV and ageing from SSA. However, we find the current evidence inadequate to characterise the real public health dimension of these impairments in the region, partly because of heterogeneous findings, a limited number of well-designed studies on the subject and substantial methodological limitations in many of the available studies. Future work should have sufficiently large samples of PLWH50+, engage appropriate comparison groups, harmonise the measurement of these outcomes using a standardised methodology to generate more robust prevalence estimates and confirm predictors.

Strengths and Limitations of this study

- This is the first comprehensive review of common mental disorders, cognitive impairment, frailty and quality of life among older adults living with HIV in SSA, synthesising the current evidence regarding the prevalence and correlates of these outcomes.
- Our expanded search of five online databases and snowballing techniques minimised the risk of missing relevant studies.
- Meta-analysis was not conducted due to extensive heterogeneity across the studies in methods and presentation of results.
- We observed substantial methodological limitations in many of the included studies, e.g. small sample sizes, making it difficult to characterise the real public health dimension of these impairments in the region.

For peer review only - http://bmjopen.bmj.com/site/about/guidelines.xhtml

1.0 Background

All societies in the world are experiencing an ageing population – some are in its early stages, and some are more advanced [1]. Likewise, the proportion of people living with HIV (PLWH) aged ≥50 years (considered as older adults within the HIV literature) is growing rapidly across the globe [2], demanding an increase in research, policy and practice to address the complex needs of this vulnerable group [3]. The increase in older PLWH is mainly driven by two factors: effective antiretroviral therapy (ART) and new HIV infections among the elderly [4]. Several ageing HIV cohorts (mostly from high-income countries – HICs) are pointing out an elevated burden of physical conditions (e.g. cardiometabolic diseases), mental morbidities (e.g. depression, cognitive disorders), psychosocial challenges (e.g. stigma, loneliness), and geriatric syndromes (e.g. frailty) among PLWH ≥50 years (PLWH50+) compared to their uninfected counterparts [4-6]. Unfortunately, the mechanisms for the heightened risk of poor outcomes among PLWH50+ are not fully understood but span a host of disease-related factors, legacy of the early years of the HIV epidemic, host vulnerability factors, and treatment factors [4]. The burden and determinants of many of these impairments in SSA, the region most affected by HIV globally, are largely undocumented.

In 2016 there were around 6 million PLWH50+ globally, representing 16% of the entire adult HIV population [2]. This proportion was projected to reach 21% by the end of 2020, with Southern and Eastern Africa containing the vast majority of PLWH50+ [2]. Unfortunately, this population is being neglected in HIV care and prevention efforts, especially in low and middle-income settings of sub-Saharan Africa (SSA) [7]. In SSA, this concern is not just based on the close association between ageing and physical, cognitive, mental impairments, but also on the recognition that older adults are not considered to be a priority for policy issues. They are marginalised and increasingly vulnerable to greater poverty [8, 9]. The growing numbers of PLWH50+ in SSA and the increasing burden of age-associated comorbidities need research to provide evidence to develop targeted interventions to address the unique needs of this vulnerable yet neglected population.

Frailty, common mental disorders (CMDs) such as depression and anxiety, and cognitive impairments are of particular concern in this population [4-6]. Frailty is an important emerging indicator of vulnerability that is increasingly being evaluated among individuals ageing with HIV [10]. It describes a state of physiologic vulnerability that, in the presence of internal or external stressors, puts a person at an elevated risk of adverse clinical outcomes, such as hospitalisation, functional disability, and mortality [11, 12]. Generally, the mental morbidities and frailty are understudied despite their elevated burden and potential adverse impacts on the individuals' HIV treatment and health-related quality of life (HRQoL). The course and implications of these outcomes are well described in HICs, but it is an emergent research area in SSA. However, since the year 2010, the SSA region has witnessed a rise in interest in HIV and ageing outcomes, evidenced by the establishment of cohort studies of older people in different parts of Africa [13-15]. Therefore, the current review aims to summarise the existing empirical evidence on common mental disorders (CMDs), cognitive impairment, frailty, and health-related quality of life (HRQoL) among PLWH50+ in SSA by addressing the following specific objectives.

a) Establish the prevalence of CMDs, cognitive impairment and frailty among PLWH50+ in SSA.

For peer review only - http://bmjopen.bmj.com/site/about/guidelines.xhtml

b) Identify the correlates of CMDs, cognitive impairment, frailty and HRQoL among PLWH50+ in SSA.

2.0 Methods

This systematic review was conducted following the Preferred Reporting Items for Systematic Reviews and Meta-analyses (PRISMA) guidelines [16]. The review protocol was prospectively registered in PROSPERO (Registration number: CRD42020145791).

2.1. Search strategy. Five online databases (PubMed, CINAHL, PsycINFO, Embase, and Scopus) were searched for publications. The last search was conducted on Jan 4th, 2021. The following search terms were used: (HIV OR HIV-1 OR HIV/AIDS OR HIV infections) AND (adult OR older adult OR older people OR older individual OR elderly) AND (Africa OR sub-Saharan Africa OR Africa South of the Sahara) AND (cognitive impairment OR neurocognitive impairment OR neurological complication OR HIV-associated neurocognitive disorder OR common mental disorder OR depression OR depressive symptoms OR depressive disorder OR anxiety OR anxiety disorder OR quality of life OR health-related quality of life OR grip strength OR hand strength OR frailty). We also searched through the reference lists of included articles to access additional articles.

Studies were considered eligible if they: (i) involved older PLWH whose mean/median age was ≥ 50 years or studies that aggregated the outcomes of interest by age (including the ≥ 50 age category); (ii) were conducted within SSA; (iii) were empirical and published in a peer-reviewed journal; (iv) reported any of our outcomes of interest (CMDs, cognitive impairment, frailty or HRQoL). We excluded studies that did not aggregate the outcomes of interest by age and HIV status. Two of the authors (PNM and AM) independently screened the titles, abstracts and full articles for eligibility.

2.2. Data extraction and quality assessment. The two authors (PNM and AM) independently extracted the following study characteristics using a similarly designed extraction sheet: (i) first author; (ii) country and the year of publication; (iii) sample description; (iv) how the outcome of interest was measured; (v) results reported; (vi) key findings on the outcome of interest. In each study, we extracted the specific estimate being reported for each outcome. For studies with mixed HIV infection groups or additional age categories apart from those ≥50 years, we computed the reported estimate of the older adults meeting the age eligibility criteria, i.e. ≥50 years. For studies where it was impossible to do such computations, the burden of the outcomes of interest was reported in their original effect measures.

We evaluated the quality of included studies using the modified version of the Newcastle-Ottawa Scale (NOS) [17]. The scale uses five parameters [*sample size, sample representativeness, comparability between respondents and non-respondents, ascertainment of the outcome of interest, and quality of descriptive statistics reporting*] to obtain a total score ranging from 0 – 5. A score ≥3 is considered a low risk of bias, while a score of <3 is regarded as a high risk of bias.

2.3. Data Synthesis and Analysis. A narrative synthesis approach was used to compare studies and describe the state of evidence on all reported outcomes; by describing the effect estimates, for instance, proportions, means with their standard deviations, and median with their interquartile ranges. Where applicable, ranges were used to summarise the outcomes of

For peer review only - http://bmjopen.bmj.com/site/about/guidelines.xhtml

interest reported. A meta-analysis could not be done due to a limited number of studies and substantial heterogeneity among studies.

2.4. Patient and Public Involvement Statement. The accompanying manuscript is part of broader doctoral research work for the first author. During the conceptual phase of this work, community engagement through meetings with different stakeholders was carried out to contextualise the issues of ageing and HIV at the coast of Kenya (e.g. in terms of priorities and preferences). These stakeholders included the Kilifi and Mombasa counties' departments of Health, sub-county health management teams, and hospital management teams of various health facilities at the study setting. The research team received and incorporated useful feedback from these meetings towards the design and implementation of the project. It also led to further elaborations regarding ongoing community engagement during and after the study, including disseminating any work arising from this project using these forums and others such as publications, hospital departmental meetings, patient groups and conferences as an essential way to discuss the implications of the findings. For this systematic review, patients were not involved in the conduct of the study. However, the results will be disseminated to the relevant patient groups within the HIV clinics through feedback meetings.

3.0 Results

3.1. Characteristics of included studies

We identified 8,741 articles from the 5 online databases and an additional 7 citations from snowballing. Of these, 2,693 were duplicates. Hence, we screened 6,055 titles and abstracts for initial eligibility, out of which 575 articles were identified. Full articles were obtained for these citations, of which 50 met the eligibility criteria (Figure 1). The eligible studies were conducted between 2006 and 2021 among 15 SSA countries of Uganda (n=13), South Africa (n=12), Ethiopia (n=8), Tanzania (n=5), Nigeria (n=3), Cameroon (n=2), Côte d'Ivoire (n=2), Senegal (n=2), Kenya (n=2), Botswana (n=1), Zambia (n=1), Malawi (n=1), Namibia (n=1), Togo (n=1) and Burkina Faso (n=1). Figure 2 shows the geographical distribution of these studies across the SSA region. Virtually all the included studies were cross-sectional, except one that presented data from a randomised trial [18]. Eighteen studies (36%) analysed baseline or follow up data of different cohort studies (Table 1).

About two-thirds of the included studies *(n=35)* recruited their HIV infected samples from clinical settings [18-52], while the rest obtained their samples from the general population through household surveys and community samples. Additionally, many of the studies included samples of PLWH50+ lower than 100 (*n=22*). Overall, the samples of PLWH50+ per study ranged from 19 to 1,048 [36, 53], while those for HIV uninfected older adults ranged from 17 to 4,022 [22, 54].

Common HIV disease markers such as the duration lived with HIV infection, current/nadir CD4+ T cell count, duration of ART use, viral suppression level among PLWH50+ were reported in a few studies. However, there was extensive heterogeneity in how the information was reported and the level of detail given. In 42% of the included studies, all PLWH50+ were on ART. Six other studies included only ART naïve PLWH50+. Among ART users, viral suppression was reported in 5 studies where it was documented to range from 73% - 90%. Nadir CD4 + T cell count among PLWH50+ was reported in 4 studies where most of the

For peer review only - http://bmjopen.bmj.com/site/about/guidelines.xhtml

participants (about 60%) had a count lower than 200. The duration of HIV infection among PLWH50+ ranged from 3.5 to 12 years (among the few studies that reported this variable).

Only eight studies examined the health outcomes of interest using an exclusively HIV infected older sample [25, 33, 40, 45-49]. Eighteen other studies used HIV uninfected older adults as comparison groups, while the rest included HIV infected young adults (the ages of which varied extensively). Details of the characteristics of the included studies are highlighted in Table 1.

Insert Figure 1 – ***Identification of eligible studies*** – here

Insert Figure 2 – ***Geographical distribution of the included studies within SSA*** – here

3.2. Common mental disorders.

Depression was reported in 25 studies, while anxiety was reported in 2 studies (Table 2). Nine of the depression studies [19, 29, 44, 49, 51, 55-58] reported major depressive disorders (MDD), while the remaining sixteen studies reported the prevalence of depressive symptoms. To synthesise and report the available evidence, depression (whenever used in this review) captures both the depressive symptoms (derived from brief screening measures) and Major Depressive Disorder (based on structured diagnostic interviews, e.g. the MINI by specialised professionals or trained non-specialised workers). Overall, the prevalence of depression among PLWH50+ ranged from 8% to 59% (when utilising brief screening tools for depressive symptoms) [27, 59] and from 6% to 30% (when using structured interviews for major depression) [44, 57]. Eight studies documented higher rates of depression among PLWH50+ than their comparison groups [20, 23, 27, 36, 37, 43, 55, 56]. On the contrary, 11 studies reported lower rates of depression among PLWH50+ than their comparison groups [19, 24, 28, 29, 31, 32, 44, 50, 51, 58, 59] while three other studies reported minimal or no differences in depression between PLWH50+ and their comparison groups [34, 42, 57]. In one population-based study conducted in rural South Africa, neither diagnosis nor treatment of HIV was significantly associated with depression when compared with being uninfected [54].

Anxiety was reported in two studies [30, 39]. In one of them [39], PLWH50+ presented with the same proportion of anxiety symptoms with HIV uninfected older adults at 3%. In the other study [30], the prevalence of anxiety disorders among PLWH50+ was 21% compared to 22% in HIV infected young adults (18 – 50 years).

Correlates of depression among PLWH50+ were reported only in 8 studies [27, 28, 46, 48, 51, 54, 55, 58]. Among sociodemographic variables, older age [27, 54], declining socioeconomic status [55], being unemployed [46], being female [28, 58], HIV status disclosure [48], and urban residency [58] were associated with an elevated risk of depression

For peer review only - http://bmjopen.bmj.com/site/about/guidelines.xhtml

while being married, employed (full time or part-time work) [54], old age [51], higher educational attainment [48], and belonging to the highest wealth quintile [28] were associated with reduced levels of depression. However, in some studies, household wealth quintile, education levels [51], and age [25] were not significant correlates of depression. In other studies, being a current/former smoker [46], increasing disability scores, decreasing mean handgrip strength, reported back pain, not having hypertension [55], HIV stigma [48], and caregiving (for adult children) [58] were significantly associated with elevated rates of depression. On the other hand, receiving a government grant [58] and resilience [48] were significantly associated with reduced levels of depression. Details of this data are presented in Table 2.

Insert Table 1 – ***Summary of included studies*** – here

Insert Table 2 - ***Summary of prevalence estimates and correlates for common mental disorders*** – here

3.3. Cognitive and neurological functioning

These outcomes were reported by 13 studies, as highlighted in Table 3. Of these, five studies quantified the prevalence of HIV-associated neurocognitive disorders (HAND) using different measures [25, 35, 38, 40, 49], two studies reported findings on dementia [21, 60] while 5 studies documented findings on cognitive function/impairment [22, 33, 51, 53, 61]. The remaining study reported the proportion of HIV-associated peripheral neuropathy among PLWH50+ [39]. Three HAND studies documented the prevalence of symptomatic HAND in exclusive samples of PLWH50+ and reported impairment of 22% in two studies [25, 49] and 24% [40]. The other two HAND studies documented elevated proportions of symptomatic HAND among PLWH50+ compared to their younger counterparts [35, 38]. In two of the studies reporting cognitive function, PLWH50+ exhibited significantly poorer performance in several domains [22, 51]. In contrast, HIV infected older adults registered lower rates of cognitive impairment (4%) than their uninfected peers (9%) in a baseline analysis of the Health and Aging in Africa: A Longitudinal Study of an INDEPTH Community (HAALSI) population-based cohort in rural South Africa (HAALSI) [53]. In a separate analysis of the same baseline data of the HAALSI cohort [61], HIV infected older adults scored on average 0.06 (95% confidence interval (CI) 0.01–0.12) standard deviation (SD) units higher on a conventional cognitive function measure and 0.02 (95%CI − 0.07 to 0.04) SD units lower when using the Oxford Cognitive Screen-Plus (OCS-Plus) measure than their HIV-negative counterparts. Still in South Africa, Joska and colleagues examined the prevalence of dementia and its associations in a population survey of 1,150 rural elderly participants. They found no significant differences in the rates of neurocognitive impairment between PLWH50+ (18%) and HIV uninfected counterparts (11%) [60]. In the remaining study, 19% of the PLWH50+ were reported to have peripheral neuropathy [39].

The correlates of cognitive function/impairment were reported in 7 studies. The key predictors of reduced cognitive function were mainly sociodemographic: older age (≥50 years) [21, 40, 53, 60], living alone [25], being illiterate or having low formal education level [25, 53], being older at the time of HIV diagnosis [25], being female, unmarried, and having

For peer review only - http://bmjopen.bmj.com/site/about/guidelines.xhtml
For peer review only

low socioeconomic status [53] were associated with a higher risk of impairment. However, in some studies, the effects of age, education, and marital status were not significant [25, 60]. Other correlates of cognitive impairment included the presence of depressive symptoms [60], a history of cardiovascular conditions [53], and a history of falls in the past 12 months [33]. HIV specific factors (such as nadir CD4 count, ART use) [25, 61] and vascular risk factors (such as smoking status, body mass index and blood pressure) [25] were not associated with cognitive impairment.

Insert Table 3 – ***Summary of prevalence estimates and correlates for cognitive impairment/neurological impairment*** – here

For peer review only

For peer review only - http://bmjopen.bmj.com/site/about/guidelines.xhtml

3.4. Frailty/grip strength.

Frailty was reported in three studies [33, 47, 62]. In the first study, a hospital-based cross-sectional study of 145 PLWH50+ in Tanzania, frailty assessment was completed using 3 measures (the Fried frailty phenotype – FFP, the Clinical Frailty Scale – CFS, and Brief Frailty Instrument for Tanzania – B-FIT 2) [47]. In this study, the authors reported low levels of frailty: 3% (when using the FFP method) and 1% (when using the CFS and BFIT-2 methods). The second study documented similar frailty rates among 292 PLWH50+ and 322 HIV uninfected peers in rural South Africa (15% vs 18% respectively), using a modified version of the FFP [62]. In the third study, increasing frailty scores were significantly associated with increasing polypharmacy levels in Uganda [33]. Five other studies [18, 45, 57, 63, 64] reported mean handgrip strength as an indicator of frailty. Among these studies, one reported a higher mean handgrip strength [63] while another reported a weaker mean handgrip strength [57] among PLWH50+ compared to similarly-aged HIV uninfected individuals. In another study, there were no significant differences in mean handgrip strength among PLWH50+ and demographically similar HIV uninfected older adults [64]. In a separate study, a sample of malnourished PLWH50+ taking part in a randomised trial in Zambia and Tanzania recorded continuous improvements in mean handgrip strength at three different time points (time of ART initiation, after 12 weeks, and 2-3 years later) [18].

The correlates of frailty were reported in two studies. Being female, unmarried [47] and older [47, 62] were significantly associated with worse frailty scores, and there were no associations between inflammatory biomarkers with frailty [62]. Factors associated with mean handgrip strength were reported in four of the studies. In one, HIV status was significantly associated with weaker handgrip strength [57]. In the other study, being female and being divorced/widowed were significantly associated with weaker handgrip strength, while having a secondary education level was significantly associated with better handgrip strength. Table 4 gives details of this outcome.

Insert Table 4 – ***Summary of prevalence estimates and correlates for frailty and handgrip strength*** – here

For peer review only - http://bmjopen.bmj.com/site/about/guidelines.xhtml

3.5. Health-related quality of life

This outcome was reported in 8 studies. Two of these studies reported better mean quality of life scores [63, 65] among PLWH50+ than their comparison groups (HIV uninfected older adults). In contrast, four others reported a significantly lower mean quality of life scores [39, 41, 52, 66] among PLWH50+ compared to HIV uninfected older adults or HIV infected young adults. In another study, there were no significant differences in the mean quality of life scores between PLWH50+ and their younger counterparts [26].

The correlates of quality of life were reported in only 1 study [65]. In this study, having some source of income and being in the upper two wealth quintiles were significantly associated with the likelihood of having a better quality of life among PLWH50+. Being female and previously married were associated with reduced odds of good quality of life in PLWH50+ in univariate and multivariate models. Table 5 gives more details on this outcome.

Overall, a significant number of the included studies (about 35%) had a low quality score (≤2 – indicating a high risk of bias) [19-22, 26, 29, 31, 35-41, 48, 51, 66]. Specifically, about two-thirds of the included studies did not report participant response rates. Sixteen studies used non-probability sampling methods or did not report their sampling methods. Close to half of the included studies had substantially small samples of PLWH50+ (≤100). Furthermore, a substantial number of studies (about 40%) provided scarce information regarding their measures' reliability and validity. Details of the quality assessment of all included studies is captured in the Supplementary File.

Insert Table 5 – ***Summary of results for health-related quality of life*** – here

For peer review only

For peer review only - http://bmjopen.bmj.com/site/about/guidelines.xhtml

4.0 Discussion

The evidence informing the interaction of HIV, ageing and health in SSA is slowly growing, with more than two-thirds of the included studies in this review being published between 2015 and 2020. However, our review shows that the research on depression, anxiety, cognitive function, frailty and HRQoL among PLWH50+ in SSA is still scanty and largely concentrated in three countries (Uganda, South Africa and Ethiopia). Overall, we find the current evidence inadequate to characterise the real public health burden of these impairments in the region, partly because of limited formal measurements, heterogeneous findings, a limited number of well-designed studies and substantial methodological limitations observed in many of the included studies. Therefore, any policies and programmes about the wellbeing of PLWH50+ in the region have been made on the basis of minimal evidence. The rising numbers of PLWH50+ in SSA and potentially elevated burden of age-associated comorbidities in this population warrant more research across the region to provide the evidence needed to develop targeted, comprehensive policies, programmes, and interventions to enhance the health outcomes of PLWH50+.

Depression was the most frequently reported outcome in about half of the included studies, followed by cognitive impairment in around a quarter of the studies. This finding conforms with the existing HIV literature, which recognises depression as the most commonly occurring psychiatric complication in this population, hence widely studied [67]. Consistent with the literature [68, 69], screening measures were more likely to report more depressive symptoms than structured diagnostic interviews. This highlights the importance of a two-staged measurement approach where screening is followed by a structured clinical examination to yield more accurate estimates of the mental health burden. Overall, PLWH50+ exhibited varied rates of depression (6% to 59%) [27, 57] and cognitive impairments (4% to 61%) [35, 53]. The variance in the impairments across the studies could reflect major contextual differences across the SSA region in terms of social-environmental milieu, the timing of ART initiation, commonly used ART regimens, the prevalence of co-infections, healthcare systems, formal and informal support systems which are likely to alter the risk profile and morbidity phenotypes of these adults [70]. This observation could also be a consequence of the methodological limitations in some of the included studies. Indeed, about 35% of the studies scored poorly (≤ 2) on the measure of quality, thus indicating a high risk of bias [19-22, 26, 29, 31, 35-41, 48, 51, 66]. The key methodological concerns included small sample sizes of PLWH50+, diverse and unrepresentative samples, failure to report important study details such as sample selection, and validity of the measures used. We also observed considerable heterogeneity in the different outcomes, with different authors using various measures and definitions of their outcomes, thus making it difficult to quantify and compare the burden of reported outcomes. For instance, cognitive impairment was reported in three different ways: HIV-associated neurocognitive disorder, [25, 35, 38, 40, 49], dementia [21, 60] and cognitive dysfunction [22, 33, 53]. Also, some of the included studies had overlapping age groups for their HIV infected young adults (18 – 49, 18 – 50, 18 – 54, 18 – 55, 20 – 49, and 21 – 49), thus making it difficult to compare their outcomes with those of PLWH50+.

Despite the observed limitations, the evidence of better cognition [53, 61], mental health [28, 44, 50, 51, 54, 58, 59], frailty/handgrip strength [47, 62, 63] and quality of life [65] outcomes among PLWH50+ on ART (mainly among the robust studies with low risk of bias) compared

For peer review only - http://bmjopen.bmj.com/site/about/guidelines.xhtml

to uninfected older adults in the current review was striking. This observation could be part of an emerging body of evidence from SSA showing that the mental, cognitive, frailty and quality of life outcomes among PLWH are comparable to those without HIV. The mechanisms responsible for this observation are not well established and require further examination across many settings in SSA. However, it could be attributed to a few plausible reasons, including i) the direct impact of ART on viral replication, inflammatory pathways, and downstream disease pathophysiology, the so-called "ART advantage" [71, 72], ii) a survivorship bias among PLWH50+ who access and remain in HIV care; and iii) beneficial spillover effects of ART programs such as enhanced access to and utilisation of primary care services resulting in early identification and management of comorbid conditions [72-74]. This observation could also be related to the fact that PLWH50+ on ART are more likely to utilise biomedical facilities due to better education and higher socioeconomic status than their HIV uninfected peers. We also recognise the potential effects of Berkson's bias – some form of selection bias that arises when a sample is derived from a subpopulation instead of the general population [75]. More studies, preferably longitudinal ones, will shed more light on this observation. As it stands, however, this finding offers an interesting contrast to the commonly reported findings from high-income countries, which generally show that PLWH50+ present with worse physical and mental health outcomes compared to age-matched HIV uninfected people [5]. In a recent analysis of the AGEHIV Cohort Study in the Netherlands, PLWH50+ (n=541) reported significantly worse physical and mental aspects of HRQoL and had a higher prevalence of depression than HIV-uninfected older individuals (n=526) [76]. Similarly, in the United States, Do and colleagues registered an excess burden of depression (prevalence ratio of 2.90) among HIV infected persons receiving medical care compared to the general population [77]. Likewise, a recent review examining ways of optimising the health and wellbeing of older people living with HIV in the UK reported a higher incidence of comorbidities than in the general population [78]. These findings may suggest context-specific differences in the drivers of physical and mental outcomes, including the demographic and risk factor profile as well as the role of engagement in HIV care – among those with and without HIV, especially when comparing low and middle-income to high-income countries. Future studies should explore the extent to which HIV programs could be promoting the wellbeing of PLWH50+ in SSA compared to HIV uninfected older adults in the general population who are likely to bear the brunt of the prevailing poor health systems and primary care systems in the region.

The most widely used frailty model in HIV-related research is the frailty phenotype (FP) characterised by Fried and colleagues [79]. The FP comprises 5 criteria (weight loss, weak grip strength, exhaustion, slow walking speed, and low physical activity), with frailty defined by the presence of 3 or more criteria. As evidenced in our review, there is hardly any data on frailty among PLWH50+. Only two studies quantified the prevalence of frailty among PLWH50+ with prevalence ranging from 3% to 15% (using the FP method). One of the studies was exclusively conducted among PLWH50+, yielding a low frequency of frailty (3%). In the other study, conducted in rural South Africa, the prevalence of frailty was similar between PLWH50+ and their uninfected counterparts (15% vs 18%, respectively). This finding supports the growing evidence in SSA, which suggest that older on effective ART have similar or better health profiles than their uninfected counterparts, contrary to what has been observed in HICs. At the global stage, the frailty prevalence in SSA appears relatively lower than that of HICs which ranges from 5% to 29% [80]. The population reaching older

For peer review only - http://bmjopen.bmj.com/site/about/guidelines.xhtml

age in many SSA settings may be relatively fit/active, still working, which may be true of PLWH, potentially favouring better physical health in these individuals. Nonetheless, given the limited number of studies on this outcome in SSA, these findings remain preliminary and inconclusive and highlight the urgent need for more studies to shed more light on these observations.

For some time, HRQoL has emerged as a salient indicator of HIV care and an essential target for HIV-related research, aimed at expanding the continuum of service paradigm [81]. This new quality of life frontier has been proposed as the 'fourth 90' to the original '90-90-90' testing and treatment target championed by UNAIDS: to ensure that 90% of people with viral load suppression have good HRQoL [81]. In the current review, research on HRQoL among PLWH50+ was scanty. Thus, little is known on the risk and protective factors of patient-reported health-related quality of life. Further studies should explore the independent effects of ageing-related conditions such as common mental disorders, cognitive impairments and frailty on HRQoL among PLWH50+ in SSA to support healthy ageing even in the context of a chronic illness such as HIV infection.

The correlates of CMDs, cognitive impairment, frailty and HRQoL have been scarcely investigated in SSA. In the current review, the frequently reported correlates were sociodemographic in nature, many of which (e.g. age, formal education levels and wealth status) were inconsistent. However, being female was consistently associated with declining mental health, cognitive function, HRQoL and frailty status. This finding mirrors the results of a recent review of the physical and psychological health of older women residing in SSA, which indicated that women were significantly more likely to report poor health compared with men [82]. This finding highlights the need for adequate representation of both sexes in research studies, incorporating a gender lens in their analyses to understand better the unique health challenges that each gender faces. Further studies are also urgently needed in SSA to identify potential risk and protective factors, especially those that could be modified, to suggest how to best integrate the management of these impairments in HIV care guidelines in SSA. Future studies should explore the influence of HIV-related factors (e.g. nadir CD4 count, adherence, disease stage and ART regimen), psychosocial risk (e.g. loneliness, ageism, stigma, social support, living arrangements) and other comorbidities on the health and wellbeing of PLWH50+. To promote healthy ageing in the context of chronic conditions, such as HIV infection, the psychological and social domains are especially critical as they could be a way to compensate for physiological limitations (that is, multiple chronic conditions) and allow even in the context of disease and disability, to experience optimal quality of life.

4.1 Strengths and Limitations

To our knowledge, this is the first comprehensive review of CMDs, cognitive impairment, frailty and HRQoL among PLWH50+ living in SSA. Our expanded search of 5 online databases and snowballing techniques minimised the risk of missing relevant studies. However, our review should be considered in the context of important limitations. Despite a thorough search strategy, some articles may have been missed. The substantial amount of heterogeneity across the studies in terms of, e.g. study design, operationalisation of age, definitions of outcomes, and measures made comparisons difficult and did not allow a meta-analysis to be done. Furthermore, most of the studies recruited PLWH50+ from HIV clinics

For peer review only - http://bmjopen.bmj.com/site/about/guidelines.xhtml

whose attendees may represent the less-healthy end of the spectrum of service users leading to overestimation of reported outcomes. Conversely, those at the filter end of the cohort may be more able to attend or proactive concerning their wellbeing, leading to underestimation of outcomes. Besides, the substantial methodological limitations observed might have biased the reported findings, hence limiting their external validity.

4.2 Conclusions

This review found an increased reporting of depressive symptoms among PLWH50+ in SSA. However, the research on cognitive function, anxiety disorders, frailty and quality of life in this population is still scanty and mainly concentrated in Uganda, South Africa and Ethiopia. Overall, the current evidence is inadequate to characterise the real public health dimension of these impairments in the region, partly because of the heterogeneous findings, limited number of well-designed studies and significant methodological limitations observed in many of the included studies. Our findings underscore the need to increase the evidence base of these outcomes in the region. Future studies should: a) explicitly target older persons ≥50 years living with HIV, b) carefully select a comparison group and have sufficient sample sizes for adequate statistical testing, c) expand the geographical distribution of these studies in many SSA settings and use adequately standardised survey tools adapted for the region, d) and generate more data on the risk and determinants of these outcomes using a gender lens. There is also an ongoing need for well-designed longitudinal cohort studies to study the temporal patterns of these outcomes in the region.

Contributors: PNM and AA conceptualised and designed the review. PNM and AM reviewed titles, abstracts and full-text papers for eligibility as well as data extraction. PNM wrote the first draft of the manuscript. AM, CN, RW and AA reviewed and edited the prepared manuscript.

Funding

This work was funded by the Wellcome Trust International Master's Fellowship to PNM (Grant number 208283/Z/17/Z). Further funding supporting this work was from 1) the Medical Research Council (Grant number MR/M025454/1) to AA. This award is jointly funded by the UK Medical Research Council (MRC) and the UK Department for International Development (DFID) under MRC/DFID concordant agreement and is also part of the EDCTP2 program supported by the European Union; 2) DELTAS Africa Initiative [DEL-15-003]. The DELTAS Africa Initiative is an independent funding scheme of the African Academy of Sciences (AAS) 's Alliance for Accelerating Excellence in Science in Africa (AESA) and supported by the New Partnership for Africa's Development Planning and Coordinating Agency (NEPAD Agency) with funding from the Wellcome Trust [107769/Z/10/Z] and the UK government. The funders did not have a role in the design and conduct of the study or interpretation of study findings. The views expressed in this publication are those of the author(s) and not necessarily those of AAS, NEPAD Agency, Wellcome Trust or the UK government. For the purpose of Open Access, the author has applied a CC-BY public copyright license to any accepted manuscript version arising from this submission.

For peer review only - http://bmjopen.bmj.com/site/about/guidelines.xhtml

Competing interests: The authors have no potential conflict of interest to declare.

Patient consent: Not required

Data sharing statement: No original data were generated for this study. All data underlying the results are available as part of the article, and no additional source data are required.

Acknowledgement

The authors would like to thank the Director of Kenya Medical Research Institute for granting permission to publish this work

For peer review only

For peer review only - http://bmjopen.bmj.com/site/about/guidelines.xhtml

References

1. United Nations, *World population ageing 2019: Highlights*, in *ST/ESA/SER. A/430*. 2019.
2. Autenrieth, C.S., et al., *Global and regional trends of people living with HIV aged 50 and over: Estimates and projections for 2000–2020.* PloS one, 2018. **13**(11): p. e0207005.
3. O'Brien, K.K., et al., *Research priorities for rehabilitation and aging with HIV: a framework from the Canada-International HIV and Rehabilitation Research Collaborative (CIHRRC).* AIDS Research and Therapy, 2020. **17**: p. 1-13.
4. Sabin, C.A. and P. Reiss, *Epidemiology of ageing with HIV: what can we learn from cohorts?* Aids, 2017. **31**: p. S121-S128.
5. Brennan-Ing, M., J. Ramirez-Valles, and A. Tax, *Aging With HIV: Health Policy and Advocacy Priorities.* Health Education & Behavior, 2021. **48**(1): p. 5-8.
6. Milic, J., et al., *European cohorts of older HIV adults: POPPY, AGE h IV, GEPPO, COBRA and FUNCFRAIL.* European Geriatric Medicine, 2019. **10**(2): p. 247-257.
7. Joint United Nations Programme on HIV/AIDS, *The Gap Report. Geneva, Switzerland: UNAIDS*. 2014.
8. Saka, S., F. Oosthuizen, and M. Nlooto, *national policies and older people's healthcare in Sub-Saharan Africa: a scoping review.* Annals of global health, 2019. **85**(1).
9. Nabalamba, A. and M. Chikoko, *Aging population challenges in Africa*. 2011: African Development Bank, Chief Economist Complex.
10. Brothers, T.D. and K. Rockwood, *Frailty: a new vulnerability indicator in people aging with HIV.* European Geriatric Medicine, 2019. **10**(2): p. 219-226.
11. Gustafson, D., et al., *Frailty and constellations of factors in aging HIV-infected and uninfected women-the women's interagency HIV study.* The Journal of frailty & aging, 2016. **5**(1): p. 43.
12. Levett, T., *Frailty Prevalence and Predictors in Older Adults with HIV*. 2017, University of Brighton.
13. World Health Organization. *Health statistics and information systems: SAGE HIV studies*. 2020 [cited 2020 June 23]; Available from: https://www.who.int/healthinfo/sage/hiv_studies/en/.
14. Gómez-Olivé, F.X., et al., *Cohort profile: health and ageing in Africa: a longitudinal study of an INDEPTH community in South Africa (HAALSI).* International journal of epidemiology, 2018. **47**(3): p. 689-690j.
15. National Institutes of Health. *Ugandan Non-Communicable Diseases and Aging Cohort (UGANDAC)*. 2020 [cited 2020 June 23]; Available from: https://clinicaltrials.gov/ct2/show/NCT02445079.
16. Page, M.J., et al., *PRISMA 2020 explanation and elaboration: updated guidance and exemplars for reporting systematic reviews.* BMJ, 2021. **372**.
17. Stang, A., *Critical evaluation of the Newcastle-Ottawa scale for the assessment of the quality of nonrandomised studies in meta-analyses.* European journal of epidemiology, 2010. **25**(9): p. 603-605.
18. Filteau, S., et al., *Nutritional status is the major factor affecting grip strength of African HIV patients before and during antiretroviral treatment.* Trop Med Int Health, 2017. **22**(10): p. 1302-1313.
19. Akena, D., et al., *The association between aids related stigma and major depressive disorder among HIV-positive individuals in Uganda.* PLoS One, 2012. **7**(11): p. e48671.
20. Asangbeh, S.L., et al., *Predictors of depression among patients on art in a rural health district in North West Cameroon.* AIDS Care, 2016. **28**(2): p. 205-8.
21. Atashili, J., et al., *Prevalence, characteristics and correlates of a positive-dementia screen in patients on antiretroviral therapy in Bamenda, Cameroon: a cross-sectional study.* BMC Neurol, 2013. **13**: p. 86.
22. Cassimjee, N. and P.K. Motswai, *Neuropsychological profiles of adults and older adults with HIV.* South African Journal of Psychology, 2017. **47**(1): p. 35-45.
23. Cholera, R., et al., *Depression and Engagement in Care Among Newly Diagnosed HIV-Infected Adults in Johannesburg, South Africa.* AIDS & Behavior, 2017. **21**(6): p. 1632-1640.

For peer review only - http://bmjopen.bmj.com/site/about/guidelines.xhtml

24. Duko, B., et al., *Prevalence and associated factors of depression among patients with HIV/AIDS in Hawassa, Ethiopia, cross-sectional study.* Annals of General Psychiatry, 2018. **17**.
25. Eaton, P., et al., *Risk factors for symptomatic HIV-associated neurocognitive disorder in adults aged 50 and over attending a HIV clinic in Tanzania.* Int J Geriatr Psychiatry, 2020.
26. Harding, R., et al., *Quality of life and wellbeing among HIV outpatients in East Africa: a multicentre observational study.* BMC Infect Dis, 2014. **14**: p. 613.
27. Kaharuza, F.M., et al., *Depression and CD4 cell count among persons with HIV infection in Uganda.* AIDS Behav, 2006. **10**(4 Suppl): p. S105-11.
28. Manne-Goehler, J., et al., *Depressive symptoms before and after antiretroviral therapy initiation among older-aged individuals in rural Uganda.* AIDS and Behavior, 2019. **23**(3): p. 564-571.
29. Musinguzi, K., et al., *Association between major depressive disorder and pro-inflammatory cytokines and acute phase proteins among HIV-1 positive patients in Uganda.* BMC Immunol, 2018. **19**(1): p. 1.
30. Olagunju, A.T., et al., *Factors associated with anxiety disorders among HIV-positive attendees of an HIV clinic in Lagos, Nigeria.* Int J STD AIDS, 2012. **23**(6): p. 389-93.
31. Olley, B.O., et al., *Psychosocial factors predicting severity of depression among treatment-seeking HIV/AIDS patients: A multi-site Nigerian study.* Niger J Clin Pract, 2017. **20**(3): p. 296-302.
32. Shumba, C., et al., *Prevalence of Depressive Symptoms Amongst Highly Active Antiretroviral Therapy (HAART) Patients in AIDSRelief Uganda.* J Public Health Afr, 2013. **4**(2): p. e19.
33. Ssonko, M., et al., *Polypharmacy among HIV positive older adults on anti-retroviral therapy attending an urban clinic in Uganda.* BMC Geriatr, 2018. **18**(1): p. 125.
34. Tesfaw, G., et al., *Prevalence and correlates of depression and anxiety among patients with HIV on-follow up at Alert Hospital, Addis Ababa, Ethiopia.* BMC Psychiatry, 2016. **16**(1): p. 368.
35. Tsegaw, M., et al., *Screening HIV-associated neurocognitive disorders (HAND) among HIV positive patients attending antiretroviral therapy in South Wollo, Ethiopia.* J Psychiatr Res, 2017. **85**: p. 37-41.
36. Berhe, H. and A. Bayray, *Prevalence of depression and associated factors among people living with HIV/AIDS in tigray, North Ethiopia: A cross sectional hospital based study.* International Journal of Pharmaceutical Sciences and Research, 2013. **4**(2): p. 765.
37. Eshetu, D.A., et al., *Prevalence of depression and associated factors among HIV/AIDS Patients attending ART Clinic at Debrebirhan referral hospital, North Showa, Amhara Region, Ethiopia.* Clin Psychiatry, 2015. **1**(1): p. 1-7.
38. Mugendi, A., et al., *Prevalence and Correlates of Neurocognitive Disorders among HIV patients on antiretroviral therapy at a Kenyan Hospital.* Neurology research international, 2019. **2019**.
39. Obimakinde, A.M., et al., *Going Beyond Giving Antiretroviral Therapy: Multimorbidity in Older People Aging with HIV in Nigeria.* AIDS research and human retroviruses, 2020. **36**(3): p. 180-185.
40. Oumar, G., et al., *Neurocognitive Disorders in People Living with Human Immunodeficiency Virus Aged 50 Years Old and More in Yalgado Ouédraogo Teaching Hospital.* American Journal of Internal Medicine, 2020. **8**(4): p. 177-181.
41. Parcesepe, A.M., et al., *Gender, HIV-related stigma, and health-related quality of life among adults enrolling in HIV Care in Tanzania.* AIDS and Behavior, 2020. **24**(1): p. 142-150.
42. Torgersen, J., et al., *Impact of efavirenz metabolism on loss to care in older HIV+ Africans.* European Journal of Drug Metabolism and Pharmacokinetics, 2019. **44**(2): p. 179-187.
43. Abadiga, M., *Depression and its associated factors among HIV/AIDS patients attending ART clinics at Gimbi General hospital, West Ethiopia, 2018.* BMC Research Notes, 2019. **12**(1): p. 527.
44. Asrat, B., et al., *Major depressive disorder and its association with adherence to antiretroviral therapy and quality of life: cross-sectional survey of people living with HIV/AIDS in Northwest Ethiopia.* BMC psychiatry, 2020. **20**(1): p. 1-13.

For peer review only - http://bmjopen.bmj.com/site/about/guidelines.xhtml

45. Bernard, C., et al., *Prevalence and factors associated with physical function limitation in older West African people living with HIV.* PloS one, 2020. **15**(10): p. e0240906.
46. Bernard, C., et al., *Prevalence and factors associated with severe depressive symptoms in older west African people living with HIV.* BMC psychiatry, 2020. **20**(1): p. 1-11.
47. Bristow, C., et al., *Low levels of frailty in HIV-positive older adults on antiretroviral therapy in northern Tanzania.* Journal of NeuroVirology, 2021: p. 1-12.
48. Kalomo, E.N., et al., *HIV stigma, resilience and depressive symptoms among older adults living with HIV in rural Namibia.* African Journal of AIDS Research, 2020. **19**(3): p. 198-205.
49. Kellett-Wright, J., et al., *Screening for HIV-Associated Neurocognitive Disorder (HAND) in Adults Aged 50 and Over Attending a Government HIV Clinic in Kilimanjaro, Tanzania. Comparison of the International HIV Dementia Scale (IHDS) and IDEA Six Item Dementia Screen.* AIDS and Behavior, 2020: p. 1-12.
50. Motumma, A., et al., *Prevalence and associated factors of common mental disorders among adult patients attending HIV follow up service in Harar town, Eastern Ethiopia: a cross-sectional study.* BMC psychology, 2019. **7**(1): p. 1-9.
51. Moucheraud, C., et al., *A Multi-Dimensional Characterisation of Aging and Wellbeing Among HIV-Positive Adults in Malawi.* AIDS and Behavior, 2020: p. 1-11.
52. Yaya, I., et al., *Health-related quality of life among people living with HIV/AIDS in Togo: individuals and contextual effects.* BMC Research Notes, 2019. **12**(1): p. 140.
53. Kobayashi, L.C., et al., *Cognitive function and impairment in older, rural south African adults: evidence from "health and aging in Africa: a longitudinal study of an INDEPTH Community in Rural South Africa".* Neuroepidemiology, 2019. **52**(1-2): p. 32-40.
54. Geldsetzer, P., et al., *Depressive symptoms and their relation to age and chronic diseases among middle-aged and older adults in rural South Africa.* The Journals of Gerontology: Series A: Biological Sciences and Medical Sciences, 2019. **74**(6): p. 957-963.
55. Kinyanda, E., et al., *Risk of major depressive disorder among older persons living in HIV-endemic central and southwestern Uganda.* AIDS Care, 2016. **28**(12): p. 1516-1521.
56. Mugisha, J.O., et al., *Chronic disease, risk factors and disability in adults aged 50 and above living with and without HIV: findings from the Wellbeing of Older People Study in Uganda.* Glob Health Action, 2016. **9**: p. 31098.
57. Negin, J., et al., *Prevalence of HIV and chronic comorbidities among older adults.* Aids, 2012. **26 Suppl 1**(0 1): p. S55-63.
58. Nyirenda, M., et al., *Prevalence and correlates of depression among HIV-infected and-affected older people in rural South Africa.* Journal of affective disorders, 2013. **151**(1): p. 31-38.
59. Rohr, J.K., et al., *HIV treatment cascade for older adults in rural South Africa.* Sexually Transmitted Infections, 2020. **96**(4): p. 271-276.
60. Joska, J.A., et al., *Prevalence of HIV-1 Infection in an elderly rural population and associations with neurocognitive impairment.* Aids, 2019. **33**(11): p. 1765-1771.
61. Asiimwe, S.B., et al., *Cognitive differences associated with HIV serostatus and antiretroviral therapy use in a population-based sample of older adults in South Africa.* Scientific reports, 2020. **10**(1): p. 1-14.
62. Edwards, A., et al., *HIV serostatus, inflammatory biomarkers and the frailty phenotype among older people in rural KwaZulu-Natal, South Africa.* African Journal of AIDS Research, 2020. **19**(3): p. 177-185.
63. Mugisha, J., et al., *Caregiving responsibilities and burden among older people by HIV status and other determinants in Uganda.* AIDS Care, 2013. **25**(11): p. 1341-8.
64. Scholten, F., et al., *Health and functional status among older people with HIV/AIDS in Uganda.* BMC Public Health, 2011. **11**: p. 886.
65. Nyirenda, M., et al., *An investigation of factors associated with the health and well-being of HIV-infected or HIV-affected older people in rural South Africa.* BMC Public Health, 2012. **12**(1): p. 259-259.
66. Maniragaba, F., et al., *Predictors of quality of life of older persons in rural Uganda: A cross sectional study.* AAS Open Res, 2018. **1**: p. 22.

For peer review only - http://bmjopen.bmj.com/site/about/guidelines.xhtml

67. Nanni, M.G., et al., *Depression in HIV infected patients: a review.* Current psychiatry reports, 2015. **17**(1): p. 530.
68. Tsai, A.C., *Reliability and validity of depression assessment among persons with HIV in sub-Saharan Africa: systematic review and meta-analysis.* Journal of acquired immune deficiency syndromes (1999), 2014. **66**(5): p. 503.
69. Ayano, G., M. Solomon, and M. Abraha, *A systematic review and meta-analysis of epidemiology of depression in people living with HIV in east Africa.* BMC psychiatry, 2018. **18**(1): p. 254.
70. Nyirenda, M., et al., *Health, wellbeing, and disability among older people infected or affected by HIV in Uganda and South Africa.* Global health action, 2013. **6**(1): p. 19201.
71. Pala, A.N., et al., *Subtypes of depressive symptoms and inflammatory biomarkers: An exploratory study on a sample of HIV-positive patients.* Brain, behavior, and immunity, 2016. **56**: p. 105-113.
72. Manne-Goehler, J., et al., *The ART advantage: healthcare utilisation for diabetes and hypertension in rural South Africa.* Journal of acquired immune deficiency syndromes (1999), 2017. **75**(5): p. 561.
73. Feinstein, M.J., et al., *Ideal cardiovascular health and carotid atherosclerosis in a mixed cohort of HIV-infected and uninfected Ugandans.* AIDS research and human retroviruses, 2017. **33**(1): p. 49-56.
74. Wouters, E., et al., *Physical and emotional health outcomes after 12 months of public-sector antiretroviral treatment in the Free State Province of South Africa: a longitudinal study using structural equation modelling.* BMC Public Health, 2009. **9**(1): p. 103.
75. Schwartzbaum, J., A. Ahlbom, and M. Feychting, *Berkson's bias reviewed.* European journal of epidemiology, 2003. **18**(12): p. 1109-1112.
76. Langebeek, N., et al., *Impact of comorbidity and ageing on health-related quality of life in HIV-positive and HIV-negative individuals.* Aids, 2017. **31**(10): p. 1471-1481.
77. Do, A.N., et al., *Excess burden of depression among HIV-infected persons receiving medical care in the United States: data from the medical monitoring project and the behavioral risk factor surveillance system.* PloS one, 2014. **9**(3).
78. Date, H.L., *Optimising the health and wellbeing of older people living with HIV in the United Kingdom.* Acute pain, 2019. **10**: p. 00.
79. Fried, L.P., et al., *Frailty in older adults: evidence for a phenotype.* The Journals of Gerontology Series A: Biological Sciences and Medical Sciences, 2001. **56**(3): p. M146-M157.
80. Levett, T.J., et al., *Systematic review of prevalence and predictors of frailty in individuals with human immunodeficiency virus.* Journal of the American Geriatrics Society, 2016. **64**(5): p. 1006-1014.
81. Lazarus, J.V., et al., *Beyond viral suppression of HIV–the new quality of life frontier.* BMC medicine, 2016. **14**(1): p. 94.
82. Mangipudi, S., T. Cosco, and S. Harper, *A systematic review of physical and psychological health and wellbeing of older women in Sub-Saharan Africa.* Journal of Public Health, 2020. **42**(2): p. 294-303.

For peer review only - http://bmjopen.bmj.com/site/about/guidelines.xhtml

Table 1: Summary of included studies

Author (Year)	Country	Total Sample size	Sample of PLWH50+ (%)	Comparison group sample	Treatment status of PLWH	ART Regimen & other HIV disease markers for PLWH50+	Sample source	Study setting	Study design	Sampling method	Outcomes reported
Abadiga, M. (2019). [43]	Ethiopia	393	55 (14.0)	338 PLWH (18-48) years	All on ART	NR	HIV clinic	Rural – 41.1%; Urban – 43.0%	Cross-sectional	Simple random	Depressive symptoms
Akena, D., et al (2012) [19]	Uganda	368	51 (13.9)	317 PLWH (18-49 years)	NR	NR	HIV clinic	NR	Cross-sectional	Random	Major depressive disorder
Asangbeh, S. L., et al (2016) [20]	Cameroon	202	62 (30.7)	140 PLWH (21-49 years)	All on ART	NR	HIV clinic	Rural	Cross-sectional	NR	Depressive symptoms
Asiimwe, S. B., et al. (2020). [61]	South Africa	4560	1048 (22.9)	3512 HIV uninfected older adults (≥40 years)	68.6% on ART	1499 Mean viral load among ART users and 49034 in non-ART users	Population based	Rural	Population based	Simple random	Cognitive function
Asrat, B., et al. (2020). [44]	Ethiopia	391	54 (13.8)	337 PLWH (18-49) years	All on ART	74.6% on 1st Line ART; 25.4% on 2nd line ART	HIV clinic	NR	Cross-sectional	Systematic random	Major depressive disorder
Atashili, J., et al. (2013) [21]	Cameroon	400	NR	PLWH (20-49 years)	All on ART	NR	HIV clinic	Mixed (Urban and rural)	Cross-sectional	Consecutive	Positive-dementia screen
Berhe, H. and A. Bayray (2013) [36]	Ethiopia	269	19 (7.1)	250 PLWH (18-54 years)	NR	NR	HIV clinics	Mixed (79.2% urban)	Cross-sectional	NR	Depressive symptoms
Bernard, C., et al. (2020). [45]	Coˆte d'Ivoire & Senegal	333	333 (100.0)	None	All on ART	87.1% with undetectable viral load. 48.5% having <500 CD4+	3 HIV clinics within a hospital	Urban	Cross-sectional	Convenience	Grip strength
Bernard, C., et al. (2020). [46]	Coˆte d'Ivoire & Senegal	334	334 (100.0)	None	All on ART	87.1% with undetectable viral load. 48.5% having <500 CD4+	3 HIV clinics within a hospital	Urban	Cross-sectional	Convenience	Sever depressive symptoms
Bristow, C., et al. (2021). [47]	Tanzania	145	145 (100.0)	None	99.3% on ART.	77.2% on 1st line. 72.6% virally suppressed. Mean CD4+ of 494.8. Median time with HIV= 10 years	HIV clinic within a hospital	NR	Cross-sectional	Systematic sampling	Frailty
Cassimjee, N. and P. K. Motswai (2017) [22]	South Africa	50	33 (66.0)	17 HIV uninfected adults (mean age 52.2. years)	NR	Mean years lived with HIV = 3.5 years. Mean CD4+ count of 415. 29.0% with CD4+ cell count <200	HIV wellness centre in a semi-urban community clinic	Semi-urban	Cross-sectional	NR	Neuropsychological profiles
Cholera, R., et al. (2017) [23]	South Africa	340	28 (8.2)	312 PLWH (18-49 years)	ART naïve	ART naïve	HIV primary care clinic	Mixed	Cross-sectional	Random	Depressive symptoms
Duko, B., et al. (2018) [24]	Ethiopia	383	31 (8.1)	352 PLWH (18-54 years)	93.2% on ART	NR	HIV clinic	Mixed (88.8% urban)	Cross-sectional	Systematic random	Depressive symptoms

For peer review only - http://bmjopen.bmj.com/site/about/guidelines.xhtml

Author (Year)	Country	Total Sample size	Sample of PLWH50+ (%)	Comparison group sample	Treatment status of PLWH	ART Regimen & other HIV disease markers for PLWH50+	Sample source	Study setting	Study design	Sampling method	Outcomes reported
Eaton, P., et al. (2020) [25]	Tanzania	253	253 (100.0)	None	94.8% on ART	Majority on 1st line. Median nadir CD4+ cell count of 201.5	HIV clinic within a hospital	NR	Cross-sectional	Systematic random	HAND and depressive symptoms
Edwards, A., et al. (2020). [62]	South Africa	614 (62.5% female)	292 (47.6)	322 HIV uninfected older adults (≥50 years)	NR	NR	Community based sample	Rural	Cross-sectional	Stratified Random sampling	Frailty
Eshetu, D. A., et al. (2015) [37]	Ethiopia	416	60 (14.4)	356 PLWH (20-49 years)	56.2% on ART	NR	HIV clinic	NR	Cross-sectional	NR	Depressive symptoms
Filteau, S., et al. (2017) [18]	Zambia and Tanzania	1742 at baseline	NR	PLWH (18 to 49 years)	ART naïve at baseline	NR	HIV clinics within hospitals	Mixed	Multicenter Randomised trial	Random	Handgrip strength
Geldsetzer, P., et al. (2019) [54]	South Africa	5,059	1,037 (20.5)	4,022 HIV uninfected older adults (≥40 years)	63.9% on ART	NR	Population-based sample	Rural	Population-based survey	Simple random	Depressive symptoms and its associations with chronic diseases
Harding, R., et al. (2014) [26]	Kenya and Uganda	1337	NR	PLWH (18 to 49 years)	49.9% on ART	NR	HIV clinics	NR	cross-sectional	Consecutive	Quality of life
Joska, J. A., et al. (2019) [60]	South Africa	1,150	55 (4.8)	1,095 HIV uninfected older adults ≥60 years	86.1% on ART	NR	Population-based sample	Rural	Population-based cross-sectional	Random	Point prevalence of dementia
Kaharuza, F. M., et al. (2006) [27]	Uganda	1,017	94 (9.2)	923 PLWH (18 to 50 years)	ART naïve	ART naïve	HIV clinic	Rural	Cross-sectional	Random	Depressive symptoms
Kalomo, E. N., et al. (2020). [48]	Namibia	147 (63% females)	147 (100.0)	None	All on ART	NR	2 HIV clinics within a hospital	Rural	Cross-sectional	Purposive and convenience	Depressive symptoms
Kellett-Wright, J., et al. (2020). [49]	Tanzania	235	253 (100.0)	None	95.5% on ART	Majority on 1st line. Median time with HIV =7.1 years. Median CD4+ count of 516.	HIV clinic within a hospital	NR	Cross-sectional	Systematic random	HAND
Kinyanda, E., et al. (2016) [55]	Uganda	468	244 (52.1)	224 HIV uninfected older adults ≥50 years	9.8% on ART	NR	Community cohort of old people	Mixed (rural and periurban)	Cross-sectional	Random	Risk of major depressive disorder
Kobayashi, L. C., et al. (2019) [53]	South Africa	5,059	1,048 (20.7)	4,011 HIV uninfected older adults ≥50 years	63.9% on ART	NR	Population-based sample	Rural	Population-based survey	Simple random	Cognitive impairment
Maniragaba, F., et al. (2018) [66]	Uganda	912	28 (3.1)	884 HIV uninfected older adults ≥60 years	NR	NR	Community-based sample	Rural	Cross-sectional survey	Stratified cluster sampling	Quality of life

For peer review only - http://bmjopen.bmj.com/site/about/guidelines.xhtml

Author (Year)	Country	Total Sample size	Sample of PLWH50+ (%)	Comparison group sample	Treatment status of PLWH	ART Regimen & other HIV disease markers for PLWH50+	Sample source	Study setting	Study design	Sampling method	Outcomes reported
Manne-Goehler, J., et al. (2019) [28]	Uganda	296	154 (52.0)	142 HIV uninfected older adults ≥40 years	All on ART	60.0% on AZT/3TC/NVP Mean CD4+ count of 510; mean nadir CD4+ count of 136. 90.0% virally suppressed.	HIV clinic (for PLWH) and surrounding community (controls)	Rural	Cross-sectional	Convenience sampling	Depressive symptoms
Motumma, A., et al. (2019). [50]	Ethiopia	420	122 (29.0)	298 PLWH (18 - 40 years)	All on ART	NR	2 HIV clinics	NR	Cross-sectional	Simple random	Common mental disorders
Moucheraud, C., et al. (2020) [51]	Malawi	134	74 (55.2)	60 HIV infected young adults (30-49) years	All on ART for ≥ 2 years	58.1% on TDF/3TC/DTG. 76.4% virally suppressed.	HIV treatment program	Urban	Cross-sectional	NR	Depressive symptoms and cognitive functioning
Mugendi, A., et al. (2019) [38]	Kenya	345	64 (18.6)	281 PLWH (20-49 years)	All on ART	NR	HIV clinic	Urban	Cross-sectional	Convenience sampling	Neurocognitive impairments
Mugisha, J., et al. (2013) [63]	Uganda	509	198 (38.9)	311 HIV uninfected older adults ≥50 years	51.0% on ART	NR	Community sample and HIV clinics	Mixed (rural and periurban).	Cross-sectional	Random	Handgrip strength and quality of life
Mugisha, J. O., et al. (2016) [56]	Uganda	471	244(51.8)	227 HIV uninfected older adults ≥50 years	90.6% on ART	NR	Community sample and HIV clinics	NR	Cross-sectional	Random	Depression
Musinguzi, K., et al. (2018) [29]	Uganda	201	21 (10.4)	180 PLWH (18 to 49 years)	ART naïve	ART naïve	HIV clinics	Mixed (semi-urban and rural)	Cross-sectional	Simple random	Major depressive disorder
Negin, J., et al. (2012) [57]	South Africa	2,864	142 (5.0)	2,722 HIV uninfected older adults	NR	NR	Population-based	Mixed (rural and urban)	Cross-sectional	Random	Depression, handgrip strength
Nyirenda, M., et al. (2013) [58]	South Africa	422	203 (48.1)	219 HIV uninfected older adults ≥50 years	23.7% on ART	NR	Population-based sample	Mixed (rural and urban)	Cross-sectional	Stratified random	Depression and its correlates
Nyirenda, M., et al. (2012) [65]	South Africa	422	203 (48.1)	219 HIV uninfected older adults ≥50 years	23.7% on ART	NR	Population-based sample	Mixed (rural and urban)	Cross-sectional	Stratified random	Quality of life
Nyirenda, M., et al. (2013) [70]	Uganda and South Africa	932	400 (43.0)	732 (HIV uninfected older adults ≥50 years	About 22.0% on ART	NR	Population-based sample	Periurban/urban and rural	Multi-site cross-sectional	Random	Quality of life
Obimakinde, A. M., et al. (2020) [39]	Nigeria	186	62 (33.3)	162 HIV uninfected older adults ≥60 years	All on ART	83.9% on 1st Line ART.	HIV clinic and geriatric centre	Urban	Cross-sectional	NR	Anxiety, peripheral neuropathy and quality of life
Olagunju, A. T., et al. (2012) [30]	Nigeria	300	24 (8.0)	276 PLWH (18-50 years)	All on ART	NR	HIV clinic	NR	Cross-sectional	Systematic random	Anxiety disorders

For peer review only - http://bmjopen.bmj.com/site/about/guidelines.xhtml

**Author (Year)**	**Country**	**Total Sample size**	**Sample of PLWH50+ (%)**	**Comparison group sample**	**Treatment status of PLWH**	**ART Regimen & other HIV disease markers for PLWH50+**	**Sample source**	**Study setting**	**Study design**	**Sampling method**	**Outcomes reported**
Olley, B. O., et al. (2017) [31]	Nigeria	502	44 (8.8)	458 PLWH (18 to 50 years)	All on ART	NR	HIV clinics	NR	Cross-sectional	Convenience	Depressive symptoms
Oumar, G., et al. (2020) [40]	Burkina Faso	102	102 (100.0)	None	All on ART	79.4% undetectable viral load. 57.9% with nadir CD4+ cell count of <200	HIV clinic within a hospital	NR	Cross-sectional	NR	Neurocognitive Disorders
Parcesepe, M., et al. (2020) [41]	Tanzania	906	82 (9.1)	824 PLWH (18 to 50 years)	ART naïve	ART naïve	HIV clinics	NR	Cross-sectional	NR	Quality of life
Rohr, J. K., et al. (2020). [59]	South Africa	4560	1048 (23.0)	3512 HIV uninfected older adults (≥40 years)	68.6% (on ART)	1499 Mean viral load among ART users and 49034 in non-ART users	Population-based	Rural	Population based cross-sectional	Simple random	Depressive symptoms
Scholten, F., et al. (2011) [64]	Uganda	500	189 (37.8)	311 HIV uninfected older adults ≥50 years	51.0% on ART	NR	Population-based sample	Peri-urban and rural	Cross-sectional	Random	Handgrip strength
Ssonko, M., et al. (2018) [33]	Uganda	411	411 (100.0)	None	All on ART	NR	HIV clinic	Urban	Cross-sectional	Systematic random	Frailty
Shumba, C., et al. (2013) [32]	Uganda	675	91 (13.5)	584 PLWH (19 to 49 years)	All on ART	NR	HIV clinic	Rural	Cross-sectional	Random	Depressive symptoms
Tesfaw, G., et al. (2016) [34]	Ethiopia	417	59 (14.1)	358 PLWH (19 to 49 years)	All on ART	NR	HIV clinic	NR	Cross-sectional	Systematic sampling	Depressive symptoms
Torgersen, J., et al. (2019) [42]	Botswana	914	125 (13.7)	789 PLWH (21 to 49)	ART naïve	ART naïve	HIV clinics	NR	Cross-sectional	NR	Depression
Tsegaw, M., et al. (2017) [35]	Ethiopia	593	67 (11.3)	526 PLWH (18 to 55 years)	All om ART	94.8% 1st Line ART; rest 2nd line	HIV clinic	NR	Cross-sectional	NR	HAND
Yaya, I., et al. (2019). [52]	Togo	880 (78.4% female)	154 (17.5)	726 HIV infected young adults [15-49] years	90.5% on ART.	90.8% on 1st Line; 9.2% on 2nd line	5 HIV healthcare centres	62.6% - Urban	Cross-sectional	Simple random	Quality of life

Notes: ART – *Antiretroviral treatment*; **AZT –** *Zidovudine*; **CD4+ –***Cluster of Differentiation 4*; **DTG –** *Dolutegravir*; **HIV –** *Human immunodeficiency virus;* **HAND –** *HIV-associated neurocognitive disorders*; **3TC –** *Lamivudine*; **NR –** *Not reported*; **NVP –** *Nevirapine*; **PLWH –** *People living with HIV*; **PLWH50+ –** *People living with HIV ≥50 years old*; **TDF –** *Tenofovir disoproxil*

For peer review only

Table 2: Summary of prevalence estimates and correlates for common mental disorders

Author (Year)	Outcome of interest	Sample size	Assessment tool used	Cut-off score	Information on local tool validation	Prevalence estimates/other results	Correlates reported
Abadiga, M. (2019). [43]	Prevalence of depressive symptoms	55 PLWH48+; 338 PLWH (18-48 years)	9-Item Patient Health Questionnaire	≥5	Previously validated in Ethiopia	49.1% among PLWH48+; 40.5% in PLWH (18-48 years)	Reported correlates not aggregated by age.
Akena, D., et al. (2012) [19]	Prevalence of major depressive disorder	51 PLWH50+; 317 PLWH (18-49 years)	Mini International Neuropsychiatric Inventory	≥5 of the 9 DSM-IV-TR symptoms for major depression	Not locally validated	11.8% among PLWH50+; 18.3% in PLWH (18-49 years)	Reported correlates not aggregated by age.
Asangbeh, S. L., et al. (2016) [20]	Prevalence of depressive symptoms	62 PLWH50+; 140 PLWH (21-49 years)	9-Item Patient Health Questionnaire	≥10	Validated in a previous study in Cameroon	37.0% in PLWH50+; 25.0% in PLWH (21-49 years)	Reported correlates not aggregated by age.
Asrat, B., et al. (2020). [44]	Prevalence of major depressive disorder	54 PLWH50+; 337 PLWH (18-49 years)	Mini International Neuropsychiatric Interview	NR	Previously adapted in Ethiopia	29.6% in PLWH50+; 33.6% in PLWH (40-49 years); 33.5% in PLWH (30-39) years and 31.4% in PLWH (18-29) years.	Reported correlates not aggregated by age.
Berhe, H. and A. Bayray (2013) [36]	Prevalence of depressive symptoms	19 PLWH50+; 250 PLWH (18-54 years)	21-Item Hamilton Depression Scale	≥8	NR	57.9% among PLWH50+; 42.8% in PLWH (18-54 years)	Reported correlates not aggregated by age.
Bernard, C., et al. (2020). [46]	Prevalence and factors associated with severe depressive symptoms	334 PLWH50+	Center for Epidemiological Studies Depression scale (CES-D)	A total score ≥ 17 for men and ≥ 23 for women	Relied on previous validation of the tool from other African countries (Uganda, South Africa and Zambia)	The prevalence of severe depressive symptoms was 17.9% (95% CI: 13.8-22).	PLWH with severe depressive symptoms were more likely to be **unemployed** (AOR = 2.8; 95% CI: 1.4–5.7) and to be **current or former tobacco smokers** (AOR = 2.6; 95% CI: 1.3–5.4) but **were less likely to be overweight or obese** (AOR = 0.4; 95% CI: 0.2–0.8).
Cholera, R., et al. (2017) [23]	Prevalence of depressive symptoms	28 PLWH50+; 312 PLWH (18-49 years)	9-Item Patient Health Questionnaire	≥10	Previously validated against the MINI yielding 78.7% sensitivity and 83.4% specificity in the study population	46.4% among PLWH50+; 28.8% in PLWH (18-49 years)	NR
Duko, B., et al. (2018) [24]	Prevalence of depressive symptoms	31 PLWH50+; 352 PLWH (18-54 years)	9-Item Patient Health Questionnaire	≥11	Previously validated yielding 86% sensitivity and 67% specificity	45.2% among PLWH50+; 48.9% in PLWH (18-54years)	Reported correlates not aggregated by age.
Eaton, P., et al. (2020) [25]	Prevalence of depressive symptoms	253 PLWH50+	15-item Geriatric depression scale	≥5	NR	17.4%	Not reported

For peer review only - http://bmjopen.bmj.com/site/about/guidelines.xhtml

Author (Year)	Outcome of interest	Sample size	Assessment tool used	Cut-off score	Information on local tool validation	Prevalence estimates/other results	Correlates reported
Eshetu, D. A., et al. (2015) [37]	Prevalence of depressive symptoms	60 PLWH50+; 356 PLWH (20-49 years)	9-Item Patient Health Questionnaire	≥5	Sensitivity of 88% and Specificity of 88%	46.7% among PLWH50+; 37.6% in PLWH (20-49 years)	Reported correlates not aggregated by age
Geldsetzer, P., et al. (2019) [54]	Depressive symptoms and its associations with diabetes, HIV, hypertension and obesity a	1,037 PLWH40+; 4,022 HIV uninfected older adults ≥40 years	8-Item Center for Epidemiologic Studies Depression Scale (CESD)	NR	Not validated previously. The current version underwent intensive cultural adaptation	None of the chronic conditions was significantly associated with depressive symptoms in multivariable regressions.	Older age was positively associated with depression in both univariable and multivariable regression, whereas being married and employment was negatively associated.
Kaharuza, F. M., et al. (2006) [27]	Prevalence of depression	94 PLWH50+; 923 PLWH (18-50 years)	Modified version of the 20-Item CES-D	≥23	Internally consistent (alpha = 0.9)	58.5% among PLWH50+; 14.8% in PLWH (18-50 years)	Older age (>50 years) was positively associated with depressive symptoms; AOR 1.93 (95% CI: 1.09, 3.42).
Kalomo, E. N., et al. (2020). [48]	Association between HIV stigma, resilience, and depressive symptoms	147 PLWH50+	The 8-item geriatric depression scale	≥3 symptoms	Cronbach alpha of 0.73 in the current study	46.1% of the participants had suggestive depression.	There was a positive association between HIV stigma (negative self-perceptions) and depression and a negative relationship between resilience and depression. Also, being female and higher education were protective factors and disclosure of HIV status was a risk factor
Kellett-Wright, J., et al. (2020). [49]	Prevalence of major depressive symptoms	235 PLWH50+	DSM-IV	NR	NR	16.6% had major depression	NR
Kinyanda, E., et al. (2016) [55]	Prevalence of Major depressive disorder	244 PLWH50+; 224 HIV affected but uninfected older adults ≥50 years	MINI Plus	NR	NR	11.9% among PLWH50+; 6.3% in HIV affected but uninfected older adults ≥50 years	MDD was significantly associated with declining SES, increasing disability scores, decreasing mean grip strength, reported back pain, and not having hypertension.
Manne-Goehler, J., et al. (2019) [28]	Prevalence of depression	154 PLWH40+; 142 HIV uninfected older adults ≥40 years	15-Item Hopkins Checklist (HSCL)	Mean score of ≥1.75	Previously validated in the same population	21.4% among PLWH40+; 33.8% in uninfected older adults ≥40 years	In multivariable regression models, PLWH on ART and those falling in the highest wealth quartile had a lower prevalence of probable depression. In comparison, women had a significantly higher prevalence of depression than men. Education and age were not significantly associated with depression.
Motumma, A., et al. (2019). [50]	Prevalence of common mental disorders	122 PLWH40+; 298 HIV infected young adults (18-40) years	Self-Reporting Questionnaire (SRQ-20)	A score of ≥7 of SRQ-20 items	Previously validated in Ethiopia, with high sensitivity (85.7%) and specificity (75.6%).	23.0% among PLWH40+; and 30.2% among PLWH <40 years.	Correlates not aggregated by age

For peer review only - http://bmjopen.bmj.com/site/about/guidelines.xhtml

Author (Year)	Outcome of interest	Sample size	Assessment tool used	Cut-off score	Information on local tool validation	Prevalence estimates/other results	Correlates reported
Moucheraud, C., et al. (2020). [51]	Mild and major depressive symptoms	74 PLWH50+ and 60 young PLWH [30-49] years.	Center for Epidemiologic Studies Depression Scale (CES-D)	Mild/major depression symptoms = ≥16 CESD scores; Major depression symptoms = ≥27 CES-D scores	NR	Symptoms of mild/major depression: 32.4% among PLWH50+ versus 56.7% in the young PLWH.<br><br>Major depression symptoms: 10.8% versus 26.7% among young PLWH.	Older adults reported less few depressive symptoms (mild depression: aOR 0.23 p = 0.002; major depression: aOR 0.16, p = 0.004); in gender-stratified models, these relationships were significant only for females
Mugisha, J. O., et al. (2016) [56]	Prevalence of major depressive disorder	244 PLWH50+; 227 HIV affected but uninfected older adults ≥50 years	MINI	NR	Locally adapted	9.5% among PLWH50+; 5.8% in the uninfected older adults ≥50 years	NR
Musinguzi, K., et al. (2018) [29]	Prevalence of major depressive disorder	21 PLWH50+; 180 PLWH (18-49 years)	MINI	NR	NR	14.3% among PLWH50+; 32.8% in PLWH (18-49 years)	Reported correlates not aggregated by age.
Negin, J., et al. (2012) [57]	Prevalence of depressive symptoms	142 PLWH50+; 2,722 HIV uninfected older adults ≥50 years	Validated symptom questions from standard instruments (World mental health survey & CIDI)	NR	Locally adapted	6.0% among PLWH50+; 4.8% in the uninfected older adults ≥50 years	NR
Nyirenda, M., et al. (2013) [58]	Prevalence and correlates of major depressive episode	203 PLWH50+; 219 HIV uninfected older adults ≥50 years	Composite International Diagnostic Interview (CIDI)	ICD 10-DCR criteria	Locally adapted	14.8% among PLWH50+; 30.1 % in the uninfected older adults ≥50 years	Being female (aOR 3.04, 95% CI 1.73–5.36), receiving a government grant (aOR 0.34, 95% CI 0.15–0.75), urban residency (aOR 1.86, 95% CI 1.16–2.96) and adult caregiving (aOR 2.37, 95% CI 1.37–4.12) were significantly associated with any depressive episode
Olley, B. O., et al. (2017) [31]	Prevalence of depressive symptoms	44 PLWH50+; 458 PLWH (18-50 years)	20-item CES-D.	≥15	Cronbach's alpha = 0.79. Item-total correlation coefficient ranged between 0.65 and 0.87	20.5% among PLWH50+; 29.7% in PLWH (18-50 years)	Not aggregated by age
Rohr, J. K., et al. (2020). [59]	Prevalence of depressive symptoms	1048 PLWH40+; 3512 HIV uninfected older adults ≥40 years	The 8-item Center for Epidemiologic Studies—Depression Scale	≥4 symptoms	NR	8.0% among PLWH40+ versus 10.0% among HIV uninfected older adults ≥40 years.	NR

For peer review only - http://bmjopen.bmj.com/site/about/guidelines.xhtml

Author (Year)	Outcome of interest	Sample size	Assessment tool used	Cut-off score	Information on local tool validation	Prevalence estimates/other results	Correlates reported
Shumba, C., et al. (2013) [32]	Prevalence of depressive symptoms	91 PLWH50+; 584 PLWH (19-49 years)	Locally adapted 5-item symptom questionnaire	≥1	Cronbach's alpha of 0.87	53.9% among PLWH50+; 60.1% in PLWH (19-49 years)	NR
Tesfaw, G., et al. (2016) [34]	Prevalence of depressive symptoms	59 PLWH50+; 358 PLWH (19-49 years)	Seven items - depression subscale of Hospital Anxiety and Depression Scale	≥8	Previously validated	39.0% among PLWH50+; 41.6% in PLWH (18-49 years)	Not aggregated by age
Torgersen, J., et al. (2019) [42]	Prevalence of depressive symptoms	125 PLWH50+; 789 PLWH (21-49 years)	9-Item Patient Health Questionnaire	≥10	NR	16.0% among PLWH50+; 15.0% in PLWH (21-49 years)	NR
Obimakinde, A. M., et al. (2020) [39]	Anxiety disorder	62 PLWH50+; 162 HIV uninfected older adults ≥50 years	Extracted from participants clinical notes	NA	NR	3.2% among PLWH50+; 3.2% in HIV uninfected older adults	NR
Olagunju, A. T., et al. (2012) [30]	Prevalence of anxiety disorders	24 PLWH50+; 276 PLWH (18-50 years)	Clinical Assessment in Neuropsychiatry (SCAN)	International Classification of Diseases, 10th edition (ICD-10) criteria	NR	20.8% among PLWH50+; 21.7% in PLWH (18-50 years)	Not aggregated by age

Notes: ART – *Antiretroviral treatment*; **AOR –** *Adjusted odds ratio*; **CES-D –** *Center for Epidemiologic Studies Depression Scale*; **CI –** *Confidence interval*; **CIDI –** *Composite International Diagnostic Interview*; **DSM-IV TR –** *Diagnostic and Statistical Manual of Mental Disorders, fourth edition, text revision;* **HSCL –** *Hopkins Symptoms Checklist*; **ICD-10-DCR –** *Diagnostic Criteria for Research accompanying the International Classification of Diseases, Tenth Revision*; **MDD –** *Major depressive* disorder; **MINI –** *Mini International Neuropsychiatric Interview*; **NR –** *Not reported*; **PLWH –** *People living with HIV*; **PLWH50+ –** *People living with HIV ≥50 years old*; **SCAN –** *Clinical Assessment in Neuropsychiatry*

For peer review only - http://bmjopen.bmj.com/site/about/guidelines.xhtml

Table 3: Summary of prevalence estimates and correlates for cognitive impairment/neurological impairment

Author (Year)	Outcome of interest	Sample size	Assessment tool used	Cut-off score	Information on local tool validation	Prevalence estimates/other results	Correlates reported
Asiimwe, S. B., et al. (2020). [61]	Cognitive differences associated with HIV serostatus and ART use	1048 PLWH40+; 3512 HIV uninfected older adults ≥40 years	A battery of conventional instruments and the Oxford Cognitive Screen-Plus (OCS-Plus)	Not applicable. Summarised mean cognitive scores between participants with and without HIV on the two measures.	Previously validated in the study setting	PLWH scored on average 0.06 (95% CI 0.01–0.12) standard deviation (SD) units higher on the conventional cognitive function measure and 0.02 (95% CI − 0.07 to 0.04) SD units lower on the OCS-Plus measure than HIV-negative participants.	ART use was non-significantly associated with better cognitive outcomes among PLWH in both instruments.
Atashili, J., et al. (2013) [21]	Positive dementia screen	400 HIV infected adults (20-53 years old)	International HIV Dementia Scale (IHDS)	≤10 on IHDS	Not validated	The odds of having a positive dementia screen increased progressively with increasing participants' age, with the oldest group (≥50 years) having the greatest odds.	Not aggregated by age
Cassimjee, N. and P. K. Motswai (2017) [22]	Differences in neuropsychological functioning between PLWH and their uninfected peers	33 PLWH50+; 17 HIV uninfected older adults [mean age 52.2]	Dementia Rating Scale-2, the Stroop Colour and Word Test, the Symbol Digits Test and the D-KEFS Trail Making Test.	N/A	NR	PLWH, in comparison to the uninfected group, had significantly poorer performance profiles in global cognitive functioning, memory, executive functioning, psychomotor functioning and processing speed.	Not reported
Eaton, P., et al. (2020) [25]	Prevalence and risk factors for symptomatic HAND	253 PLWH50+	Comprehensive NP battery	Frascati criteria for classifying HAND	NR	Symptomatic HAND (HAD and MND) was available in 21.7%. HAD was present in 9 (3.6%).	Participants were at higher risk of having symptomatic HAND if they lived alone [OR = 2.6], were illiterate (OR= 3.2) or older at the time of HIV diagnosis (OR = 1.1). HIV-specific factors were not related to symptomatic HAND. Vascular risk factors, such as smoking status, were not associated with symptomatic HAND.
Joska, J. A., et al. (2019) [60]	Point prevalence of dementia and its associations	55 PLWH50+; 1,095 HIV uninfected older adults ≥50 years	Brief Community Screening Instrument for Dementia	NR	Previously validated	18.2% among PLWH50+; 10.7 % in the uninfected older adults ≥50 years	Dementia status was associated with HIV infection (OR= 2.2, CI=1–4.5), older age (OR =1.0, CI=1.0–1.1) and f depressive symptoms (OR 2.7, CI=1.8–3.9). The effects of education and marital status were not significant.

For peer review only - http://bmjopen.bmj.com/site/about/guidelines.xhtml

Author (Year)	Outcome of interest	Sample size	Assessment tool used	Cut-off score	Information on local tool validation	Prevalence estimates/other results	Correlates reported
Kellett-Wright, J., et al. (2020). [49]	Prevalence of HAND	235 PLWH50+	International HIV Dementia Scale (IHDS) and IDEA (Six Item Dementia Screen)	HAND diagnosis by 2007 AAN criteria was based on detailed consensus panel discussion	Both tools had limited diagnostic accuracy for HAND (area under the receiver operating characteristic curve 0.639–0.667 IHDS, 0.647–0.713 IDEA	Prevalence of HAND was 47.0%.<br>25.3% met the criteria for ANI, 18.2% MND, and 3.6% HAD.	Not reported
Kobayashi, L. C., et al. (2019) [53]	Cognitive impairment	1,048 PLWH40+;<br>4,011 HIV uninfected older adults ≥40 years	Brief cognitive battery (orientation, immediate & delayed recall).	≤ 1.5 SDs below the mean composite time orientation and memory score.	Locally adapted	4.0% among PLWH40+;<br>9.0% in the uninfected older adults ≥40 years	The independent predictors of lower cognitive score were being older, female, unmarried, not working, having a low formal education, low socioeconomic status, and a history of cardiovascular conditions.
Moucheraud, C., et al. (2020). [51]	Mean Cognitive function scores	74 PLWH50+ and 60 young PLWH (30-49) years	Brief cognitive battery (MMSE)	NR	NR	Mean cognitive scores:<br>16.0 among PLWH50+ versus 17.4 among young PLWH.	NR
Mugendi, A., et al. (2019) [38]	HIV-associated neurocognitive disorder (HAND)	64 PLWH50+;<br>281 PLWH (20-49 years)	Montreal Cognitive Assessment (MoCA) and IHDS	Combined scores of the 2 tools: a score of ≤26 on MOCA and ≤10 on the IHDS.	NR	Among PLWH50+: 79.0% ANI, 16.0% MND and 3.2% HAD.<br>Among PLWH (20-49 years): 75.7% ANI, 10.7% MND and 1.5% HAD.	Reported correlates not aggregated by age.
Ssonko, M., et al. (2018) [33]	Cognitive impairment	411 PLWH50+	Folstein's Mini-Mental State Examination	NR	NR	Mild cognitive impairment was associated with falls in the previous 12 months.	NR
Tsegaw, M., et al. (2017) [35]	Prevalence of HAND	67 PLWH56+;<br>526 PLWH (18-55 years)	International HIV Dementia Scale	≤9.5	NR	61.2% among PLWH56+; And 41.8% in PLWH (18-55 years)	Reported correlates not aggregated by age.
Obimakinde, A. M., et al. (2020) [39]	HIV associated peripheral neuropathy	62 PLWH50+;<br>162 HIV uninfected older adults ≥50 years	Extracted from participants medical notes	Clinical notes	NR	19.0% in PLWH50+	NR
Oumar, G., et al. (2020) [40]	Neurocognitive disorders	102 PLWH50+	Mini Mental State Examination	Frascati criteria for classifying HAND	NR	23.5% had neurocognitive disorders,	In a multivariate analysis, age (OR=4.6) and educational status (OR=2.6) were associated with neurocognitive disorders.

Notes: ANI – *Asymptomatic Neurocognitive Impairment;* **ART –** *Antiretroviral treatment*; **HAD** – *HIV-associated* dementia; **HAND** – *HIV-associated neurocognitive disorders*; **IDEA –** Item Dementia Screen**; IHDS** – *International HIV dementia scale*; **MMSE** – *Mini-mental Status Examination*; **MND** – *Mild neurocognitive* disorder; **NA** – *Not* applicable; **NP** – Neuropsychological; **NR** – *Not* reported; SD – *Standard* deviation; **OR –** *Odds ratio*; **PLWH –** *People living with HIV*; **PLWH40+ -** *People living with HIV ≥40* years; **PLWH50+ –** *People living with HIV ≥50 years old*

For peer review only - http://bmjopen.bmj.com/site/about/guidelines.xhtml

Table 4: Summary of prevalence estimates and correlates for frailty and handgrip strength

Author (Year)	Outcome of interest	Sample size	Assessment tool used	Cut-off score	Tool validation	Prevalence estimates/other results	Correlates reported
Bernard, C., et al. (2020). [45]	Levels of low Grip strength	333 PLWH50+	Calibrated Jamar hydraulic hand dynamometer	A performance less to the median obtained in each gender group (male/female)	Not reported	43.2% of women had low grip strength. 42.5% of men had low grip strength.	NR
Bristow, C., et al. (2021). [47]	Prevalence of Frailty and associated factors	145 PLWH50+	Fried frailty phenotype (FFP), Clinical Frailty Scale (CFS) and B-FIT 2).	**FFP:** A score of 1–2 was determined to be pre-frail, and a score of ≥ 3 was determined to be frail; **The B-FIT** (A score of 8/20); **CFS**, (CFS score 5–9)	BFIT and FFP previously validated in Tanzania.	2.8% (95% CI 0.09–5.4%) when **using the FFP.** 0.68% (CI − 0.66–2.04%) (when using **the CFS or B-FIT 2).**	FFP frailty was associated with female gender (p = 0.006), marital status (p = 0.007) and age (p = 0.038). Weight loss was the most common FFP domain failure.
Edwards, A., et al. (2020). [62]	Prevalence of frailty	292 PLWH50+ and 322 HIV uninfected adults (≥50) years.	Fried Frailty Phenotype (FFP)	Presence of ≥3 of the 5 FFP phenotypes	Adapted in the local population	Frailty was similar for HIV negative and PWH (17.7% vs 14.7%, p = 0.72); as was pre-frailty status [66.5% vs 63.4%].	The prevalence of frailty increased with age. There was no association between any of the inflammatory biomarkers and frailty and pre-frailty.
Filteau, S., et al. (2017) [18]	Change in mean handgrip strength	PLWH50+: 148 at baseline; 72 at 12wks, and 50 at 2-3 years ART	Digital handgrip dynamometer	NA	NR	Mean grip strength change 18.7 kgs at bassline, 20.4 kgs at 12 weeks and 23.1 kgs at 2-3 years.	Reported correlates not aggregated by age.
Mugisha, J., et al. (2013) [63]	Mean handgrip strength	198 PLWH50+; 311 HIV uninfected adults ≥50 years	Digital handgrip dynamometer	NA	NR	25.7 among PLWH50+; and 22.1 among the uninfected older adults ≥50 years.	NR
Negin, J., et al. (2012) [57]	Mean handgrip strength	142 PLWH50+; 2,722 HIV uninfected older adults ≥50 years	Digital handgrip dynamometer	NA	Locally validated	Grip strength in PLWH50+ (33.5 kg) was weaker than among HIV-uninfected individuals (38.3 kg).	When controlling for age and sex among those aged 50 years and older, HIV status was significantly associated with weaker grip strength.
Scholten, F., et al. (2011) [64]	Mean handgrip strength	189 PLWH50+; 2017 HIV affected 50+; and 104 HIV uninfected older adults ≥50 years	Digital handgrip dynamometer	NA	Locally adapted	25.8 among PLWH50+; and 25.3 among HIV affected 50+ and 25.2 among the uninfected older adults ≥50 years.	Women scored poorly than men. Married respondents had significantly better scores than widowed and divorced respondents. Respondents with at least secondary education had significantly better scores.
Ssonko, M., et al. (2018) [33]	Frailty	411 PLWH50+	Self-report 40 -item frailty index questionnaire	NR	NR	Frailty index scores of 5 to 6 (PR = 10.6, 95% C.I. 1.4, 78, p = 0.02), and 7 or more (PR = 17.4, 95% C.I. 2.4, 126.5, p = 0.005) were associated with polypharmacy	Increasing Frailty score was associated with increasing levels of polypharmacy.

Notes: ART – *Antiretroviral treatment*; **B-FIT 2 –** *Brief Frailty Instrument of Tanzania*; **CFS –** *Clinical Frailty Scale*; **FFP –** *Fried Frailty Phenotype*; **NA –** *Not* applicable; **NP –** Neuropsychological; **NR –** *Not* reported; **PLWH –** *People living with HIV*; **PLWH50+ –** *People living with HIV ≥50 years old*; **PR –** *Prevalence ratio*

For peer review only - http://bmjopen.bmj.com/site/about/guidelines.xhtml

Table 5: Summary of results for health-related quality of life

Author (Year)	Outcome of interest	Sample size	Assessment tool used	Cut-off score	Information on local tool validation	Prevalence estimates/other results	Correlates reported
Harding, R., et al. (2014) [26]	Self-reported mental and Physical aspect of Quality of Life	1,337 adults aged 18-70 years (*number of PLWH50+ NR)*	Medical Outcome Scale - HIV (MOS-HIV)	NA	Previously validated in other SSA setting	**Mean Mental Health score:** 46.4 among PLWH50+;<br>46.2 in PLWH (18-49 years)<br>**Physical health score**:<br>42.9 among PLWH50+; and<br>44.8 in PLWH (18-49 years)	Reported correlates not aggregated by age.
Maniragaba, F., et al. (2018) [66]	Quality of life	28 PLWH50+;<br>884 HIV uninfected older adults ≥60 years	An adapted version of the WHOQOL-OLD	An index (good, fair and poor quality of life)<br>.	NR	PLWH had poor quality of life (OR=0.45; CI=0.22-0.93) compared to those who were HIV uninfected.	Not aggregated by HIV status
Mugisha, J., et al. (2013) [63]	Self-reported Mean quality of life	198 PLWH50+;<br>311 HIV uninfected older adults ≥50 years	8-Item WHOQoL	NA	NR	57.2 among PLWH50+; and<br>45.3 in the uninfected older adults ≥50 years	NR
Nyirenda, M., et al. (2012) [65]	Self-reported Median Quality of life	203 PLWH50+;<br>219 HIV uninfected older adults ≥50 years	8-Item WHOQoL	NA	Locally adapted	62.5 among PLWH50+; and<br>59.4 in the uninfected older adults ≥50 years. PLWH50+ had better QoL than uninfected older adults.	Being female and having been previously married was associated with lower odds of good quality of life in PLWH50+
Nyirenda, M., et al. (2013) [70]	Self-reported quality of life	400 PLWH50+;<br>732 HIV uninfected older adults ≥50 years	8-Item WHOQoL	NA	Locally adapted and validated	Older people in South Africa were more likely to have a better quality of life (aOR 2.15, 95% CI 1.60–2.90) than those in Uganda.	NR
Obimakinde, A. M., et al. (2020) [39]	Self-reported mean quality of life	62 PLWH50+;<br>124 HIV uninfected older adults ≥50 years	8-item WHOQoL	NA	NR	Mean (SD):82.7 (8.9) among PLWH50+;<br>86.2 (6.5) in the uninfected adults ≥50 years.	NR
Parcesepe, A. M., et al. (2020) [41]	Self-reported Mean Quality of life	82 PLWH50+;<br>824 PLWH (18-49 years)	HIV/AIDS-Targeted Quality of Life Scale	NA	Previously validated in other SSA settings	68 among PLWH50+, and<br>72.4 among PLWH (18-49 years)	Reported correlates not aggregated by age.
Yaya, I., et al. (2019). [52]	Self-reported levels of quality of life	154 PLWH50+;<br>726 PLWH (15-49 years)	31- Item WHOQOL-HIV BREF	A score higher than 77.3 was considered good global quality of life.	NR	72.7% of PLWH50+ had a good global quality of life compared to 77.3% in the younger PLWH.	Correlates not aggregated by age

Notes: ART – *Antiretroviral treatment*; **AOR –** *Adjusted odds ratio*; **OR –** *Odds ratio*; **CI –** *Confidence interval*; **NA –** *Not* applicable; **NR –** *Not reported*; **PLWH –** *People living with HIV*; **PLWH50+ –** *People living with HIV ≥50 years old*; **QoL** – *Quality of* life; **SSA** – *sub-Saharan* Africa; **SD –** *Standard deviation*; **WHOQOL** – *World Health Organization Quality of life measure*

For peer review only

Figures: A Systematic Review of Health and Wellbeing in older adults living with HIV in sub-Saharan Africa

Figure 1: Identification of eligible studies

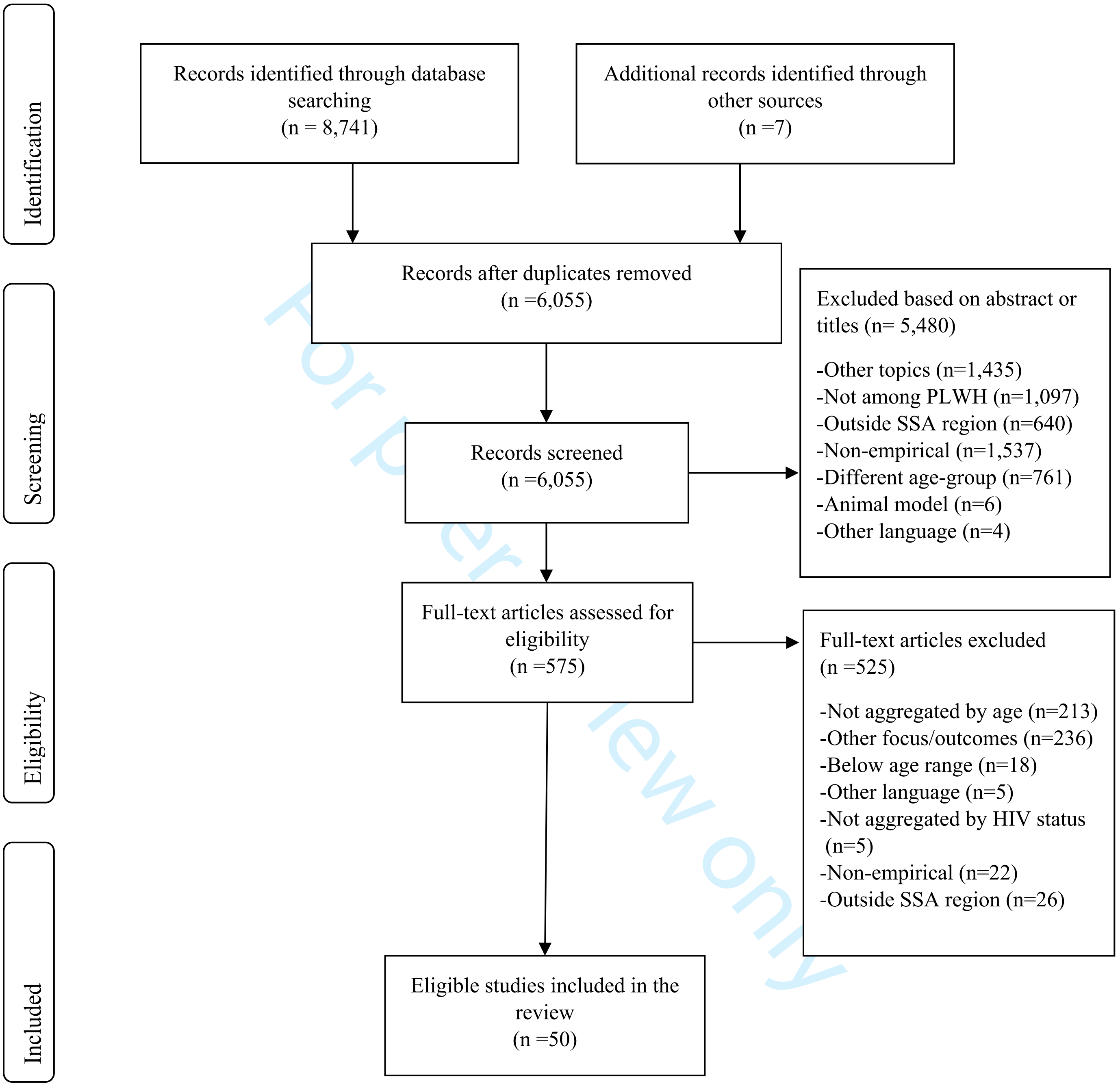

For peer review only

Figures: A Systematic Review of Health and Wellbeing in older adults living with HIV in sub-Saharan Africa

Figure 2: Geographical distribution of the included studies within SSA

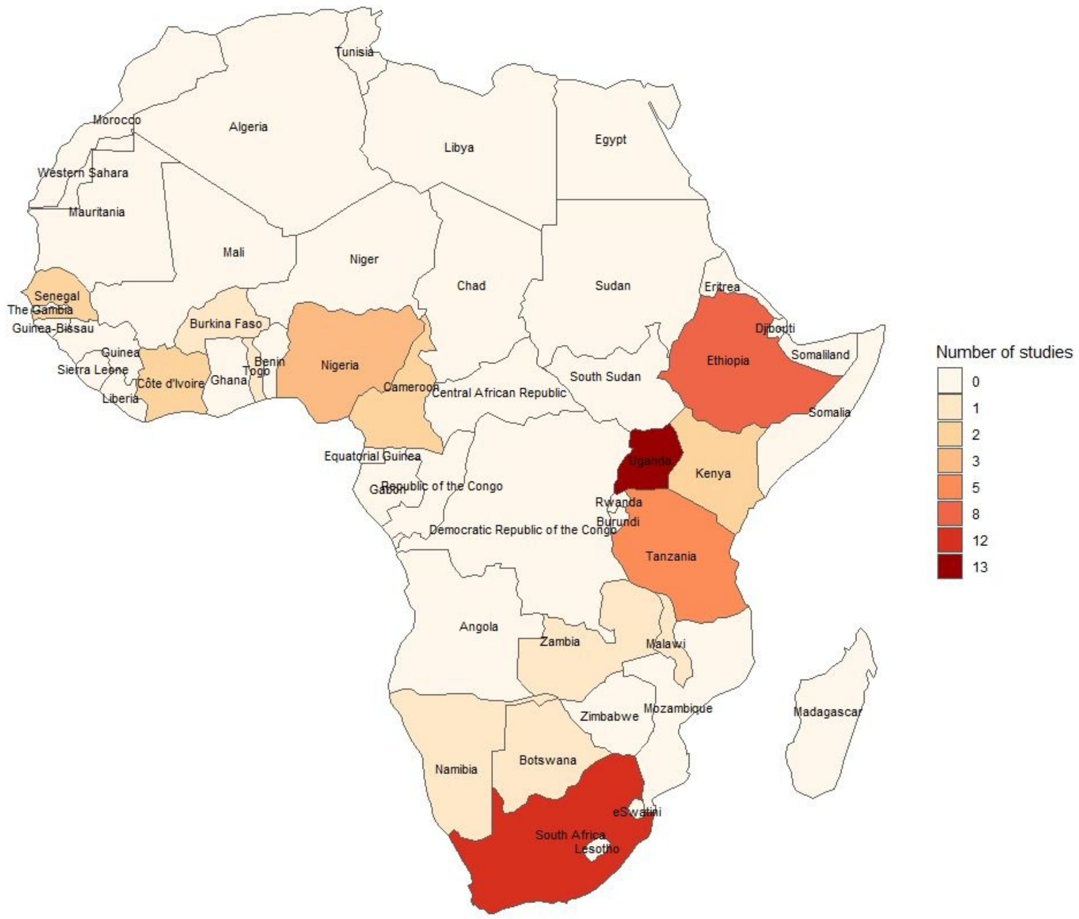

Supplementary Files – A Systematic Review of Health and Wellbeing in older adults living with HIV in sub-Saharan Africa

Supplementary table 1. Modified Newcastle-Ottawa Risk of Bias Assessment

Author	Representativeness of the sample	Sample size	Comparability between respondents and non-respondents	Outcome ascertainment	Quality of Statistics reporting	Total quality score
**Abadiga, M. (2019).**	**1**	**0**	**1**	**1**	**1**	**4**
Akena, D., et al. (2012).	1	0	0	0	1	2
Asangbeh, S. L., et al. (2016)	0	0	0	1	1	2
Asiimwe, S. B., et al. (2020).	1	1	1	1	1	5
Asrat, B., et al. (2020).	1	0	1	1	1	4
Atashili, J., et al. (2013)	0	0	1	0	1	2
Berhe, H. and A. Bayray (2013)	0	0	0	0	1	1
Bernard, C., et al. (2020).	1	1	0	0	1	3
Bernard, C., et al. (2020).	1	1	0	0	1	3
Bristow, C., et al. (2021).	1	1	1	1	1	5
Cassimjee, N. and P. K. Motswai (2017)	0	0	0	1	1	2
Cholera, R., et al. (2017).	1	0	1	1	1	4
Duko, B., et al. (2018).	1	0	0	1	1	3
Eaton, P., et al. (2020)	1	1	0	1	1	4
Edwards, A., et al. (2020).	1	1	1	0	1	4
Eshetu, D. A., et al. (2015).	0	0	0	0	1	1
Filteau, S., et al. (2017).	1	1	0	1	1	4
Geldsetzer, P., et al. (2019).	1	1	1	0	1	4
Harding, R., et al. (2014).	0	0	0	1	1	2
Joska, J. A., et al. (2019).	1	1	0	0	1	3
Kaharuza, F. M., et al. (2006)	1	0	0	1	1	3
Kalomo, E. N., et al. (2020)	0	1	0	0	1	2
Kellett-Wright, J., et al. (2020)	1	1	0	1	1	4
Kinyanda, E., et al. (2016)	1	1	0	1	1	4
Kobayashi, L. C., et al. (2019)	1	1	1	1	1	5
Maniragaba, F., et al. (2018)	1	0	0	0	1	2
Manne-Goehler, J., et al. (2019).	0	1	0	1	1	3
Motumma, A., et al. (2019).	1	1	1	1	1	5
Moucheraud, C., et al. (2020)	0	0	0	1	1	2
Mugendi, A., et al. (2019).	0	0	0	0	1	1
Mugisha, J., et al. (2013)	1	1	0	1	1	4
Mugisha, J. O., et al. (2016).	1	1	0	1	1	4
Musinguzi, K., et al. (2018).	1	0	0	0	1	2
Negin, J., et al. (2012)	1	1	0	1	1	4
Nyirenda, M., et al. (2012).	1	1	0	1	1	4
Nyirenda, M., et al. (2013).	1	1	0	1	1	4
Nyirenda, M., et al. (2013)	1	1	0	1	1	4
Obimakinde, A. M., et al. (2020).	0	0	1	0	1	2
Olagunju, A. T., et al. (2012).	1	0	0	1	1	3
Olley, B. O., et al. (2017).	0	0	0	1	1	2
Oumar, G., et al. (2020).	0	1	0	0	1	2
Parcesepe, A. M., et al. (2020).	0	0	0	1	1	2
Rohr, J. K., et al. (2020)	1	1	1	0	1	4
Scholten, F., et al. (2011).	1	1	0	1	1	4
Shumba, C., et al. (2013).	1	0	0	1	1	3
Ssonko, M., et al. (2018).	1	1	0	0	1	3
Tesfaw, G., et al. (2016)	1	0	1	1	1	4
Torgersen, J., et al. (2019).	0	1	1	0	1	3
Tsegaw, M., et al. (2017)	0	0	1	0	1	2
Yaya, I., et al. (2019).	1	1	1	0	1	4

For peer review only

Research Checklist– A Systematic Review of Health and Wellbeing in older adults living with HIV in sub-Saharan Africa

PRISMA 2020 Checklist

Section and Topic	Item #	Checklist item	Location where item is reported
**TITLE**			
Title	1	Identify the report as a systematic review.	Page 1
**ABSTRACT**			
Abstract	2	See the PRISMA 2020 for Abstracts checklist.	Page 2
**INTRODUCTION**			
Rationale	3	Describe the rationale for the review in the context of existing knowledge.	Page 3
Objectives	4	Provide an explicit statement of the objective(s) or question(s) the review addresses.	Page 3 and 4
**METHODS**			
Eligibility criteria	5	Specify the inclusion and exclusion criteria for the review and how studies were grouped for the syntheses.	Page 4
Information sources	6	Specify all databases, registers, websites, organisations, reference lists and other sources searched or consulted to identify studies. Specify the date when each source was last searched or consulted.	Page 4
Search strategy	7	Present the full search strategies for all databases, registers and websites, including any filters and limits used.	Page 4
Selection process	8	Specify the methods used to decide whether a study met the inclusion criteria of the review, including how many reviewers screened each record and each report retrieved, whether they worked independently, and if applicable, details of automation tools used in the process.	Page 4
Data collection process	9	Specify the methods used to collect data from reports, including how many reviewers collected data from each report, whether they worked independently, any processes for obtaining or confirming data from study investigators, and if applicable, details of automation tools used in the process.	Page 4
Data items	10a	List and define all outcomes for which data were sought. Specify whether all results that were compatible with each outcome domain in each study were sought (e.g. for all measures, time points, analyses), and if not, the methods used to decide which results to collect.	Page 4 and 5
	10b	List and define all other variables for which data were sought (e.g. participant and intervention characteristics, funding sources). Describe any assumptions made about any missing or unclear information.	N/A
Study risk of bias assessment	11	Specify the methods used to assess risk of bias in the included studies, including details of the tool(s) used, how many reviewers assessed each study and whether they worked independently, and if applicable, details of automation tools used in the process.	Page 4
Effect measures	12	Specify for each outcome the effect measure(s) (e.g. risk ratio, mean difference) used in the synthesis or presentation of results.	Page 4
Synthesis methods	13a	Describe the processes used to decide which studies were eligible for each synthesis (e.g. tabulating the study intervention characteristics and comparing against the planned groups for each synthesis (item #5)).	N/A
	13b	Describe any methods required to prepare the data for presentation or synthesis, such as handling of missing summary statistics, or data conversions.	N/A
	13c	Describe any methods used to tabulate or visually display results of individual studies and syntheses.	Page 4

Research Checklist– A Systematic Review of Health and Wellbeing in older adults living with HIV in sub-Saharan Africa

Section and Topic	Item #	Checklist item	Location where item is reported
	13d	Describe any methods used to synthesize results and provide a rationale for the choice(s). If meta-analysis was performed, describe the model(s), method(s) to identify the presence and extent of statistical heterogeneity, and software package(s) used.	Page 4 and 5
	13e	Describe any methods used to explore possible causes of heterogeneity among study results (e.g. subgroup analysis, meta-regression).	N/A
	13f	Describe any sensitivity analyses conducted to assess robustness of the synthesized results.	N/A
Reporting bias assessment	14	Describe any methods used to assess risk of bias due to missing results in a synthesis (arising from reporting biases).	N/A
Certainty assessment	15	Describe any methods used to assess certainty (or confidence) in the body of evidence for an outcome.	N/A
**RESULTS**			
Study selection	16a	Describe the results of the search and selection process, from the number of records identified in the search to the number of studies included in the review, ideally using a flow diagram.	Page 5
	16b	Cite studies that might appear to meet the inclusion criteria, but which were excluded, and explain why they were excluded.	N/A
Study characteristics	17	Cite each included study and present its characteristics.	Page 20 to 23
Risk of bias in studies	18	Present assessments of risk of bias for each included study.	Supplementary table 1
Results of individual studies	19	For all outcomes, present, for each study: (a) summary statistics for each group (where appropriate) and (b) an effect estimate and its precision (e.g. confidence/credible interval), ideally using structured tables or plots.	Pages 6 to 9; Pages 24 to 31
Results of syntheses	20a	For each synthesis, briefly summarise the characteristics and risk of bias among contributing studies.	Pages 6 to 9
	20b	Present results of all statistical syntheses conducted. If meta-analysis was done, present for each the summary estimate and its precision (e.g. confidence/credible interval) and measures of statistical heterogeneity. If comparing groups, describe the direction of the effect.	N/A
	20c	Present results of all investigations of possible causes of heterogeneity among study results.	N/A
	20d	Present results of all sensitivity analyses conducted to assess the robustness of the synthesized results.	N/A
Reporting biases	21	Present assessments of risk of bias due to missing results (arising from reporting biases) for each synthesis assessed.	N/A
Certainty of evidence	22	Present assessments of certainty (or confidence) in the body of evidence for each outcome assessed.	N/A
**DISCUSSION**			
Discussion	23a	Provide a general interpretation of the results in the context of other evidence.	Pages 11-13
	23b	Discuss any limitations of the evidence included in the review.	Page 13-14

For peer review only

Research Checklist– A Systematic Review of Health and Wellbeing in older adults living with HIV in sub-Saharan Africa

Section and Topic	Item #	Checklist item	Location where item is reported
	23c	Discuss any limitations of the review processes used.	Page 13-14
	23d	Discuss implications of the results for practice, policy, and future research.	Page 14
**OTHER INFORMATION**			
Registration and protocol	24a	Provide registration information for the review, including register name and registration number, or state that the review was not registered.	Page 4
	24b	Indicate where the review protocol can be accessed, or state that a protocol was not prepared.	Page 4
	24c	Describe and explain any amendments to information provided at registration or in the protocol.	N/A
Support	25	Describe sources of financial or non-financial support for the review, and the role of the funders or sponsors in the review.	Page 14
Competing interests	26	Declare any competing interests of review authors.	Page 15
Availability of data, code and other materials	27	Report which of the following are publicly available and where they can be found: template data collection forms; data extracted from included studies; data used for all analyses; analytic code; any other materials used in the review.	Page 15

For peer review only

Mental health and Wellbeing of older adults living with HIV in sub-Saharan Africa: a systematic review

Journal:	*BMJ Open*
Manuscript ID	bmjopen-2021-052810.R1
Article Type:	Original research
Date Submitted by the Author:	09-Aug-2021

Complete List of Authors:	Mwangala, Patrick; KEMRI-Wellcome Trust Research Programme, neurosciences; University of the Witwatersrand School of Public Health,<br>Mabrouk, Adam; KEMRI-Wellcome Trust Research Programme, Neurosciences<br>Wagner, Ryan; University of the Witwatersrand, MRC/Wits Rural Public Health and Health Transitions Research Unit (Agincourt), School of Public Health, Faculty of Health Sciences<br>Newton, Charles; Centre for Geographic Medicine Research Coast, Psychiatry, Oxford University; University of Oxford Department of Psychiatry<br>Abubakar, Amina A.; KEMRI-Wellcome Trust Research Programme, neurosciences; Institute for Human Development, Aga Khan University

**Primary Subject Heading**:	HIV/AIDS
Secondary Subject Heading:	Mental health, Geriatric medicine
Keywords:	HIV & AIDS < INFECTIOUS DISEASES, PSYCHIATRY, PUBLIC HEALTH

I, the Submitting Author has the right to grant and does grant on behalf of all authors of the Work (as defined in the below author licence), an exclusive licence and/or a non-exclusive licence for contributions from authors who are: i) UK Crown employees; ii) where BMJ has agreed a CC-BY licence shall apply, and/or iii) in accordance with the terms applicable for US Federal Government officers or employees acting as part of their official duties; on a worldwide, perpetual, irrevocable, royalty-free basis to BMJ Publishing Group Ltd ("BMJ") its licensees and where the relevant Journal is co-owned by BMJ to the co-owners of the Journal, to publish the Work in this journal and any other BMJ products and to exploit all rights, as set out in our licence.

The Submitting Author accepts and understands that any supply made under these terms is made by BMJ to the Submitting Author unless you are acting as an employee on behalf of your employer or a postgraduate student of an affiliated institution which is paying any applicable article publishing charge ("APC") for Open Access articles. Where the Submitting Author wishes to make the Work available on an Open Access basis (and intends to pay the relevant APC), the terms of reuse of such Open Access shall be governed by a Creative Commons licence – details of these licences and which Creative Commons licence will apply to this Work are set out in our licence referred to above.

Other than as permitted in any relevant BMJ Author's Self Archiving Policies, I confirm this Work has not been accepted for publication elsewhere, is not being considered for publication elsewhere and does not duplicate material already published. I confirm all authors consent to publication of this Work and authorise the granting of this licence.

For peer review only

Mental health and Wellbeing of older adults living with HIV in sub-Saharan Africa: a systematic review

*Patrick N Mwangala[1,2], Adam Mabrouk[1], Ryan G Wagner[3], Charles R Newton[1, 4,] Amina Abubakar[1, 4 5,6]

Author Affiliations:

[1]Centre for Geographic Medicine Research Coast, Kenya Medical Research Institute (KEMRI), P.O Box 230-80108, Kilifi, Kenya

[2]School of Public Health, University of the Witwatersrand, 27 St Andrews Road, Parktown 2193, South Africa

[3]MRC/Wits Rural Public Health and Health Transitions Research Unit (Agincourt), University of the Witwatersrand, Faculty of Health Sciences, Johannesburg, South Africa

[4]Department of Psychiatry, University of Oxford, Warneford Hospital, Warneford Ln, Oxford OX3 7JX, United Kingdom

[5]Department of Public Health, Pwani University, P.O. BOX 195-80108, Kilifi, Kenya

[6]Institute for Human Development, Aga Khan University, P.O. BOX 30270-00100, Nairobi, Kenya

*Corresponding author: Patrick N Mwangala; Email: pmwangala27@gmail.com; Tel: +254 708 759457

For peer review only

For peer review only - http://bmjopen.bmj.com/site/about/guidelines.xhtml

ABSTRACT

Objective: To summarise the empirical evidence on common mental disorders (CMDs), cognitive impairment, frailty and health-related quality of life (HRQoL) among people infected with human immunodeficiency virus (HIV) aged ≥50 years (PLWH50+) residing in sub-Saharan Africa (SSA). Specifically, we document the prevalence and correlates of these outcomes.

Design, data sources and eligibility criteria: The following online databases were systematically searched: PubMed, CINAHL, PsycINFO, Embase and Scopus up to Jan 2021. Research articles on depression, anxiety, cognitive function, frailty, and quality of life among PLWH50+ residing in SSA were included in the review.

Data extraction and synthesis: We extracted information, including study characteristics, and main findings. These were tabulated, and a narrative synthesis approach was adopted, given the substantial heterogeneity among included studies.

Results: A total of 50 studies from fifteen SSA countries met the inclusion criteria. About two-thirds of these studies emanated from Ethiopia, Uganda, and South Africa. Studies regarding depression predominated (*n=26),* followed by cognitive impairment (*n=13*). Overall, PLWH50+ exhibited varying prevalence of depression (6% to 59%), cognitive impairments (4% to 61%) and frailty (3% to 15%). The correlates of CMDs, cognitive impairment, frailty and HRQoL were rarely investigated, but those reported were sociodemographic variables, many of which were inconsistent.

Conclusions: This review documented an increasing number of published studies on HIV and aging from SSA. However, we find the current evidence on the mental and wellbeing outcomes in PLWH50+ inadequate to characterise the public health dimension of these impairments in SSA, because of heterogeneous findings, a limited number of well-designed studies and substantial methodological limitations in many of the available studies. Future work should have sufficiently large samples of PLWH50+, engage appropriate comparison groups, harmonise the measurement of these outcomes using a standardised methodology to generate more robust prevalence estimates and confirm predictors.

PROSPERO registration number: CRD42020145791

Strengths and Limitations of this study

- This is the first comprehensive review of common mental disorders, cognitive impairment, frailty and quality of life among older adults living with HIV in SSA, synthesising the current evidence regarding the prevalence and correlates of these outcomes.
- Our expanded search of five online databases and snowballing techniques minimised the risk of missing relevant studies.
- Meta-analysis was not conducted due to extensive heterogeneity across the studies in methods and presentation of results.
- We observed substantial methodological limitations in many of the included studies, e.g. small sample sizes, making it difficult to characterise the real public health dimension of these impairments in the region.

For peer review only

For peer review only - http://bmjopen.bmj.com/site/about/guidelines.xhtml

1.0 BACKGROUND

All societies globally are experiencing an aging population – some are in its early stages, and some are more advanced [1]. Likewise, the proportion of people living with HIV (PLWH) aged ≥50 years (considered as older adults within the HIV literature) is growing rapidly across the globe [2], demanding an increase in research, policy and practice to address the complex needs of this vulnerable group [3]. In 2016 there were around 6 million PLWH50+ globally, representing 16% of the entire adult HIV population [2]. This proportion was projected to reach 21% by the end of 2020, with Southern and Eastern Africa containing the vast majority of PLWH50+ [2]. The increase in older PLWH is mainly driven by two factors: effective antiretroviral therapy (ART) and new HIV infections among the elderly [4].

This rapidly growing segment of PLWH50+ yields new challenges. Several HIV and aging cohorts (mostly from high-income countries – HICs) are pointing out an elevated burden of physical conditions (e.g. cardiometabolic diseases), mental morbidities (e.g. depression, cognitive disorders), psychosocial challenges (e.g. stigma, loneliness), and geriatric syndromes (e.g. frailty) among PLWH ≥50 years (PLWH50+) compared to their uninfected counterparts [4-6]. Unfortunately, the mechanisms for the heightened risk of poor outcomes among PLWH50+ are not fully understood but span a host of disease-related factors, legacy of the early years of the HIV epidemic, and host vulnerability factors [4]. Frailty, common mental disorders (CMDs) such as depression and anxiety, and cognitive impairments are of particular concern in this population [4-6]. Frailty is an important emerging indicator of vulnerability that is increasingly being evaluated among individuals aging with HIV [7]. It describes a state of physiologic vulnerability that, in the presence of internal or external stressors, puts a person at an elevated risk of adverse clinical outcomes, such as hospitalisation, functional disability, and mortality [8, 9]. Overall, mental morbidities and frailty are understudied in the literature despite their elevated burden and potential adverse impacts on HIV treatment and health-related quality of life (HRQoL).

Despite the apparent need to sharpen the focus on the needs of the growing cohort of PLWH50+, this population is being neglected in HIV care and prevention efforts, especially in low- and middle-income settings of sub-Saharan Africa (SSA). In SSA, this concern is not just based on the close association between aging and physical, cognitive, mental impairments, but also on the recognition that older adults are not considered to be a priority for policy issues. They are marginalised and increasingly vulnerable to greater poverty [10, 11]. Thus, the burden and determinants of physical and mental impairments in these adults in SSA, the region most affected by HIV globally, are largely undocumented [12].

The course and implication of physical and mental morbidities among PLWH50+ are well described in HICs, but it is an emergent research area in SSA. However, since 2010, the SSA region has witnessed a rise in interest in HIV and aging outcomes, evidenced by the establishment of cohort studies of older people in different parts of Africa [13-15]. This is encouraging since data from HICs may not be readily generalized to SSA where differences in, e.g. genetics, social-environmental milieu, formal and informal support systems will almost certainly alter the risk profile and morbidity characteristics among PLWH50+. Therefore, the current review aims to summarise the existing empirical evidence on common mental disorders (CMDs), cognitive impairment, frailty, and health-related quality of life (HRQoL) among PLWH50+ in SSA by addressing the following specific objectives.

For peer review only - http://bmjopen.bmj.com/site/about/guidelines.xhtml

a) Establish the prevalence of CMDs, cognitive impairment and frailty among PLWH50+ in SSA.

b) Identify the correlates of CMDs, cognitive impairment, frailty and HRQoL among PLWH50+ in SSA.

2.0 METHODS

We followed the Preferred Reporting Items for Systematic Reviews and Meta-analyses (PRISMA) guidelines to report the study [16]. The review protocol was prospectively registered in PROSPERO (Registration number: CRD42020145791).

2.1. Search strategy. Five online databases (PubMed, CINAHL, PsycINFO, Embase, and Scopus) were searched for publications. The last search was conducted on Jan 4th, 2021. The following search terms were used: (HIV OR HIV-1 OR HIV/AIDS OR HIV infections) AND (adult OR older adult OR older people OR older individual OR elderly) AND (Africa OR sub-Saharan Africa OR Africa South of the Sahara) AND (cognitive impairment OR neurocognitive impairment OR neurological complication OR HIV-associated neurocognitive disorder OR common mental disorder OR depression OR depressive symptoms OR depressive disorder OR anxiety OR anxiety disorder OR quality of life OR health-related quality of life OR grip strength OR hand strength OR frailty). We also searched through the reference lists of included articles to access additional articles.

Studies were considered eligible if they: (i) involved PLWH50+ or had participants whose mean/median age was ≥ 50 years or aggregated their outcomes of interest by age (including the ≥ 50 age category); (ii) were conducted within SSA; (iii) were empirical and published in a peer-reviewed journal; (iv) reported any of our outcomes of interest (CMDs, cognitive impairment, frailty or HRQoL). We excluded studies that did not aggregate the outcomes of interest by HIV status. Two of the authors (PNM and AM) independently screened the titles, abstracts and full articles for eligibility and reached consensus.

2.2. Data extraction and quality assessment. The two authors (PNM and AM) independently extracted the following study characteristics using a similarly designed extraction sheet: (i) first author; (ii) country and the year of publication; (iii) sample description; (iv) how the outcome of interest was measured; (v) results reported; (vi) key findings on the outcome of interest. For studies exclusively carried out among PLWH50+, we computed or extracted the reported percentages of the relevant outcomes. Similarly, for studies with middle-aged participants but whose mean/median age was ≥50 years, we also computed or extracted the reported percentages for the relevant outcomes for the whole sample. For the studies with middle-aged participants (but whose mean/median age was not at least 50 years), we computed percentages for participants who fell within the ≥ 50 category, which was usually provided in the papers. In some studies, it was impossible to calculate these percentages. Hence, the occurrence of a specific outcome was reported in the original effect measure, e.g. odds ratio, median or mean. Any disagreement between the reviewers during data abstraction was also resolved by consensus.

We evaluated the quality of included studies using the modified version of the Newcastle-Ottawa Scale (NOS) [17]. The scale uses five parameters [*sample size, sample representativeness, comparability between respondents and non-respondents, ascertainment of the outcome of interest, and quality of descriptive statistics reporting*] to obtain a total

For peer review only - http://bmjopen.bmj.com/site/about/guidelines.xhtml

score ranging from 0 – 5. A score ≥3 is considered a low risk of bias, while a score of <3 is regarded as a high risk of bias.

2.3. Data Synthesis and Analysis. A narrative synthesis approach was used to compare studies and describe the state of evidence on all reported outcomes; by describing the effect estimates, for instance, proportions, means with standard deviations, and median with their interquartile ranges. Where applicable, ranges were used to summarise the outcomes of interest reported. A meta-analysis could not be done due to a limited number of studies and substantial heterogeneity among studies.

2.4. Patient and Public Involvement Statement. The accompanying manuscript is part of broader doctoral research work for the first author. During the conceptual phase of this work, community engagement through meetings with different stakeholders was carried out to contextualise the issues of aging and HIV at the coast of Kenya (e.g. in terms of priorities and preferences). These stakeholders included the Kilifi and Mombasa counties' departments of Health, sub-county health management teams, and hospital management teams of various health facilities at the study setting. The research team received and incorporated useful feedback from these meetings towards the design and implementation of the project. It also led to further elaborations regarding ongoing community engagement during and after the study, including disseminating any work arising from this project using these forums and others such as publications, hospital departmental meetings, patient groups and conferences as an essential way to discuss the implications of the findings. For this systematic review, patients were not involved in the conduct of the study. However, the results will be disseminated to the relevant patient groups within the HIV clinics through feedback meetings.

3.0 RESULTS

3.1. Characteristics of included studies

We identified 8,741 articles from the 5 online databases and an additional 7 citations from snowballing. Of these, 2,693 were duplicates. Hence, we screened 6,055 titles and abstracts for initial eligibility, out of which 575 articles were identified. Full articles were obtained for these citations, of which 50 met the eligibility criteria (Figure 1). The eligible studies were conducted between 2006 and 2021 among 15 SSA countries of Uganda (n=13), South Africa (n=12), Ethiopia (n=8), Tanzania (n=5), Nigeria (n=3), Cameroon (n=2), Côte d'Ivoire (n=2), Senegal (n=2), Kenya (n=2), Botswana (n=1), Zambia (n=1), Malawi (n=1), Namibia (n=1), Togo (n=1) and Burkina Faso (n=1). However, some of these studies were conducted in multiple countries. Figure 2 shows the geographical distribution of these studies across the SSA region. Virtually all the included studies were cross-sectional, except one that presented data from a randomised trial [18]. Eighteen studies (36%) analysed baseline or follow up data of different cohort studies.

About two-thirds of the included studies *(n=35)* recruited their HIV infected samples from clinical settings [18-52]. The rest obtained their samples from the general population through household surveys and community samples. Additionally, many of the studies included samples of PLWH50+ lower than 100 (*n=22*). Overall, the samples of PLWH50+ per study ranged from 19 to 1,048 [36, 53], while those for HIV uninfected older adults ranged from 17 to 4,022 [22, 54].

For peer review only - http://bmjopen.bmj.com/site/about/guidelines.xhtml

Common HIV disease markers such as the duration of HIV infection, current/nadir CD4+ T cell count, duration of ART use, viral suppression level among PLWH50+ were reported in a few studies. However, there was extensive heterogeneity in how the information was reported and the level of detail given. In 42% of the included studies, all PLWH50+ were on ART. Six other studies included only ART naïve PLWH50+. Among ART users, viral suppression was reported in 5 studies where it was documented to range from 73% - 90%. Nadir CD4 + T cell count among PLWH50+ was reported in 4 studies where most of the participants (about 60%) had a count lower than 200. The duration of HIV infection among PLWH50+ ranged from 3.5 to 12 years (among the few studies that reported this variable).

Only eight studies examined the health outcomes of interest using an exclusively HIV infected older sample [25, 33, 40, 45-49]. Eighteen other studies used HIV uninfected older adults as comparison groups, while the rest included HIV infected young adults (the ages varied extensively).

Insert Figure 1 – ***Identification of eligible studies*** – here

Insert Figure 2 – ***Geographical distribution of the included studies within SSA*** – here

3.2. Common mental disorders.

Depression was reported in 25 studies, while anxiety was reported in 2 studies. Notably, depression was assessed using multiple scales with diverse scoring systems. Six of the studies used the 9-item Patient Health Questionnaire – PHQ-9, while a similar number used the Center for Epidemiological Studies Depression scale – CES-D. Five other studies used the MINI International Neuropsychiatric Interview – MINI, while the rest utilized a host of different scales. Nine of the depression studies [19, 29, 44, 49, 51, 55-58] reported major depressive disorders (MDD), while the remaining sixteen studies reported the prevalence of depressive symptoms. To synthesise and report the available evidence, depression (whenever used in this review) captures both the depressive symptoms (derived from brief screening measures) and Major Depressive Disorder (based on structured diagnostic interviews, e.g. the MINI by specialised professionals or trained non-specialised workers). More information on this outcome is presented in the supplementary table 3.

Prevalence of depression and anxiety

Overall, the prevalence of depression ranged from 6% to 59%. We found no clear pattern of overreporting or underreporting of depression prevalence in the included studies, considering the different cut-offs used. For instance, in studies using the PHQ-9, the prevalence of depression ranged from 47% to 49% (with a cut-off of ≥5) and 16% to 45% when using a cut-off of ≥10. We also noted a similar observation when we took the ART status of PLWH into account. Specifically, the prevalence of depression ranged from 8% to 59% among studies whose participants were entirely on ART compared to 6% to 54% among studies with mixed or naïve PLWH. However, studies that used structured interviews such as the MINI, CIDI and DSM-IV to assess for depression were noted to frequently report lower prevalence of

For peer review only - http://bmjopen.bmj.com/site/about/guidelines.xhtml

depression among PLWH50+ (ranging from 6% to 30%) [44, 57] compared to studies that used brief screening scales such as the PHQ-9 (prevalence ranging from 8% to 59%) [27, 59]

Of note, eight studies documented higher prevalence of depression among PLWH50+ than their comparison groups [20, 23, 27, 36, 37, 43, 55, 56]. On the contrary, 11 studies reported a lower prevalence of depression among PLWH50+ than their comparison groups [19, 24, 28, 29, 31, 32, 44, 50, 51, 58, 59]. In comparison, three other studies reported minimal or no differences in depression between PLWH50+ and their comparison groups [34, 42, 57]. In one population-based study conducted in rural South Africa, neither diagnosis nor treatment of HIV was significantly associated with depression when compared with being uninfected [54].

Anxiety was reported in two studies [30, 39]. In one of them [39], PLWH50+ on ART presented with the same proportion of anxiety symptoms with HIV uninfected older adults at 3%. In the other study [30], the prevalence of anxiety disorders among PLWH50+ on ART was 21% compared to 22% in HIV infected young adults (18 – 50 years).

Reported correlates of depression

Correlates of depression among PLWH50+ were reported only in 8 studies [27, 28, 46, 48, 51, 54, 55, 58]. Among sociodemographic variables, older age [27, 54], declining socioeconomic status [55], being unemployed [46], being female [28, 58], HIV status disclosure [48], and urban residency [58] were associated with an elevated risk of depression while being married, employed (full time or part-time work) [54], old age [51], higher educational attainment [48], and belonging to the highest wealth quintile [28] were associated with reduced levels of depression. However, in some studies, household wealth quintile, education levels [51], and age [25] were not significant correlates of depression. In other studies, being a current/former smoker [46], increasing disability scores, decreasing mean handgrip strength, reported back pain, not having hypertension [55], HIV stigma [48], and caregiving (for adult children) [58] were significantly associated with elevated levels of depression. On the other hand, receiving a government grant [58] and resilience [48] were significantly associated with reduced levels of depression. Details of this data are presented in supplementary table 3.

3.3. Cognitive and neurological functioning

These outcomes were reported by 13 studies, as highlighted in Table 1. Of these, five studies quantified the prevalence of HIV-associated neurocognitive disorders (HAND) using different measures [25, 35, 38, 40, 49], two studies reported findings on dementia [21, 60] while 5 studies documented findings on cognitive function/impairment [22, 33, 51, 53, 61]. The remaining study reported the proportion of HIV-associated peripheral neuropathy among PLWH50+ [39].

For peer review only - http://bmjopen.bmj.com/site/about/guidelines.xhtml

Prevalence estimates of reported outcomes

The prevalence of symptomatic HAND among PLWH50+ on ART ranged from 19% [38] to 61% [35]. Two of the HAND studies documented elevated proportions of symptomatic HAND among PLWH50+ on ART compared to their younger counterparts [35, 38]. In rural South Africa, Joska and colleagues examined the prevalence of dementia and its associations in a population survey of 1,150 rural elderly participants. They found no significant differences in the prevalence of dementia between PLWH50+ (86% on ART) and HIV uninfected counterparts 18% vs 11%, respectively [60]. In a different study in rural South Africa, a baseline analysis of the Health and Aging in Africa – A Longitudinal Study of an INDEPTH Community (HAALSI), HIV infected older adults (64% on ART) registered a lower prevalence of cognitive impairment (4%) than their uninfected peers (9%) [53]. In a separate analysis of the same baseline data of the HAALSI cohort [61], HIV infected older adults scored on average 0.06 (95% confidence interval (CI) 0.01–0.12) standard deviation (SD) units higher on a conventional cognitive function measure and 0.02 (95%CI − 0.07 to 0.04) SD units lower when using the Oxford Cognitive Screen-Plus (OCS-Plus) measure than their HIV-negative counterparts.

Correlates of cognitive function/impairment

The correlates of cognitive function/impairment were reported in 7 studies. The key predictors of reduced cognitive function were mainly sociodemographic: older age (≥50 years) [21, 40, 53, 60], living alone [25], being illiterate or having low formal education level [25, 53], being older at the time of HIV diagnosis [25], being female, unmarried, and having low socioeconomic status [53] were associated with a higher risk of impairment. However, in some studies, the effects of age, education, and marital status were not significant [25, 60]. Other correlates of cognitive impairment included the presence of depressive symptoms [60], a history of cardiovascular conditions [53], and a history of falls in the past 12 months [33]. HIV specific factors (such as nadir CD4 count, ART use) [25, 61] and vascular risk factors (such as smoking status, body mass index and blood pressure) [25] were not associated with cognitive impairment.

Insert Table 1– ***Summary of prevalence estimates and correlates for cognitive impairment/neurological impairment*** – here

For peer review only - http://bmjopen.bmj.com/site/about/guidelines.xhtml

3.4. Frailty/grip strength.

Prevalence estimates

Frailty was reported in three studies [33, 47, 62]. In the first study, a hospital-based cross-sectional study of 145 PLWH50+ on ART in Tanzania, frailty assessment was completed using 3 measures (the Fried frailty phenotype – FFP, the Clinical Frailty Scale – CFS, and Brief Frailty Instrument for Tanzania – B-FIT 2) [47]. In this study, the authors reported low levels of frailty: 3% (when using the FFP method) and 1% (when using the CFS and BFIT-2 methods). The second study documented a similar prevalence of frailty among 292 PLWH50+ *(ART status not reported)* and 322 HIV uninfected peers in rural South Africa (15% vs 18% respectively), using a modified version of the FFP [62]. In the third study, increasing frailty scores were significantly associated with increasing polypharmacy levels in a sample of 411 PLWH50+ on ART in Uganda [33]. Five other studies [18, 45, 57, 63, 64] reported mean handgrip strength as an indicator of frailty, as shown in Table 2.

Reported correlates of frailty

The correlates of frailty were reported in two studies. Being female, unmarried [47] and older [47, 62] were significantly associated with worse frailty scores, and there were no associations between inflammatory biomarkers with frailty [62]. Factors associated with mean handgrip strength were reported in four of the studies. In one, HIV status was significantly associated with weaker handgrip strength [57]. In another study, being female and divorced/widowed were significantly associated with weaker handgrip strength, while having a secondary education level was significantly associated with better handgrip strength. Table 2 gives details of this outcome.

Insert Table 2– ***Summary of prevalence estimates and correlates for frailty and handgrip strength*** – here

3.5. Health-related quality of life

This outcome was reported in 8 studies. Three-quarters of the studies used the World Health Organization Quality of Life (WHOQOL) measure to assess health-related quality of life. The overall mean quality of life scores (out of 100) ranged from 42 to 83 among PLWH50+ on ART. In two studies, PLWH50+ (partially on ART) reported better quality of life scores than their uninfected counterparts [63, 65]. In contrast, four other studies reported a lower quality of life scores [39, 41, 52, 66] among PLWH50+ compared to HIV uninfected older adults or HIV infected young adults.

The correlates of quality of life were reported in only 1 study [65], where having some source of income and being in the upper two wealth quintiles were significantly associated with better quality of life among PLWH50+. Additionally, being female and previously married were associated with reduced odds of good quality of life in PLWH50+. Table 3 gives more details on this outcome.

For peer review only - http://bmjopen.bmj.com/site/about/guidelines.xhtml

Overall, a significant number of the included studies (about 35%) had a low quality score (≤2 – indicating a high risk of bias) [19-22, 26, 29, 31, 35-41, 48, 51, 66]. Specifically, about two-thirds of the included studies did not report participant response rates. Sixteen studies used non-probability sampling methods or did not report their sampling methods. Close to half of the included studies had substantially small samples of PLWH50+ (≤100). Furthermore, a substantial number of studies (about 40%) provided scarce information regarding their measures' reliability and validity. Details of the quality assessment of all included studies are captured in the Supplementary File.

Insert Table 3– ***Summary of results for health-related quality of life*** – here

For peer review only

4.0 DISCUSSION

4.1 Summary of main findings

Our review shows that the research on depression, anxiety, cognitive function, frailty and HRQoL among PLWH50+ in SSA is still scanty and largely concentrated in Uganda, South Africa and Ethiopia. Overall, PLWH50+ presented with wide-ranging prevalence of depression (6% to 59%), cognitive impairment (4% to 61%) and frailty (3% to 15%). Additionally, the correlates of these outcomes were rarely investigated. Nonetheless, those reported were mainly sociodemographic variables, many of which were inconsistent. We find the current evidence inadequate to characterise the real burden and determinants of these outcomes in the region, partly because of the limited number of well-designed studies, heterogeneous findings, and substantial methodological limitations observed in many included studies. Therefore, any policies and programmes about the wellbeing of PLWH50+ in the region have been made on the basis of minimal evidence. The rising numbers of PLWH50+ in SSA and the potentially elevated burden of age-associated comorbidities in this population warrant more research across the region. Such data will provide the evidence needed to develop appropriate policies, programmes, and interventions for the successful aging of PLWH in SSA.

4.2 Depression and anxiety

Depression was the most frequently reported outcome in about half of the included studies. This finding is consistent with the available literature on HIV, which recognises depression as the most commonly occurring psychiatric complication in this population [67]. However, only two studies investigated anxiety, a common mental disorder with a similar prevalence to those seen in depression [68]. The under investigation of anxiety disorders is of particular concern because accumulating research involving PLWH50+ in HICs has documented elevated anxiety levels, as high as 65% [68]. More research is needed on this outcome in the region.

Our review also noted wide-ranging prevalences of depression and anxiety. This finding corroborates previous review findings on the prevalence of common mental disorders in the region, albeit in younger HIV populations [69]. The observed variation could reflect significant contextual differences across the SSA region, e.g., social-environmental milieu,

For peer review only - http://bmjopen.bmj.com/site/about/guidelines.xhtml

healthcare systems, patterns of the burden of diseases, and mental health resources, which are likely to alter the risk profile of these adults [70]. It could also be attributed to potential differences in study populations, study participants, and the use of diverse measurement tools (including different cut-offs for the same measures). Indeed, in the current review, depression was assessed using at least 10 different scoring systems with diverse cut-off points. Besides, the majority of the studies did not provide information on the reliability and validity of the tools they used. Hence, it is difficult to draw firm conclusions regarding the reliability, validity, and applicability of many of the measures used to report this outcome. The PHQ-9, for instance, was frequently used to report depressive symptoms among the included studies. However, most of the studies relied on previous validation of the tool, the context of which was not given. A recent review examining the validation of brief CMDs screening tools in low and middle-income countries did not support the use of PHQ-9 in these settings. It performed poorly in several clinic populations with lower average education but performed very well in high literate populations [71]. This is especially an important consideration in SSA, where older adults are more likely to have low literacy levels. There is a need for rigorous cultural adaptation of these instruments to generate accurate epidemiological data that will inform proper intervention work among PLWH50+ in SSA.

Despite the observed limitations, the evidence of better mental health [28, 44, 50, 51, 54, 58, 59] as well as cognition [53, 61] frailty/handgrip strength [47, 62, 63] and quality of life [65] among PLWH50+ on ART (mainly among the robust studies with low risk of bias) compared to uninfected older adults in the current review was striking. This observation could be part of an emerging body of evidence from SSA showing that the mental and wellbeing outcomes among PLWH are comparable to those without HIV. The mechanisms responsible for this observation are not well established and require further examination across many settings in SSA. However, it could be attributed to a few plausible reasons, including i) the direct impact of ART on viral replication, inflammatory pathways, and downstream disease pathophysiology, the so-called "ART advantage" [72, 73], ii) a survivorship bias among PLWH50+ who access and remain in HIV care; and iii) beneficial spillover effects of ART programs such as enhanced access to and utilisation of primary care services resulting in early identification and management of comorbid conditions [73-75]. This observation could also be related to the fact that PLWH50+ on ART are more likely to utilise biomedical facilities due to better education and higher socioeconomic status than their HIV uninfected peers. More studies, preferably longitudinal ones, will shed more light on this observation. As it stands, however, this finding offers an interesting contrast to the commonly reported findings from high-income countries, which generally show that PLWH50+ present with worse physical and mental health outcomes compared to age-matched HIV uninfected people [5, 68, 76]. These findings may suggest context-specific differences in the drivers of physical and mental outcomes, including the demographic and risk factor profile as well as the role of engagement in HIV care – among those with and without HIV, especially when comparing low and middle-income to high-income countries. Future studies should explore the extent to which HIV programs could be promoting the wellbeing of PLWH50+ in SSA compared to HIV uninfected older adults in the general population who are likely to bear the brunt of the prevailing poor health systems and primary care systems in the region.

For peer review only - http://bmjopen.bmj.com/site/about/guidelines.xhtml

4.3 HIV-associated cognitive impairment

The neurological involvement in HIV disease progression remains problematic, even in well-controlled PLWH, resulting in poor quality of life. HIV associated cognitive impairment is deemed one of the most prevalent comorbidities in the era of ART. Aging with HIV would appear to impose a synergistic impact, whereby increased comorbidities, such as hypertension, diabetes, or depression, likely contribute to and maintain HIV-associated cognitive impairment. In HICs, PLWH50+ have consistently been shown to have high levels of cognitive impairments, as high as 50%. The present review found a wide-ranging prevalence of HIV-associated cognitive impairment (4% to 61%). Our observation is consistent with the available literature, where the prevalence has been noted to vary widely depending on sources used and appears dependent on the populations studied, and approaches used [77].

Brief cognitive screening tools such as the Mini Mental State Examination and International HIV Dementia Scale (IHDS) were frequently used to document the prevalence of cognitive impairment compared to comprehensive neuropsychological batteries. While the use of the brief screeners in low resource settings is well understood given the resource gap, researchers should exercise caution on the tools they use. Recent reviews evaluating brief screening tools for neurocognitive impairment in HIV/AIDS have indicated that some of the commonly utilized tools, e.g. the IHDS perform poorly, especially in screening milder cognitive impairments [78, 79]. In the current review, information on tool reliability or validity was rarely reported. One of the studies reported a limited diagnostic accuracy of the IHDS. However, the Oxford Cognitive Screen-Plus (OCSPlus), one of the most recent screening tools, is promising. So far, the tool has only been used in South Africa among older adults living with HIV, and its initial evaluation yielded excellent construct and external validity [80]. This tool has the potential to address some of the unique challenges and gaps facing resource-limited settings in screening for HAND, including difficulty in performing long test batteries, limited screening tools and a shortage of clinical staff.

4.4 Frailty

Only two studies quantified the prevalence of frailty among PLWH50+, ranging from 3% to 15%. One of the studies was exclusively conducted among PLWH50+, yielding a low frequency of frailty (3%). In the other study, conducted in rural South Africa, the prevalence of frailty was similar between PLWH50+ and their uninfected counterparts (15% vs 18%, respectively). This finding supports the growing evidence in SSA, which suggest that older on effective ART have similar or better health profiles than their uninfected counterparts, contrary to what has been observed in HICs. At the global stage, the frailty prevalence in SSA appears relatively lower than that of HICs which ranges from 5% to 29% [81]. The population reaching older age in many SSA settings may be relatively fit/active, still working, which may be true of PLWH, potentially favouring better physical health in these individuals. Nonetheless, given the limited number of studies on this outcome in SSA, these findings remain preliminary and inconclusive and highlight the urgent need for more studies to shed more light on these observations.

4.5 Health-related quality of life

For peer review only - http://bmjopen.bmj.com/site/about/guidelines.xhtml

For some time, HRQoL has emerged as a salient indicator of HIV care and an essential target for HIV-related research, aiming to expand the service paradigm continuum [82]. This new quality of life frontier has been proposed as the 'fourth 90' to the original '90-90-90' testing and treatment target championed by UNAIDS: to ensure that 90% of people with viral load suppression have good HRQoL [82]. In the current review, research on HRQoL among PLWH50+ was scanty. Thus, little is known on the risk and protective factors of patient-reported health-related quality of life. Further studies should explore the independent effects of aging-related conditions such as common mental disorders, cognitive impairments and frailty on HRQoL among PLWH50+ in SSA to support healthy aging even in the context of a chronic illness such as HIV infection.

4.6 The correlates of mental and wellbeing outcomes

The correlates of CMDs, cognitive impairment, frailty and HRQoL have been scarcely investigated in SSA. In the current review, the frequently reported correlates were sociodemographic in nature, many of which (e.g. age, formal education levels and wealth status) were inconsistent. However, being female was consistently associated with declining mental health, cognitive function, HRQoL and frailty status. This finding mirrors the results of a recent review of older women's physical and psychological health residing in SSA, which indicated that women were significantly more likely to report poor health compared with men [83]. This finding highlights the need for adequate representation of both sexes in research studies, incorporating a gender lens in their analyses to understand better the unique health challenges that each gender faces. Further studies are also urgently needed in SSA to identify potential risk and protective factors, especially those that could be modified, to suggest how to best integrate the management of these impairments in HIV care guidelines in SSA. Future studies should explore the influence of HIV-related factors, psychosocial risk (e.g. loneliness, ageism, stigma, social support, living arrangements) and other comorbidities on the health and wellbeing of PLWH50+. To promote healthy aging in the context of chronic conditions, such as HIV infection, the psychological and social domains are especially critical as they could be a way to compensate for physiological limitations (that is, multiple chronic conditions) and allow even in the context of disease and disability, to experience optimal quality of life.

4.7 Strengths and Limitations

To our knowledge, this is the first comprehensive review of CMDs, cognitive impairment, frailty and HRQoL among PLWH50+ living in SSA. Our expanded search of 5 online databases and snowballing techniques minimised the risk of missing relevant studies. However, our review should be considered in the context of important limitations. Despite a thorough search strategy, some articles may have been missed, especially in excluding non-English language studies. However, these were very few. The substantial amount of heterogeneity across the studies in terms of, e.g. study design, operationalisation of age, definitions of outcomes, and measures made comparisons difficult and did not allow a meta-analysis to be done. Furthermore, most of the studies recruited PLWH50+ from HIV clinics whose attendees may represent the less-healthy end of the spectrum of service users leading to overestimation of reported outcomes. Conversely, those at the filter end of the cohort may be more able to attend or be proactive concerning their wellbeing, leading to underestimation

For peer review only - http://bmjopen.bmj.com/site/about/guidelines.xhtml

of outcomes. Besides, the substantial methodological limitations observed might have biased the reported findings, hence limiting their external validity.

4.8 Conclusions

Overall, the existing research on the burden and determinants of mental and wellbeing outcomes among PLWH50+ in SSA is scantly addressed. We also noted substantial limitations in many of the included studies, which need to be addressed in future studies. Our findings underscore the need to increase the evidence base of these outcomes in the region. Future studies should: a) explicitly target older persons ≥50 years living with HIV, b) carefully select a comparison group and have sufficient sample sizes for adequate statistical testing, c) expand the geographical distribution of these studies in many SSA settings and use adequately standardised survey tools adapted for the region, d) and generate more data on the risk and determinants of these outcomes using a gender lens. There is also an ongoing need for well-designed longitudinal cohort studies to study the temporal patterns of these outcomes in the region.

Contributors: PNM and AA conceptualised and designed the review. PNM and AM reviewed titles, abstracts and full-text papers for eligibility as well as data extraction. PNM wrote the first draft of the manuscript. AM, CN, RW and AA reviewed and edited the prepared manuscript.

Funding

This work was funded by the Wellcome Trust International Master's Fellowship to PNM (Grant number 208283/Z/17/Z). Further funding supporting this work was from 1) the Medical Research Council (Grant number MR/M025454/1) to AA. This award is jointly funded by the UK Medical Research Council (MRC) and the UK Department for International Development (DFID) under MRC/DFID concordant agreement and is also part of the EDCTP2 program supported by the European Union; 2) DELTAS Africa Initiative [DEL-15-003]. The DELTAS Africa Initiative is an independent funding scheme of the African Academy of Sciences (AAS) 's Alliance for Accelerating Excellence in Science in Africa (AESA) and supported by the New Partnership for Africa's Development Planning and Coordinating Agency (NEPAD Agency) with funding from the Wellcome Trust [107769/Z/10/Z] and the UK government. The funders did not have a role in the design and conduct of the study or interpretation of study findings. The views expressed in this publication are those of the author(s) and not necessarily those of AAS, NEPAD Agency, Wellcome Trust or the UK government. For the purpose of Open Access, the author has applied a CC-BY public copyright license to any accepted manuscript version arising from this submission.

Competing interests: The authors have no potential conflict of interest to declare.

Patient consent: Not required

Data sharing statement: No original data were generated for this study. All data underlying the results are available as part of the article, and no additional source data are required.

For peer review only - http://bmjopen.bmj.com/site/about/guidelines.xhtml

Acknowledgement

The authors would like to thank the Director of Kenya Medical Research Institute for granting permission to publish this work

Ethics Statement

This work is part of a PhD project to the first author. The project has obtained ethical clearance from the Kenya Medical Research Institute – Scientific and Ethics Review Unit (KEMRI/SERU/CGMR-C/152/3804).

References

1. United Nations, *World population ageing 2019: Highlights*, in *ST/ESA/SER. A/430*. 2019.
2. Autenrieth, C.S., et al., *Global and regional trends of people living with HIV aged 50 and over: Estimates and projections for 2000–2020.* PloS one, 2018. **13**(11): p. e0207005.
3. O'Brien, K.K., et al., *Research priorities for rehabilitation and aging with HIV: a framework from the Canada-International HIV and Rehabilitation Research Collaborative (CIHRRC).* AIDS Research and Therapy, 2020. **17**: p. 1-13.
4. Sabin, C.A. and P. Reiss, *Epidemiology of ageing with HIV: what can we learn from cohorts?* Aids, 2017. **31**: p. S121-S128.
5. Brennan-Ing, M., J. Ramirez-Valles, and A. Tax, *Aging With HIV: Health Policy and Advocacy Priorities.* Health Education & Behavior, 2021. **48**(1): p. 5-8.
6. Milic, J., et al., *European cohorts of older HIV adults: POPPY, AGE h IV, GEPPO, COBRA and FUNCFRAIL.* European Geriatric Medicine, 2019. **10**(2): p. 247-257.
7. Brothers, T.D. and K. Rockwood, *Frailty: a new vulnerability indicator in people aging with HIV.* European Geriatric Medicine, 2019. **10**(2): p. 219-226.
8. Gustafson, D., et al., *Frailty and constellations of factors in aging HIV-infected and uninfected women-the women's interagency HIV study.* The Journal of frailty & aging, 2016. **5**(1): p. 43.
9. Levett, T., *Frailty Prevalence and Predictors in Older Adults with HIV*. 2017, University of Brighton.
10. Saka, S., F. Oosthuizen, and M. Nlooto, *national policies and older people's healthcare in Sub-Saharan Africa: a scoping review.* Annals of global health, 2019. **85**(1).
11. Nabalamba, A. and M. Chikoko, *Aging population challenges in Africa*. 2011: African Development Bank, Chief Economist Complex.
12. Joint United Nations Programme on HIV/AIDS, *The Gap Report. Geneva, Switzerland: UNAIDS*. 2014.
13. World Health Organization. *Health statistics and information systems: SAGE HIV studies*. 2020 [cited 2020 June 23]; Available from: https://www.who.int/healthinfo/sage/hiv_studies/en/.
14. Gómez-Olivé, F.X., et al., *Cohort profile: health and ageing in Africa: a longitudinal study of an INDEPTH community in South Africa (HAALSI).* International journal of epidemiology, 2018. **47**(3): p. 689-690j.
15. National Institutes of Health. *Ugandan Non-Communicable Diseases and Aging Cohort (UGANDAC)*. 2020 [cited 2020 June 23]; Available from: https://clinicaltrials.gov/ct2/show/NCT02445079.
16. Page, M.J., et al., *PRISMA 2020 explanation and elaboration: updated guidance and exemplars for reporting systematic reviews.* bmj, 2021. **372**.
17. Stang, A., *Critical evaluation of the Newcastle-Ottawa scale for the assessment of the quality of nonrandomized studies in meta-analyses.* European journal of epidemiology, 2010. **25**(9): p. 603-605.

For peer review only - http://bmjopen.bmj.com/site/about/guidelines.xhtml

18. Filteau, S., et al., *Nutritional status is the major factor affecting grip strength of African HIV patients before and during antiretroviral treatment.* Trop Med Int Health, 2017. **22**(10): p. 1302-1313.
19. Akena, D., et al., *The association between aids related stigma and major depressive disorder among HIV-positive individuals in Uganda.* PLoS One, 2012. **7**(11): p. e48671.
20. Asangbeh, S.L., et al., *Predictors of depression among patients on art in a rural health district in North West Cameroon.* AIDS Care, 2016. **28**(2): p. 205-8.
21. Atashili, J., et al., *Prevalence, characteristics and correlates of a positive-dementia screen in patients on antiretroviral therapy in Bamenda, Cameroon: a cross-sectional study.* BMC Neurol, 2013. **13**: p. 86.
22. Cassimjee, N. and P.K. Motswai, *Neuropsychological profiles of adults and older adults with HIV.* South African Journal of Psychology, 2017. **47**(1): p. 35-45.
23. Cholera, R., et al., *Depression and Engagement in Care Among Newly Diagnosed HIV-Infected Adults in Johannesburg, South Africa.* AIDS & Behavior, 2017. **21**(6): p. 1632-1640.
24. Duko, B., et al., *Prevalence and associated factors of depression among patients with HIV/AIDS in Hawassa, Ethiopia, cross-sectional study.* Annals of General Psychiatry, 2018. **17**.
25. Eaton, P., et al., *Risk factors for symptomatic HIV-associated neurocognitive disorder in adults aged 50 and over attending a HIV clinic in Tanzania.* Int J Geriatr Psychiatry, 2020.
26. Harding, R., et al., *Quality of life and wellbeing among HIV outpatients in East Africa: a multicentre observational study.* BMC Infect Dis, 2014. **14**: p. 613.
27. Kaharuza, F.M., et al., *Depression and CD4 cell count among persons with HIV infection in Uganda.* AIDS Behav, 2006. **10**(4 Suppl): p. S105-11.
28. Manne-Goehler, J., et al., *Depressive symptoms before and after antiretroviral therapy initiation among older-aged individuals in rural Uganda.* AIDS and Behavior, 2019. **23**(3): p. 564-571.
29. Musinguzi, K., et al., *Association between major depressive disorder and pro-inflammatory cytokines and acute phase proteins among HIV-1 positive patients in Uganda.* BMC Immunol, 2018. **19**(1): p. 1.
30. Olagunju, A.T., et al., *Factors associated with anxiety disorders among HIV-positive attendees of an HIV clinic in Lagos, Nigeria.* Int J STD AIDS, 2012. **23**(6): p. 389-93.
31. Olley, B.O., et al., *Psychosocial factors predicting severity of depression among treatment-seeking HIV/AIDS patients: A multi-site Nigerian study.* Niger J Clin Pract, 2017. **20**(3): p. 296-302.
32. Shumba, C., et al., *Prevalence of Depressive Symptoms Amongst Highly Active Antiretroviral Therapy (HAART) Patients in AIDSRelief Uganda.* J Public Health Afr, 2013. **4**(2): p. e19.
33. Ssonko, M., et al., *Polypharmacy among HIV positive older adults on anti-retroviral therapy attending an urban clinic in Uganda.* BMC Geriatr, 2018. **18**(1): p. 125.
34. Tesfaw, G., et al., *Prevalence and correlates of depression and anxiety among patients with HIV on-follow up at Alert Hospital, Addis Ababa, Ethiopia.* BMC Psychiatry, 2016. **16**(1): p. 368.
35. Tsegaw, M., et al., *Screening HIV-associated neurocognitive disorders (HAND) among HIV positive patients attending antiretroviral therapy in South Wollo, Ethiopia.* J Psychiatr Res, 2017. **85**: p. 37-41.
36. Berhe, H. and A. Bayray, *Prevalence of depression and associated factors among people living with HIV/AIDS in tigray, North Ethiopia: A cross sectional hospital based study.* International Journal of Pharmaceutical Sciences and Research, 2013. **4**(2): p. 765.
37. Eshetu, D.A., et al., *Prevalence of depression and associated factors among HIV/AIDS Patients attending ART Clinic at Debrebirhan referral hospital, North Showa, Amhara Region, Ethiopia.* Clin Psychiatry, 2015. **1**(1): p. 1-7.

For peer review only - http://bmjopen.bmj.com/site/about/guidelines.xhtml

38. Mugendi, A., et al., *Prevalence and Correlates of Neurocognitive Disorders among HIV patients on antiretroviral therapy at a Kenyan Hospital.* Neurology research international, 2019. **2019**.
39. Obimakinde, A.M., et al., *Going Beyond Giving Antiretroviral Therapy: Multimorbidity in Older People Aging with HIV in Nigeria.* AIDS research and human retroviruses, 2020. **36**(3): p. 180-185.
40. Oumar, G., et al., *Neurocognitive Disorders in People Living with Human Immunodeficiency Virus Aged 50 Years Old and More in Yalgado Ouédraogo Teaching Hospital.* American Journal of Internal Medicine, 2020. **8**(4): p. 177-181.
41. Parcesepe, A.M., et al., *Gender, HIV-related stigma, and health-related quality of life among adults enrolling in HIV Care in Tanzania.* AIDS and Behavior, 2020. **24**(1): p. 142-150.
42. Torgersen, J., et al., *Impact of efavirenz metabolism on loss to care in older HIV+ Africans.* European Journal of Drug Metabolism and Pharmacokinetics, 2019. **44**(2): p. 179-187.
43. Abadiga, M., *Depression and its associated factors among HIV/AIDS patients attending ART clinics at Gimbi General hospital, West Ethiopia, 2018.* BMC Research Notes, 2019. **12**(1): p. 527.
44. Asrat, B., et al., *Major depressive disorder and its association with adherence to antiretroviral therapy and quality of life: cross-sectional survey of people living with HIV/AIDS in Northwest Ethiopia.* BMC psychiatry, 2020. **20**(1): p. 1-13.
45. Bernard, C., et al., *Prevalence and factors associated with physical function limitation in older West African people living with HIV.* PloS one, 2020. **15**(10): p. e0240906.
46. Bernard, C., et al., *Prevalence and factors associated with severe depressive symptoms in older west African people living with HIV.* BMC psychiatry, 2020. **20**(1): p. 1-11.
47. Bristow, C., et al., *Low levels of frailty in HIV-positive older adults on antiretroviral therapy in northern Tanzania.* Journal of NeuroVirology, 2021: p. 1-12.
48. Kalomo, E.N., et al., *HIV stigma, resilience and depressive symptoms among older adults living with HIV in rural Namibia.* African Journal of AIDS Research, 2020. **19**(3): p. 198-205.
49. Kellett-Wright, J., et al., *Screening for HIV-Associated Neurocognitive Disorder (HAND) in Adults Aged 50 and Over Attending a Government HIV Clinic in Kilimanjaro, Tanzania. Comparison of the International HIV Dementia Scale (IHDS) and IDEA Six Item Dementia Screen.* AIDS and Behavior, 2020: p. 1-12.
50. Motumma, A., et al., *Prevalence and associated factors of common mental disorders among adult patients attending HIV follow up service in Harar town, Eastern Ethiopia: a cross-sectional study.* BMC psychology, 2019. **7**(1): p. 1-9.
51. Moucheraud, C., et al., *A Multi-Dimensional Characterization of Aging and Wellbeing Among HIV-Positive Adults in Malawi.* AIDS and Behavior, 2020: p. 1-11.
52. Yaya, I., et al., *Health-related quality of life among people living with HIV/AIDS in Togo: individuals and contextual effects.* BMC Research Notes, 2019. **12**(1): p. 140.
53. Kobayashi, L.C., et al., *Cognitive function and impairment in older, rural south African adults: evidence from "health and aging in Africa: a longitudinal study of an INDEPTH Community in Rural South Africa".* Neuroepidemiology, 2019. **52**(1-2): p. 32-40.
54. Geldsetzer, P., et al., *Depressive symptoms and their relation to age and chronic diseases among middle-aged and older adults in rural South Africa.* The Journals of Gerontology: Series A: Biological Sciences and Medical Sciences, 2019. **74**(6): p. 957-963.
55. Kinyanda, E., et al., *Risk of major depressive disorder among older persons living in HIV-endemic central and southwestern Uganda.* AIDS Care, 2016. **28**(12): p. 1516-1521.
56. Mugisha, J.O., et al., *Chronic disease, risk factors and disability in adults aged 50 and above living with and without HIV: findings from the Wellbeing of Older People Study in Uganda.* Glob Health Action, 2016. **9**: p. 31098.
57. Negin, J., et al., *Prevalence of HIV and chronic comorbidities among older adults.* Aids, 2012. **26 Suppl 1**(0 1): p. S55-63.

For peer review only

For peer review only - http://bmjopen.bmj.com/site/about/guidelines.xhtml

58. Nyirenda, M., et al., *Prevalence and correlates of depression among HIV-infected and-affected older people in rural South Africa.* Journal of affective disorders, 2013. **151**(1): p. 31-38.
59. Rohr, J.K., et al., *HIV treatment cascade for older adults in rural South Africa.* Sexually Transmitted Infections, 2020. **96**(4): p. 271-276.
60. Joska, J.A., et al., *Prevalence of HIV-1 Infection in an elderly rural population and associations with neurocognitive impairment.* Aids, 2019. **33**(11): p. 1765-1771.
61. Asiimwe, S.B., et al., *Cognitive differences associated with HIV serostatus and antiretroviral therapy use in a population-based sample of older adults in South Africa.* Scientific reports, 2020. **10**(1): p. 1-14.
62. Edwards, A., et al., *HIV serostatus, inflammatory biomarkers and the frailty phenotype among older people in rural KwaZulu-Natal, South Africa.* African Journal of AIDS Research, 2020. **19**(3): p. 177-185.
63. Mugisha, J., et al., *Caregiving responsibilities and burden among older people by HIV status and other determinants in Uganda.* AIDS Care, 2013. **25**(11): p. 1341-8.
64. Scholten, F., et al., *Health and functional status among older people with HIV/AIDS in Uganda.* BMC Public Health, 2011. **11**: p. 886.
65. Nyirenda, M., et al., *An investigation of factors associated with the health and well-being of HIV-infected or HIV-affected older people in rural South Africa.* BMC Public Health, 2012. **12**(1): p. 259-259.
66. Maniragaba, F., et al., *Predictors of quality of life of older persons in rural Uganda: A cross sectional study.* AAS Open Res, 2018. **1**: p. 22.
67. Nanni, M.G., et al., *Depression in HIV infected patients: a review.* Current psychiatry reports, 2015. **17**(1): p. 530.
68. Brennan-Ing, M., *Emerging issues in HIV and aging*. 2020.
69. Too, E.K., et al., *Prevalence and factors associated with common mental disorders in young people living with HIV in sub-Saharan Africa: a systematic review.* Journal of the International AIDS Society, 2021. **24**: p. e25705.
70. Nyirenda, M., et al., *Health, wellbeing, and disability among older people infected or affected by HIV in Uganda and South Africa.* Global health action, 2013. **6**(1): p. 19201.
71. Ali, G.-C., G. Ryan, and M.J. De Silva, *Validated screening tools for common mental disorders in low and middle income countries: a systematic review.* PloS one, 2016. **11**(6): p. e0156939.
72. Pala, A.N., et al., *Subtypes of depressive symptoms and inflammatory biomarkers: An exploratory study on a sample of HIV-positive patients.* Brain, behavior, and immunity, 2016. **56**: p. 105-113.
73. Manne-Goehler, J., et al., *The ART advantage: healthcare utilization for diabetes and hypertension in rural South Africa.* Journal of acquired immune deficiency syndromes (1999), 2017. **75**(5): p. 561.
74. Feinstein, M.J., et al., *Ideal cardiovascular health and carotid atherosclerosis in a mixed cohort of HIV-infected and uninfected Ugandans.* AIDS research and human retroviruses, 2017. **33**(1): p. 49-56.
75. Wouters, E., et al., *Physical and emotional health outcomes after 12 months of public-sector antiretroviral treatment in the Free State Province of South Africa: a longitudinal study using structural equation modelling.* BMC Public Health, 2009. **9**(1): p. 103.
76. Langebeek, N., et al., *Impact of comorbidity and ageing on health-related quality of life in HIV-positive and HIV-negative individuals.* Aids, 2017. **31**(10): p. 1471-1481.
77. Alford, K. and J. Vera, *Cognitive impairment in people living with HIV in the ART era: a review.* British medical bulletin, 2018. **127**(1): p. 55-68.
78. Zipursky, A.R., et al., *Evaluation of brief screening tools for neurocognitive impairment in HIV/AIDS: a systematic review of the literature.* AIDS (London, England), 2013. **27**(15): p. 2385.

For peer review only

For peer review only - http://bmjopen.bmj.com/site/about/guidelines.xhtml

79. Mwangala, P.N., et al., *Screening tools for HIV-associated neurocognitive disorders among adults living with HIV in sub-Saharan Africa: A scoping review.* AAS open research, 2018. **1**.
80. Humphreys, G.W., et al., *Cognitive function in low-income and low-literacy settings: Validation of the tablet-based Oxford Cognitive Screen in the Health and Aging in Africa: A Longitudinal Study of an INDEPTH community in South Africa (HAALSI).* The Journals of Gerontology: Series B, 2017. **72**(1): p. 38-50.
81. Levett, T.J., et al., *Systematic review of prevalence and predictors of frailty in individuals with human immunodeficiency virus.* Journal of the American Geriatrics Society, 2016. **64**(5): p. 1006-1014.
82. Lazarus, J.V., et al., *Beyond viral suppression of HIV–the new quality of life frontier.* BMC medicine, 2016. **14**(1): p. 94.
83. Mangipudi, S., T. Cosco, and S. Harper, *A systematic review of physical and psychological health and wellbeing of older women in Sub-Saharan Africa.* Journal of Public Health, 2020. **42**(2): p. 294-303.

For peer review only

For peer review only - http://bmjopen.bmj.com/site/about/guidelines.xhtml

Table 1: Summary of prevalence estimates and correlates for cognitive impairment/neurological impairment

Author, publication year & country	Study design	Sample size	Treatment status of PLWH	Assessment tool used	Cut-off score or diagnosis criteria	Information on local tool validation	Prevalence estimates/other results for PLWH50+	Correlates reported
Studies reporting the prevalence of HIV-associated neurocognitive disorders (HAND) or cognitive impairment								
Kellett-Wright, J., et al. (2020) ; Tanzania [49]	Cross-sectional	235 PLWH50+	95.5% on ART	International HIV Dementia Scale (IHDS) and IDEA (Six Item Dementia Screen)	Frascati criteria with a detailed consensus panel discussion	Diagnostic accuracy (area under the receiver operating characteristic curve: 0.64–0.67 for IHDS and 0.65–0.71 for IDEA	Prevalence of symptomatic HAND (MND and HAD) was 25.3%.	Not reported
Eaton, P., et al. (2020); Tanzania [25]	Cross-sectional	253 PLWH50+	94.8% on ART	Comprehensive NP battery	Frascati criteria for classifying HAND	NR	Prevalence of symptomatic HAND (HAD and MND) was 21.7%.	PLWH were at higher risk of having symptomatic HAND if they lived alone [OR = 2.6], were illiterate (OR= 3.2) or older at the time of HIV diagnosis (OR = 1.1). HIV-specific factors were not related to symptomatic HAND. Vascular risk factors, such as smoking status, were not associated with symptomatic HAND.
Oumar, G., et al. (2020); Burkina Faso [40]	Cross-sectional	102 PLWH50+	All on ART	Mini Mental State Examination	Frascati criteria for classifying HAND	NR	23.5% had neurocognitive disorders	In a multivariate analysis, age (OR=4.6) and educational status (OR=2.6) were associated with neurocognitive disorders.
Mugendi, A., et al. (2019); Kenya [38]	Cross-sectional	64 PLWH50+; 281 PLWH (20-49 years)	All on ART	Montreal Cognitive Assessment (MoCA) and IHDS	Combined scores of the 2 tools: a score of ≤26 on MOCA and ≤10 on the IHDS.	NR	Among PLWH50+, symptomatic HAND was 19.2%	Reported correlates not aggregated by age.
Tsegaw, M., et al. (2017); Ethiopia [35]	Cross-sectional	67PLWH56+; 526 PLWH (18-55 years)	All on ART	International HIV Dementia Scale	≤9.5	NR	61.2% among PLWH56+ had HAND	Reported correlates not aggregated by age.
Kobayashi, L. C., et al. (2019); South Africa [53]	Population survey	1,048 PLWH40+; 4,011 HIV uninfected older adults ≥40 years	63.9% on ART	Brief cognitive battery (orientation, immediate & delayed recall).	≤ 1.5 SDs below the mean composite time orientation and memory score.	Locally adapted	4.0% of PLWH40+ had cognitive impairment compared to 9.0% of uninfected older adults ≥40 years	Independent predictors of lower cognitive score were being older, female, unmarried, not working, having low formal education, low socioeconomic status, and having a history of cardiovascular conditions.
Ssonko, M., et al. (2018); Uganda [33]	Cross-sectional	411 PLWH50+	All on ART	Folstein's Mini-Mental State Examination	NR	NR	Mild cognitive impairment was associated with falls in the previous 12 months.	NR

For peer review only

For peer review only - http://bmjopen.bmj.com/site/about/guidelines.xhtml

Author, publication year & country	Study design	Sample size	Treatment status of PLWH	Assessment tool used	Cut-off score or diagnosis criteria	Information on local tool validation	Prevalence estimates/other results for PLWH50+	Correlates reported
Studies reporting neuropsychological test scores								
Asiimwe, S. B., et al. (2020); South Africa [61]	Population survey	1048 PLWH40+; 3512 HIV uninfected older adults ≥40 years	68.6% on ART	A battery of conventional instruments and the Oxford Cognitive Screen-Plus (OCS-Plus)	Summarised mean cognitive scores between participants with and without HIV on the two measures.	Previously validated in the study setting	PLWH scored on average 0.06 (95% CI 0.01–0.12) standard deviation (SD) units higher on the conventional cognitive function measure and 0.02 (95% CI − 0.07 to 0.04) SD units lower on the OCS-Plus measure than HIV-negative participants.	ART use was non-significantly associated with better cognitive outcomes among PLWH in both instruments.
Moucheraud, C., et al. (2020); Malawi [51]	Cross-sectional	74 PLWH50+ and 60 young PLWH (30-49) years	All on ART	Brief cognitive battery (MMSE)	NR	NR	Mean cognitive scores: 16.0 among PLWH50+ versus 17.4 among young PLWH.	NR
Cassimjee, N. and P. K. Motswai (2017); South Africa [22]	Cross-sectional	33 PLWH50+; 17 HIV uninfected older adults [mean age 52.2]	NR	Dementia Rating Scale-2, the Stroop Colour and Word Test, the Symbol Digits Test and the D-KEFS Trail Making Test.	N/A	NR	PLWH, in comparison to the uninfected group, had significantly poorer performance profiles in global cognitive functioning, memory, executive functioning, psychomotor functioning, and processing speed.	Not reported
Studies reporting dementia								
Joska, J. A., et al. (2019); South Africa [60]	Population survey	55 PLWH50+; 1,095 HIV uninfected older adults ≥50 years	86.1% on ART	Brief Community Screening Instrument for Dementia	NR	Previously validated	18.2% of PLWH50+ had dementia compared to 10.7 % of the uninfected older adults ≥50 years	Dementia status was associated with HIV infection (OR= 2.2, CI=1–4.5), older age (OR =1.0, CI=1.0–1.1) and f depressive symptoms (OR 2.7, CI=1.8–3.9).
Atashili, J., et al. (2013); Cameroon [21]	Cross-sectional	400 HIV infected adults (20-53 years old)	All on ART	International HIV Dementia Scale (IHDS)	≤10 on IHDS	Not validated	The odds of having a positive dementia screen increased progressively with increasing participants' age,	Not aggregated by age
Studies reporting HIV-associated neuropathy								
Obimakinde, A. M., et al. (2020); Nigeria [39]	Cross-sectional	62 PLWH60+; 162 HIV uninfected older adults ≥60 years	All on ART	Extracted from participants medical notes	Clinical notes	NR	19.0% in PLWH60+ had peripheral neuropathy	NR

Notes: ART – *Antiretroviral treatment*; **HAD –** *HIV-associated* dementia; **HAND –** *HIV-associated neurocognitive disorders*; **IDEA –** Item Dementia Screen**; IHDS** – *International HIV dementia scale*; **MMSE –** *Mini-mental Status Examination*; **MND** –*Mild neurocognitive* disorder; **NA** – *Not* applicable; **NR** – *Not* reported; SD – *Standard* deviation; **OR –** *Odds ratio*; **PLWH –** *People living with HIV*; **PLWH40+ -** *People living with HIV ≥40* years; **PLWH50+ –** *People living with HIV ≥50 years old*

For peer review only - http://bmjopen.bmj.com/site/about/guidelines.xhtml

Table 2: Summary of prevalence estimates and correlates for frailty and handgrip strength

Author, publication year & country	Study design	Sample size	Treatment status of PLWH	Assessment tool used	Cut-off score	Tool validation	Prevalence estimates/other results for PLWH50+	Correlates reported
**Studies that assessed frailty comprehensively**								
Bristow, C., et al. (2021); Tanzania. [47]	Cross-sectional	145 PLWH50+	99.3% on ART	Fried frailty phenotype (FFP), Clinical Frailty Scale (CFS) and B-FIT 2).	**FFP:** A score of 1–2 was pre-frail, and a score of ≥ 3 was frail; **B-FIT** (A score of 8/20); **CFS** (a score of 5–9)	BFIT and FFP previously validated in Tanzania.	2.8% (95% CI 0.09–5.4%) when using **the FFP.** 0.68% (CI − 0.66–2.04%) when using **the CFS or B-FIT 2).**	FFP frailty was associated with female gender (p = 0.006), marital status (p = 0.007) and age (p = 0.038). Weight loss was the most common FFP domain failure.
Edwards, A., et al. (2020); South Africa [62]	Cross-sectional	292 PLWH50+ and 322 HIV uninfected adults (≥50) years.	NR	Fried Frailty Phenotype (FFP)	Presence of ≥3 of the 5 FFP phenotypes	Adapted in the local population	Frailty was similar for HIV negative and PWH (17.7% vs 14.7%, p = 0.72) respectively as was pre-frailty status (66.5% vs 63.4%).	The prevalence of frailty increased with age. There was no association between any of the inflammatory biomarkers and frailty and pre-frailty.
Ssonko, M., et al. (2018); Uganda [33]	Cross-sectional	411 PLWH50+	All on ART	Self-report 40 -item frailty index questionnaire	NR	NR	Frailty index scores of 5 to 6 (PR = 10.6, 95% C.I. 1.4, 78, p = 0.02), and 7 or more (PR = 17.4, 95% C.I. 2.4, 126.5, p = 0.005) were associated with polypharmacy.	Increasing Frailty score was associated with increasing levels of polypharmacy.
Studies that assessed handgrip strength								
Bernard, C., et al. (2020); Coˆte d'Ivoire & Senegal [45]	Cross-sectional	333 PLWH50+	All on ART	Calibrated Jamar hydraulic hand dynamometer	A performance less to the median obtained in each gender group (male/female)	Not reported	43.2% of women had low grip strength. 42.5% of men had low grip strength.	NR
Filteau, S., et al. (2017); Zambia and Tanzania [18]	Randomized trial	PLWH50+: 148 at baseline; 72 at 12wks, and 50 at 2-3 years ART	ART naïve at baseline	Digital handgrip dynamometer	NA	NR	Mean grip strength change: 18.7 kgs at bassline, 20.4 kgs at 12 weeks and 23.1 kgs at 2-3 years.	Reported correlates not aggregated by age.
Mugisha, J., et al. (2013); Uganda [63]	Cross-sectional	198 PLWH50+; 311 HIV uninfected adults ≥50 years	51.0% on ART	Digital handgrip dynamometer	NA	NR	25.7 among PLWH50+; and 22.1 among the uninfected older adults ≥50 years.	NR

For peer review only

Author, publication year & country	Study design	Sample size	Treatment status of PLWH	Assessment tool used	Cut-off score	Tool validation	Prevalence estimates/other results for PLWH50+	Correlates reported
Negin, J., et al. (2012); South Africa [57]	Cross-sectional	142 PLWH50+; 2,722 HIV uninfected older adults ≥50 years	NR	Digital handgrip dynamometer	NA	Locally validated	Mean Grip strength in PLWH50+ (33.5 kg) was weaker than HIV-uninfected individuals (38.3 kg).	When controlling for age and sex among those aged 50 years and older, HIV status was significantly associated with weaker grip strength.
Scholten, F., et al. (2011); Uganda [64]	Cross-sectional	189 PLWH50+; 2017 HIV affected 50+; and 104 HIV uninfected older adults ≥50 years	51.0% on ART	Digital handgrip dynamometer	NA	Locally adapted	25.8 among PLWH50+; and 25.3 among HIV affected 50+ and 25.2 among the uninfected older adults ≥50 years.	Women scored poorly than men. Married respondents had significantly better scores than widowed and divorced respondents. Respondents with at least secondary education had significantly better scores.

Notes: ART – *Antiretroviral treatment*; **B-FIT 2** – *Brief Frailty Instrument of Tanzania*; **CFS** – *Clinical Frailty Scale*; **FFP** – *Fried Frailty Phenotype*; **NA** – *Not* applicable; **NP** – Neuropsychological; **NR** – *Not* reported; **PLWH –** *People living with HIV*; **PLWH50+ –** *People living with HIV ≥50 years old*; **PR** – *Prevalence ratio*

For peer review only - http://bmjopen.bmj.com/site/about/guidelines.xhtml

Table 3: Summary of results for health-related quality of life

Author, publication year & country	Study design	Sample size	Treatment status of PLWH	Assessment tool used	Cut-off score	Information on local tool validation	Prevalence estimates/other results	Correlates reported
**Studies that utilized different versions of the World Health Organization Quality-of-Life Scale (WHOQOL)**								
Obimakinde, A. M., et al. (2020); Nigeria [39]	Cross-sectional	62 PLWH60+; 124 HIV uninfected older adults ≥60 years	All on ART	8-item WHOQoL	NA	NR	Mean QoL (SD): 82.7 (8.9) among PLWH50+; 86.2 (6.5) in the uninfected adults ≥50 years.	NR
Mugisha, J., et al. (2013); Uganda [63]	Cross-sectional	198 PLWH50+; 311 HIV uninfected older adults ≥50 years	51.0% on ART	8-Item WHOQoL	NA	NR	Mean QoL scores: 57.2 among PLWH50+; and 45.3 in the uninfected older adults ≥50 years	NR
Nyirenda, M., et al. (2013); Uganda and South Africa [70]	Cross-sectional	400 PLWH50+; 732 HIV uninfected older adults ≥50 years	About 22% on ART	8-Item WHOQoL	NA	Locally adapted and validated	Older people in South Africa were more likely to have a better quality of life (aOR 2.15, 95% CI 1.60–2.90) than in Uganda.	NR
Nyirenda, M., et al. (2012); South Africa [65]	Cross-sectional	203 PLWH50+; 219 HIV uninfected older adults ≥50 years	23.7% on ART	8-Item WHOQoL	NA	Locally adapted	Mean QoL scores: 62.5 among PLWH50+; and 59.4 in the uninfected older adults ≥50 years.	Being female and having been previously married was associated with lower odds of good QoL in PLWH50+
Yaya, I., et al. (2019) ; Togo [52]	Cross-sectional	154 PLWH50+; 726 PLWH (15-49 years)	90.5% on ART	31- Item WHOQOL-HIV BREF	≥77.3 was considered good quality of life.	NR	72.7% of PLWH50+ had a good global quality of life compared to 77.3% in the younger PLWH.	Correlates not aggregated by age
Maniragaba, F., et al. (2018) ; Uganda [66]	Cross-sectional	28 PLWH50+; 884 HIV uninfected older adults ≥60 years	NR	An adapted version of the WHOQOL-OLD	An index (good, fair and poor quality of life) .	NR	PLWH had poor quality of life (OR=0.45; CI=0.22-0.93) compared to those who were HIV uninfected.	Not aggregated by HIV status
Studies that used other measures of quality of life								
Parcesepe, A. M., et al. (2020); Tanzania [41]	Cross-sectional	82 PLWH50+; 824 PLWH (18-49 years)	ART naïve	HIV/AIDS-Targeted Quality of Life Scale	NA	Previously validated in other SSA settings	Mean QoL score: 68 among PLWH50+, and 72.4 among PLWH (18-49 years)	Reported correlates not aggregated by age.
Harding, R., et al. (2014); Kenya and Uganda [26]	Cross-sectional	1,337 adults aged 18-70 years *(number of PLWH50+ NR)*	49.9% on ART	Medical Outcome Scale -HIV (MOS-HIV)	NA	Previously validated in other SSA setting	**Mean Mental Health score:** 46.4 among PLWH50+; 46.2 in PLWH (18-49 years) **Physical health score**: 42.9 among PLWH50+; and 44.8 in PLWH (18-49 years)	Reported correlates not aggregated by age.

Notes: ART – *Antiretroviral treatment*; **AOR –** *Adjusted odds ratio*; **OR –** *Odds ratio*; **CI –** *Confidence interval*; **NA –** *Not* applicable; **NR –** *Not reported*; **PLWH –** *People living with HIV*; **PLWH50+ –** *People living with HIV ≥50 years old*; **QoL –** *Quality of* life; **SSA** – *sub-Saharan* Africa; **SD –** *Standard deviation*; **WHOQOL** – *World Health Organization Quality of life measure*

For peer review only - http://bmjopen.bmj.com/site/about/guidelines.xhtml

Figure 1: Identification of eligible studies

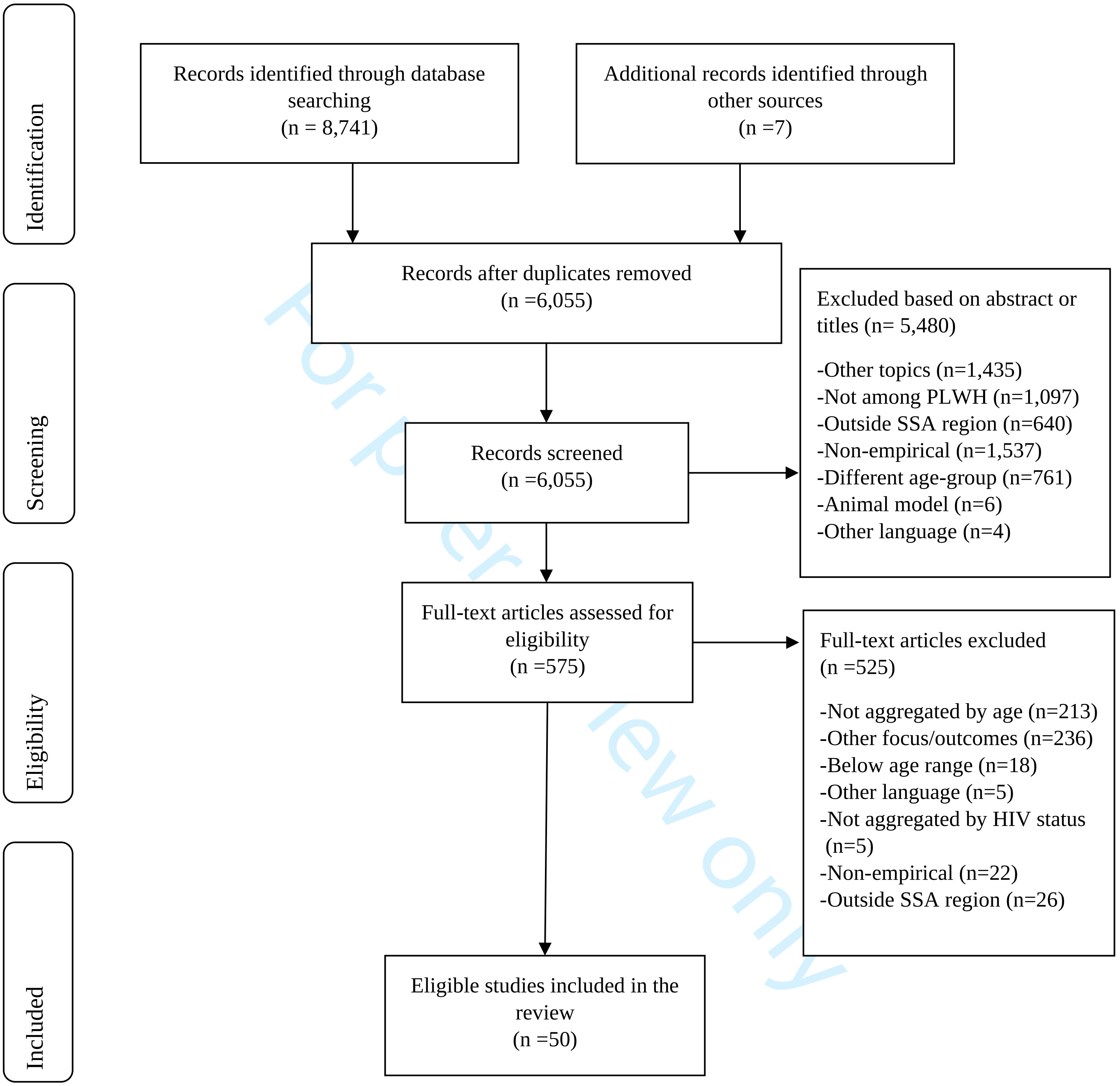

Figure 2: Geographical distribution of the included studies within SSA

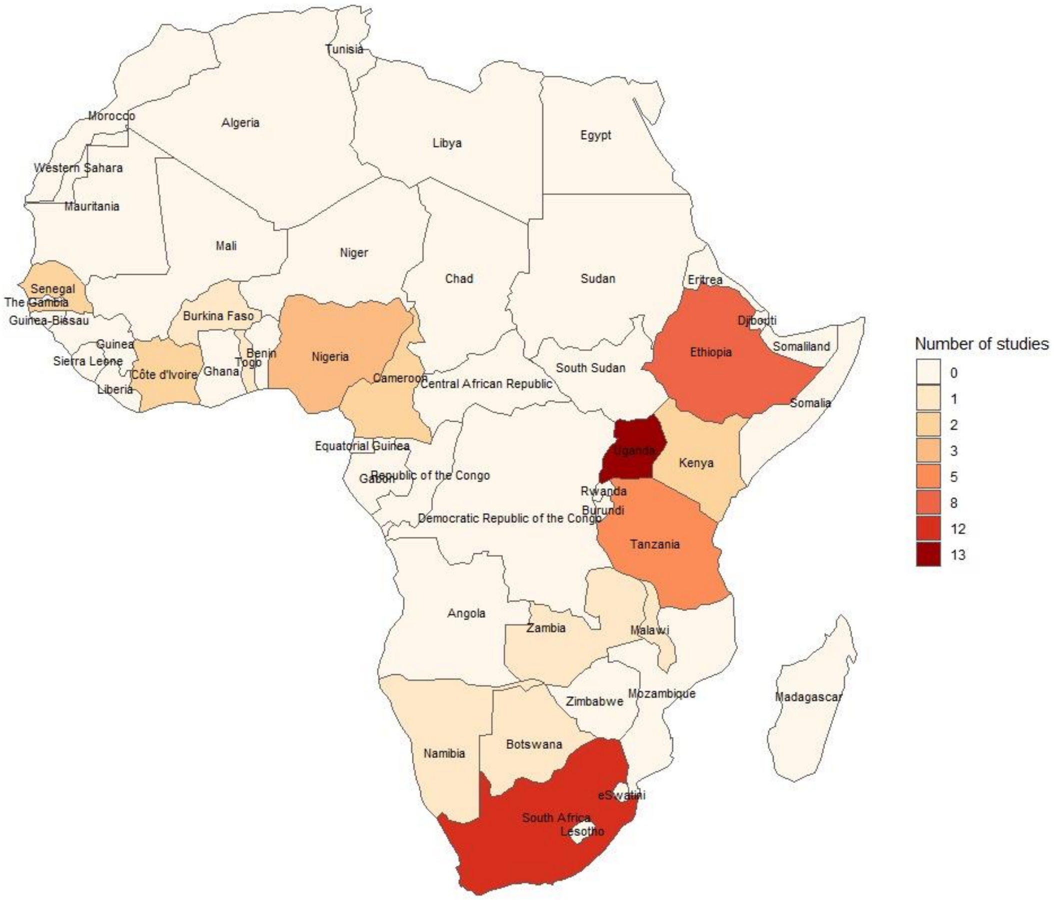

Supplementary Files – Mental health and Wellbeing of older adults living with HIV in sub-Saharan Africa: a systematic review

Supplementary table 1. Search strategy

Example database: PubMed

We adopted a stepwise approach that involves first combining shorter keywords for each part of the PICOS criteria with "OR" and later checking history for the list of searches and combining them with "AND"

The following search terms were included:

- HIV OR HIV-1 OR HIV/AIDS OR HIV infections
- adult OR older adult OR older people OR older individual OR elderly
- Africa OR sub-Saharan Africa OR Africa South of the Sahara
- cognitive impairment OR neurocognitive impairment OR neurological complication OR HIV-associated neurocognitive disorder
- common mental disorder OR depression OR depressive symptoms OR depressive disorder OR anxiety OR anxiety disorder
- quality of life OR health-related quality of life
- grip strength OR hand strength OR frailty

PubMed search – 13/01/2021: ((((((HIV) OR (HIV-1)) OR (HIV/AIDS)) OR (HIV infections)) AND (((((Adult) OR (older adult)) OR (older people)) OR (older individual)) OR (elderly))) AND (((Africa) OR (sub-Saharan Africa)) OR (Africa South of the Sahara))) AND ((((((((((((((cognitive impairment) OR (neurocognitive impairment)) OR (neurological complication)) OR (HIV-associated neurocognitive disorder)) OR (common mental disorder)) OR (depression)) OR (depressive symptoms)) OR (depressive disorder)) OR (anxiety)) OR (anxiety disorder)) OR (quality of life)) OR (health-related quality of life)) OR (grip strength)) OR (hand strength)) OR (frailty))

Number of hits = 2,090

No filters were applied.

We applied the same strategy for CINAHL, PsycINFO, Embase, and Scopus databases.

For peer review only

Supplementary Files – Mental health and Wellbeing of older adults living with HIV in sub-Saharan Africa: a systematic review

Supplementary table 2. Modified Newcastle-Ottawa Risk of Bias Assessment

Author	Representativeness of the sample	Sample size	Comparability between respondents and non-respondents	Outcome ascertainment	Quality of Statistics reporting	Total quality score
**Abadiga, M. (2019).**	**1**	**0**	**1**	**1**	**1**	**4**
Akena, D., et al. (2012).	1	0	0	0	1	2
Asangbeh, S. L., et al. (2016)	0	0	0	1	1	2
Asiimwe, S. B., et al. (2020).	1	1	1	1	1	5
Asrat, B., et al. (2020).	1	0	1	1	1	4
Atashili, J., et al. (2013)	0	0	1	0	1	2
Berhe, H. and A. Bayray (2013)	0	0	0	0	1	1
Bernard, C., et al. (2020).	1	1	0	0	1	3
Bernard, C., et al. (2020).	1	1	0	0	1	3
Bristow, C., et al. (2021).	1	1	1	1	1	5
Cassimjee, N. and P. K. Motswai (2017)	0	0	0	1	1	2
Cholera, R., et al. (2017).	1	0	1	1	1	4
Duko, B., et al. (2018).	1	0	0	1	1	3
Eaton, P., et al. (2020)	1	1	0	1	1	4
Edwards, A., et al. (2020).	1	1	1	0	1	4
Eshetu, D. A., et al. (2015).	0	0	0	0	1	1
Filteau, S., et al. (2017).	1	1	0	1	1	4
Geldsetzer, P., et al. (2019).	1	1	1	0	1	4
Harding, R., et al. (2014).	0	0	0	1	1	2
Joska, J. A., et al. (2019).	1	1	0	0	1	3
Kaharuza, F. M., et al. (2006)	1	0	0	1	1	3
Kalomo, E. N., et al. (2020)	0	1	0	0	1	2
Kellett-Wright, J., et al. (2020)	1	1	0	1	1	4
Kinyanda, E., et al. (2016)	1	1	0	1	1	4
Kobayashi, L. C., et al. (2019)	1	1	1	1	1	5
Maniragaba, F., et al. (2018)	1	0	0	0	1	2
Manne-Goehler, J., et al. (2019).	0	1	0	1	1	3
Motumma, A., et al. (2019).	1	1	1	1	1	5
Moucheraud, C., et al. (2020)	0	0	0	1	1	2
Mugendi, A., et al. (2019).	0	0	0	0	1	1
Mugisha, J., et al. (2013)	1	1	0	1	1	4
Mugisha, J. O., et al. (2016).	1	1	0	1	1	4
Musinguzi, K., et al. (2018).	1	0	0	0	1	2
Negin, J., et al. (2012)	1	1	0	1	1	4
Nyirenda, M., et al. (2012).	1	1	0	1	1	4
Nyirenda, M., et al. (2013).	1	1	0	1	1	4
Nyirenda, M., et al. (2013)	1	1	0	1	1	4
Obimakinde, A. M., et al. (2020).	0	0	1	0	1	2
Olagunju, A. T., et al. (2012).	1	0	0	1	1	3
Olley, B. O., et al. (2017).	0	0	0	1	1	2
Oumar, G., et al. (2020).	0	1	0	0	1	2
Parcesepe, A. M., et al. (2020).	0	0	0	1	1	2
Rohr, J. K., et al. (2020)	1	1	1	0	1	4
Scholten, F., et al. (2011).	1	1	0	1	1	4
Shumba, C., et al. (2013).	1	0	0	1	1	3
Ssonko, M., et al. (2018).	1	1	0	0	1	3
Tesfaw, G., et al. (2016)	1	0	1	1	1	4
Torgersen, J., et al. (2019).	0	1	1	0	1	3
Tsegaw, M., et al. (2017)	0	0	1	0	1	2
Yaya, I., et al. (2019).	1	1	1	0	1	4

For peer review only

Supplementary Files – Mental health and Wellbeing of older adults living with HIV in sub-Saharan Africa: a systematic review

Supplementary table 3: Summary of prevalence estimates and correlates for common mental disorders

Author, publication year & country	Study design	Sample size	Treatment status of PLWH	Cut-off score of the tool used	Information on local tool validation	Prevalence estimates/other results for PLWH50+	Correlates reported
Studies that used the 9-item Patient Health Questionnaire (PHQ-9) to assess depression							
Abadiga, M. (2019); Ethiopia	Cross-sectional	55 PLWH48+; 338 PLWH (18-48 years)	All on ART	≥5	Previously validated in Ethiopia	49.1%	Reported correlates not aggregated by age.
Eshetu, D. A., et al. (2015) ; Ethiopia	Cross-sectional	60 PLWH50+; 356 PLWH (20-49 years)	56.2% on ART	≥5	Sensitivity of 88% and Specificity of 88%	46.7%	Reported correlates not aggregated by age
Torgersen, J., et al. (2019); Botswana	Cross-sectional	125 PLWH50+; 789 PLWH (21-49 years)	ART naïve	≥10	NR	16.0%	NR
Cholera, R., et al. (2017); South Africa	Cross-sectional	28 PLWH50+; 312 PLWH (18-49 years)	ART naïve	≥10	Previously validated against the MINI yielding 78.7% sensitivity and 83.4% specificity in the study population	46.4%	NR
Asangbeh, S. L., et al. (2016) ; Cameroon	Cross-sectional	62 PLWH50+; 140 PLWH (21-49 years)	All on ART	≥10	Validated in a previous study in Cameroon	37.0%	Reported correlates not aggregated by age.
Duko, B., et al. (2018); Ethiopia	Cross-sectional	31 PLWH50+; 352 PLWH (18-54 years)	93.2% on ART	≥11	Previously validated yielding 86% sensitivity and 67% specificity	45.2%	Reported correlates not aggregated by age.
Studies that used the Center for Epidemiological Studies Depression scale (CES-D) to assess depression							
Bernard, C., et al. (2020); Coˆte d'Ivoire & Senegal	Cross-sectional	334 PLWH50+	All on ART	A total score ≥ 17 for men and ≥ 23 for women	Relied on previous validation of the tool from other African countries (Uganda, South Africa and Zambia)	17.9% (95% CI: 13.8-22).	PLWH with severe depressive symptoms were more likely to be **unemployed** (AOR = 2.8; 95% CI: 1.4–5.7); **current or former tobacco smokers** (AOR = 2.6; 95% CI: 1.3–5.4) but **were less likely to be overweight or obese** (AOR = 0.4; 95% CI: 0.2–0.8).
Rohr, J. K., et al. (2020); South Africa	Population survey	1048 PLWH40+; 3512 HIV uninfected older adults ≥40 years	68.6% on ART	≥4 symptoms	NR	8.0% among PLWH40+ versus 10.0% among HIV uninfected older adults ≥40 years.	NR
Moucheraud, C., et al. (2020); Malawi	Cross-sectional	74 PLWH50+ and 60 young PLWH [30-49] years.	All on ART	Mild/major depression symptoms = ≥16 CESD scores; Major depression symptoms = ≥27	NR	Symptoms of mild/major depression: 32.4% among PLWH50+; Major depression symptoms: 10.8% among PLWH50+	Older adults reported less few depressive symptoms (mild depression: AOR 0.23 p = 0.002; major depression: AOR 0.16, p = 0.004)

For peer review only

Supplementary Files – Mental health and Wellbeing of older adults living with HIV in sub-Saharan Africa: a systematic review

Author, publication year & country	Study design	Sample size	Treatment status of PLWH	Cut-off score of the tool used	Information on local tool validation	Prevalence estimates/other results for PLWH50+	Correlates reported
Geldsetzer, P., et al. (2019); South Africa	Population-based survey	1,037 PLWH40+; 4,022 HIV uninfected older adults ≥40 years	63.9% on ART	NR	Not validated previously. The current version underwent intensive cultural adaptation	None of the chronic conditions was significantly associated with depressive symptoms in multivariable regressions.	Older age was positively associated with depression, whereas being married and employment was negatively associated.
Olley, B. O., et al. (2017) ; Nigeria	Cross-sectional	44 PLWH50+; 458 PLWH (18-50 years)	All on ART	≥15	Cronbach's alpha = 0.79. Item-total correlation coefficient ranged between 0.65 and 0.87	20.5%	Not aggregated by age
Kaharuza, F. M., et al. (2006) ; Uganda	Cross-sectional	94 PLWH50+; 923 PLWH (18-50 years)	ART naïve	≥23	Internally consistent (alpha = 0.9)	58.5%	Older age (>50 years) was positively associated with depressive symptoms; AOR 1.93 (95% CI: 1.09, 3.42).
Studies that used the Mini International Neuropsychiatric Interview (MINI) to assess depression							
Asrat, B., et al. (2020); Ethiopia	Cross-sectional	54 PLWH50+; 337 PLWH (18-49 years)	All on ART	NR	Previously adapted in Ethiopia	29.6%	Reported correlates not aggregated by age.
Musinguzi, K., et al. (2018); Uganda	Cross-sectional	21 PLWH50+; 180 PLWH (18-49 years)	ART naïve	NR	NR	14.3%	Reported correlates not aggregated by age.
Kinyanda, E., et al. (2016); Uganda	Cross-sectional	244 PLWH50+; 224 HIV affected but uninfected older adults ≥50 years	9.8% on ART	NR	NR	11.9% among PLWH50+; 6.3% in HIV affected but uninfected older adults ≥50 years	MDD was significantly associated with declining SES, increasing disability scores, decreasing mean grip strength, reported back pain, and not having hypertension.
Mugisha, J. O., et al. (2016) ; Uganda	Cross-sectional	244 PLWH50+; 227 HIV uninfected older adults ≥50 years	90.6% on ART	NR	Locally adapted	9.5% among PLWH50+; 5.8% in the uninfected older adults ≥50 years	NR
Akena, D., et al. (2012); Uganda	Cross-sectional	51 PLWH50+; 317 PLWH (18-49 years)	NR	≥5 of the 9 DSM-IV-TR symptoms for major depression	Not locally validated	11.8%	Reported correlates not aggregated by age.

For peer review only

Supplementary Files – Mental health and Wellbeing of older adults living with HIV in sub-Saharan Africa: a systematic review

Author, publication year & country	Study design	Sample size	Treatment status of PLWH	Cut-off score of the tool used	Information on local tool validation	Prevalence estimates/other results for PLWH50+	Correlates reported
Studies that used the geriatric depression scale to assess depression							
Kalomo, E. N., et al. (2020); Namibia	Cross-sectional	147 PLWH50+	All on ART	≥3 symptoms	Cronbach alpha of 0.73 in the current study	46.1%	There was a positive association between HIV stigma (negative self-perceptions) and depression and a negative relationship between resilience and depression. Also, being female and higher education were protective factors and disclosure of HIV status was a risk factor
Eaton, P., et al. (2020); Tanzania	Cross-sectional	253 PLWH50+	94.8% on ART	≥5 symptoms	NR	17.4%	Not reported
Studies that used the Composite International Diagnostic Interview (CIDI) to assess depression							
Nyirenda, M., et al. (2013); South Africa	Cross-sectional	203 PLWH50+; 219 HIV uninfected older adults ≥50 years	23.7% on ART	ICD 10-DCR criteria	Locally adapted	14.8% among PLWH50+; 30.1 % in the uninfected older adults ≥50 years	Being female (aOR 3.04, 95% CI 1.73–5.36), receiving a government grant (aOR 0.34, 95% CI 0.15–0.75), urban residency (aOR 1.86, 95% CI 1.16–2.96) and adult caregiving (aOR 2.37, 95% CI 1.37–4.12) were significantly associated with any depressive episode
Negin, J., et al. (2012); South Africa	Cross-sectional	142 PLWH50+; 2,722 HIV uninfected older adults ≥50 years	NR	NR	Locally adapted	6.0% among PLWH50+; 4.8% in the uninfected older adults ≥50 years	NR
Studies that used other methods to assess depression							
Kellett-Wright, J., et al. (2020); Tanzania	Cross-sectional	235 PLWH50+	95.5% on ART	DSM-IV; cut-off NR	NR	16.6%	NR
Motumma, A., et al. (2019); Ethiopia	Cross-sectional	122 PLWH40+; 298 HIV infected young adults (18-40) years	All on ART	A score of ≥7 of Self-Reporting Questionnaire (SRQ-20)	Previously validated in Ethiopia, with high sensitivity (85.7%) and specificity (75.6%).	23.0%	Correlates not aggregated by age
Manne-Goehler, J., et al. (2019); Uganda	Cross-sectional	154 PLWH40+; 142 HIV uninfected older adults ≥40 years	All on ART	Mean score of ≥1.75 on the 15-Item Hopkins Checklist (HSCL)	Previously validated in the same population	21.4% among PLWH40+; 33.8% in uninfected older adults ≥40 years	PLWH on ART and those falling in the highest wealth quartile had a lower prevalence of probable depression. In comparison, women had a significantly higher prevalence of depression than men. Education and age were not significantly associated with depression.

For peer review only

Supplementary Files – Mental health and Wellbeing of older adults living with HIV in sub-Saharan Africa: a systematic review

Author, publication year & country	Study design	Sample size	Treatment status of PLWH	Cut-off score of the tool used	Information on local tool validation	Prevalence estimates/other results for PLWH50+	Correlates reported
Tesfaw, G., et al. (2016); Ethiopia	Cross-sectional	59 PLWH50+; 358 PLWH (19-49 years)	All on ART	A cut-off ≥8 on the 7-item Hospital Anxiety and Depression Scale	Previously validated	39.0%	Not aggregated by age
Shumba, C., et al. (2013); Uganda	Cross-sectional	91 PLWH50+; 584 PLWH (19-49 years)	All on ART	A cut-off of ≥1 on a locally adapted 5-item symptom questionnaire	Cronbach's alpha of 0.87	53.9%	NR
Berhe, H. and A. Bayray (2013); Ethiopia	Cross-sectional	19 PLWH54+; 250 PLWH (18-54 years)	NR	A score of ≥8 on the 21-Item Hamilton Depression Scale	NR	57.9%	Reported correlates not aggregated by age.
Studies reporting anxiety							
Obimakinde, A. M., et al. (2020) ; Nigeria	Cross-sectional	62 PLWH60+; 162 HIV uninfected older adults ≥60 years	All on ART	Extracted from participants clinical notes	NR	3.2% among PLWH50+; 3.2% in HIV uninfected older adults	NR
Olagunju, A. T., et al. (2012) ; Nigeria	Cross-sectional	24 PLWH50+; 276 PLWH (18-50 years)	All on ART	Clinical Assessment in Neuropsychiatry (SCAN);	NR	20.8%	Not aggregated by age

Notes: ART – *Antiretroviral treatment*; **AOR** – *Adjusted odds ratio*; **CES-D** – *Center for Epidemiologic Studies Depression Scale*; **CI** – *Confidence interval*; **CIDI** – *Composite International Diagnostic Interview*; **DSM-IV**– *Diagnostic and Statistical Manual of Mental Disorders, fourth edition;* **HSCL** – *Hopkins Symptoms Checklist*; **MDD** – *Major depressive disorder*; **MINI** – *Mini International Neuropsychiatric Interview*; **NR** – *Not reported*; **PLWH** – *People living with HIV*; **PLWH50+** – *People living with HIV ≥50 years old*; **SCAN** – *Clinical Assessment in Neuropsychiatry*

For peer review only

Research Checklist– Mental health and Wellbeing of older adults living with HIV in sub-Saharan Africa: a systematic review

PRISMA 2020 Checklist

Section and Topic	Item #	Checklist item	Location where item is reported
**TITLE**			
Title	1	Identify the report as a systematic review.	Page 1
**ABSTRACT**			
Abstract	2	See the PRISMA 2020 for Abstracts checklist.	Page 2
**INTRODUCTION**			
Rationale	3	Describe the rationale for the review in the context of existing knowledge.	Page 3
Objectives	4	Provide an explicit statement of the objective(s) or question(s) the review addresses.	Page 4
**METHODS**			
Eligibility criteria	5	Specify the inclusion and exclusion criteria for the review and how studies were grouped for the syntheses.	Page 4
Information sources	6	Specify all databases, registers, websites, organisations, reference lists and other sources searched or consulted to identify studies. Specify the date when each source was last searched or consulted.	Page 4
Search strategy	7	Present the full search strategies for all databases, registers and websites, including any filters and limits used.	Page 4 and supplementary table 1
Selection process	8	Specify the methods used to decide whether a study met the inclusion criteria of the review, including how many reviewers screened each record and each report retrieved, whether they worked independently, and if applicable, details of automation tools used in the process.	Page 4
Data collection process	9	Specify the methods used to collect data from reports, including how many reviewers collected data from each report, whether they worked independently, any processes for obtaining or confirming data from study investigators, and if applicable, details of automation tools used in the process.	Page 4
Data items	10a	List and define all outcomes for which data were sought. Specify whether all results that were compatible with each outcome domain in each study were sought (e.g. for all measures, time points, analyses), and if not, the methods used to decide which results to collect.	Page 4 and 5
	10b	List and define all other variables for which data were sought (e.g. participant and intervention characteristics, funding sources). Describe any assumptions made about any missing or unclear information.	N/A
Study risk of bias assessment	11	Specify the methods used to assess risk of bias in the included studies, including details of the tool(s) used, how many reviewers assessed each study and whether they worked independently, and if applicable, details of automation tools used in the process.	Page 4 and 5
Effect measures	12	Specify for each outcome the effect measure(s) (e.g. risk ratio, mean difference) used in the synthesis or presentation of results.	Page 4
Synthesis methods	13a	Describe the processes used to decide which studies were eligible for each synthesis (e.g. tabulating the study intervention characteristics and comparing against the planned groups for each synthesis (item #5)).	N/A
	13b	Describe any methods required to prepare the data for presentation or synthesis, such as handling of missing summary	N/A

For peer review only

Research Checklist– Mental health and Wellbeing of older adults living with HIV in sub-Saharan Africa: a systematic review

Section and Topic	Item #	Checklist item	Location where item is reported
		statistics, or data conversions.	
	13c	Describe any methods used to tabulate or visually display results of individual studies and syntheses.	Page 5
	13d	Describe any methods used to synthesize results and provide a rationale for the choice(s). If meta-analysis was performed, describe the model(s), method(s) to identify the presence and extent of statistical heterogeneity, and software package(s) used.	Page 5
	13e	Describe any methods used to explore possible causes of heterogeneity among study results (e.g. subgroup analysis, meta-regression).	N/A
	13f	Describe any sensitivity analyses conducted to assess robustness of the synthesized results.	N/A
Reporting bias assessment	14	Describe any methods used to assess risk of bias due to missing results in a synthesis (arising from reporting biases).	N/A
Certainty assessment	15	Describe any methods used to assess certainty (or confidence) in the body of evidence for an outcome.	N/A
**RESULTS**			
Study selection	16a	Describe the results of the search and selection process, from the number of records identified in the search to the number of studies included in the review, ideally using a flow diagram.	Page 5 and 6
	16b	Cite studies that might appear to meet the inclusion criteria, but which were excluded, and explain why they were excluded.	N/A
Study characteristics	17	Cite each included study and present its characteristics.	Page 20 to 28
Risk of bias in studies	18	Present assessments of risk of bias for each included study.	Supplementary table 2
Results of individual studies	19	For all outcomes, present, for each study: (a) summary statistics for each group (where appropriate) and (b) an effect estimate and its precision (e.g. confidence/credible interval), ideally using structured tables or plots.	Pages 20 to 28
Results of syntheses	20a	For each synthesis, briefly summarise the characteristics and risk of bias among contributing studies.	Supplementary table 2
	20b	Present results of all statistical syntheses conducted. If meta-analysis was done, present for each the summary estimate and its precision (e.g. confidence/credible interval) and measures of statistical heterogeneity. If comparing groups, describe the direction of the effect.	N/A
	20c	Present results of all investigations of possible causes of heterogeneity among study results.	N/A
	20d	Present results of all sensitivity analyses conducted to assess the robustness of the synthesized results.	N/A
Reporting biases	21	Present assessments of risk of bias due to missing results (arising from reporting biases) for each synthesis assessed.	N/A
Certainty of evidence	22	Present assessments of certainty (or confidence) in the body of evidence for each outcome assessed.	N/A
**DISCUSSION**			

For peer review only

Research Checklist– Mental health and Wellbeing of older adults living with HIV in sub-Saharan Africa: a systematic review

Section and Topic	Item #	Checklist item	Location where item is reported
Discussion	23a	Provide a general interpretation of the results in the context of other evidence.	Pages 10-14
	23b	Discuss any limitations of the evidence included in the review.	Page 13-14
	23c	Discuss any limitations of the review processes used.	Page 13-14
	23d	Discuss implications of the results for practice, policy, and future research.	Page 14
**OTHER INFORMATION**			
Registration and protocol	24a	Provide registration information for the review, including register name and registration number, or state that the review was not registered.	Page 2 and 4
	24b	Indicate where the review protocol can be accessed, or state that a protocol was not prepared.	Page 4
	24c	Describe and explain any amendments to information provided at registration or in the protocol.	N/A
Support	25	Describe sources of financial or non-financial support for the review, and the role of the funders or sponsors in the review.	Page 14
Competing interests	26	Declare any competing interests of review authors.	Page 14
Availability of data, code and other materials	27	Report which of the following are publicly available and where they can be found: template data collection forms; data extracted from included studies; data used for all analyses; analytic code; any other materials used in the review.	Page 14

For peer review only

BMJ Open

Mental health and wellbeing of older adults living with HIV in sub-Saharan Africa: a systematic review

Journal:	*BMJ Open*
Manuscript ID	bmjopen-2021-052810.R2
Article Type:	Original research
Date Submitted by the Author:	03-Sep-2021
Complete List of Authors:	Mwangala, Patrick; KEMRI-Wellcome Trust Research Programme, Neurosciences Department; University of the Witwatersrand School of Public Health,<br>Mabrouk, Adam; KEMRI-Wellcome Trust Research Programme, Neurosciences Department<br>Wagner, Ryan; University of the Witwatersrand, MRC/Wits Rural Public Health and Health Transitions Research Unit (Agincourt), School of Public Health, Faculty of Health Sciences<br>Newton, Charles; KEMRI-Wellcome Trust Research Programme, Neurosciences Department; University of Oxford Department of Psychiatry<br>Abubakar, Amina A.; KEMRI-Wellcome Trust Research Programme, Neurosciences Department; Institute for Human Development, Aga Khan University
**Primary Subject Heading**:	HIV/AIDS
Secondary Subject Heading:	Mental health, Geriatric medicine
Keywords:	HIV & AIDS < INFECTIOUS DISEASES, PSYCHIATRY, PUBLIC HEALTH

SCHOLARONE™
Manuscripts

I, the Submitting Author has the right to grant and does grant on behalf of all authors of the Work (as defined in the below author licence), an exclusive licence and/or a non-exclusive licence for contributions from authors who are: i) UK Crown employees; ii) where BMJ has agreed a CC-BY licence shall apply, and/or iii) in accordance with the terms applicable for US Federal Government officers or employees acting as part of their official duties; on a worldwide, perpetual, irrevocable, royalty-free basis to BMJ Publishing Group Ltd ("BMJ") its licensees and where the relevant Journal is co-owned by BMJ to the co-owners of the Journal, to publish the Work in this journal and any other BMJ products and to exploit all rights, as set out in our licence.

The Submitting Author accepts and understands that any supply made under these terms is made by BMJ to the Submitting Author unless you are acting as an employee on behalf of your employer or a postgraduate student of an affiliated institution which is paying any applicable article publishing charge ("APC") for Open Access articles. Where the Submitting Author wishes to make the Work available on an Open Access basis (and intends to pay the relevant APC), the terms of reuse of such Open Access shall be governed by a Creative Commons licence – details of these licences and which Creative Commons licence will apply to this Work are set out in our licence referred to above.

Other than as permitted in any relevant BMJ Author's Self Archiving Policies, I confirm this Work has not been accepted for publication elsewhere, is not being considered for publication elsewhere and does not duplicate material already published. I confirm all authors consent to publication of this Work and authorise the granting of this licence.

For peer review only

Mental health and wellbeing of older adults living with HIV in sub-Saharan Africa: a systematic review

*Patrick N Mwangala[1,2], Adam Mabrouk[1], Ryan G Wagner[3], Charles R Newton[1, 4,] Amina Abubakar[1, 4 5,6]

Author Affiliations:

[1]Centre for Geographic Medicine Research Coast, Kenya Medical Research Institute (KEMRI), P.O Box 230-80108, Kilifi, Kenya

[2]School of Public Health, University of the Witwatersrand, 27 St Andrews Road, Parktown 2193, South Africa

[3]MRC/Wits Rural Public Health and Health Transitions Research Unit (Agincourt), University of the Witwatersrand, Faculty of Health Sciences, Johannesburg, South Africa

[4]Department of Psychiatry, University of Oxford, Warneford Hospital, Warneford Ln, Oxford OX3 7JX, United Kingdom

[5]Department of Public Health, Pwani University, P.O. BOX 195-80108, Kilifi, Kenya

[6]Institute for Human Development, Aga Khan University, P.O. BOX 30270-00100, Nairobi, Kenya

*Corresponding author: Patrick N Mwangala; Email: pmwangala27@gmail.com; Tel: +254 708 759457

For peer review only

For peer review only - http://bmjopen.bmj.com/site/about/guidelines.xhtml

ABSTRACT

Objective: In this systematic review, we aimed to summarise the empirical evidence on common mental disorders (CMDs), cognitive impairment, frailty and health-related quality of life (HRQoL) among people infected with human immunodeficiency virus (HIV) aged ≥50 years (PLWH50+) residing in sub-Saharan Africa (SSA). Specifically, we document the prevalence and correlates of these outcomes.

Design, data sources and eligibility criteria: The following online databases were systematically searched: PubMed, CINAHL, PsycINFO, Embase and Scopus up to Jan 2021. English-language publications on depression, anxiety, cognitive function, frailty, and quality of life among PLWH50+ residing in SSA were included.

Data extraction and synthesis: We extracted information, including study characteristics, and main findings. These were tabulated, and a narrative synthesis approach was adopted, given the substantial heterogeneity among included studies.

Results: A total of 50 studies from fifteen SSA countries met the inclusion criteria. About two-thirds of these studies emanated from Ethiopia, Uganda, and South Africa. Studies regarding depression predominated (*n=26),* followed by cognitive impairment (*n=13*). Overall, PLWH50+ exhibited varying prevalence of depression (6% to 59%), cognitive impairments (4% to 61%) and frailty (3% to 15%). The correlates of CMDs, cognitive impairment, frailty and HRQoL were investigated, but those reported were sociodemographic variables, many of which were inconsistent.

Conclusions: This review documented an increasing number of published studies on HIV and aging from SSA. However, the current evidence on the mental and wellbeing outcomes in PLWH50+ is inadequate to characterise the public health dimension of these impairments in SSA, because of heterogeneous findings, few well-designed studies and substantial methodological limitations in many of the available studies. Future work should have sufficiently large samples of PLWH50+, engage appropriate comparison groups, harmonise the measurement of these outcomes using a standardised methodology to generate more robust prevalence estimates and confirm predictors.

PROSPERO registration number: CRD42020145791

Strengths and Limitations of this study

- This is the first comprehensive review of common mental disorders, cognitive impairment, frailty and quality of life among older adults living with HIV in SSA, synthesising the current evidence regarding the prevalence and correlates of these outcomes.
- Our expanded search of five online databases and snowballing techniques minimised the risk of missing relevant studies.
- Meta-analysis was not conducted due to extensive heterogeneity across the studies in methods and presentation of results.
- We observed substantial methodological limitations in many of the included studies, e.g. small sample sizes, making it difficult to characterise the real public health dimension of these impairments in the region.

For peer review only - http://bmjopen.bmj.com/site/about/guidelines.xhtml

1.0 BACKGROUND

All societies globally are experiencing an aging population – some are in its early stages, and some are more advanced [1]. Likewise, the proportion of people living with HIV (PLWH) aged ≥50 years (considered as older adults within the HIV literature) is growing rapidly across the globe [2], demanding an increase in research, policy and practice to address the complex needs of this vulnerable group [3]. In 2016 there were around 6 million PLWH50+ globally, representing 16% of the entire adult HIV population [2]. This proportion was projected to reach 21% by the end of 2020, with Southern and Eastern Africa containing the vast majority of PLWH50+ [2]. The increase in older PLWH is mainly driven by two factors: effective antiretroviral therapy (ART) and new HIV infections among the elderly [4].

This rapidly growing segment of PLWH50+ yields new challenges. Several HIV and aging cohorts (mostly from high-income countries – HICs) are pointing out an elevated burden of physical conditions (e.g. cardiometabolic diseases), mental morbidities (e.g. depression, cognitive disorders), psychosocial challenges (e.g. stigma, loneliness), and geriatric syndromes (e.g. frailty) among PLWH ≥50 years (PLWH50+) compared to their uninfected counterparts [4-6]. Unfortunately, the mechanisms for the heightened risk of poor outcomes among PLWH50+ are not fully understood but span a host of disease-related factors, legacy of the early years of the HIV epidemic, and host vulnerability factors [4]. Frailty, common mental disorders (CMDs) such as depression and anxiety, and cognitive impairments are of particular concern in this population [4-6]. Frailty is an important emerging indicator of vulnerability that is increasingly being evaluated among individuals aging with HIV [7]. It describes a state of physiologic vulnerability that, in the presence of internal or external stressors, puts a person at an elevated risk of adverse clinical outcomes, such as hospitalisation, functional disability, and mortality [8, 9]. Overall, mental morbidities and frailty are understudied in the literature despite their elevated burden and potential adverse impacts on HIV treatment and health-related quality of life (HRQoL).

Despite the apparent need to sharpen the focus on the needs of the growing cohort of PLWH50+, this population is being neglected in HIV care and prevention efforts, especially in low- and middle-income settings of sub-Saharan Africa (SSA). In SSA, this concern is not just based on the close association between aging and physical, cognitive, mental impairments, but also on the recognition that older adults are not considered to be a priority for policy issues. They are marginalised and increasingly vulnerable to greater poverty [10, 11]. Thus, the burden and determinants of physical and mental impairments in these adults in SSA, the region most affected by HIV globally, are largely undocumented [12].

The course and implication of physical and mental morbidities among PLWH50+ are well described in HICs, but it is an emergent research area in SSA. However, since 2010, the SSA region has witnessed a rise in interest in HIV and aging outcomes, evidenced by the establishment of cohort studies of older people in different parts of Africa [13-15]. This is encouraging since data from HICs may not be readily generalized to SSA where differences in, e.g. genetics, social-environmental milieu, formal and informal support systems will almost certainly alter the risk profile and morbidity characteristics among PLWH50+. Therefore, the current review aims to summarise the existing empirical evidence on common mental disorders (CMDs), cognitive impairment, frailty, and health-related quality of life (HRQoL) among PLWH50+ in SSA by addressing the following specific objectives.

For peer review only - http://bmjopen.bmj.com/site/about/guidelines.xhtml

a) Establish the prevalence of CMDs, cognitive impairment and frailty among PLWH50+ in SSA.

b) Identify the correlates of CMDs, cognitive impairment, frailty and HRQoL among PLWH50+ in SSA.

2.0 METHODS

We followed the Preferred Reporting Items for Systematic Reviews and Meta-analyses (PRISMA) guidelines to report the study [16]. The review protocol was prospectively registered in PROSPERO (Registration number: CRD42020145791).

2.1. Search strategy. Five online databases (PubMed, CINAHL, PsycINFO, Embase, and Scopus) were searched for publications. The last search was conducted on Jan 4th, 2021. The following search terms were used: (HIV OR HIV-1 OR HIV/AIDS OR HIV infections) AND (adult OR older adult OR older people OR older individual OR elderly) AND (Africa OR sub-Saharan Africa OR Africa South of the Sahara) AND (cognitive impairment OR neurocognitive impairment OR neurological complication OR HIV-associated neurocognitive disorder OR common mental disorder OR depression OR depressive symptoms OR depressive disorder OR anxiety OR anxiety disorder OR quality of life OR health-related quality of life OR grip strength OR hand strength OR frailty). We also searched through the reference lists of included articles to access additional articles. Details of the full search strategy are provided in Supplementary file 1.

Studies were considered eligible if they: (i) involved PLWH50+ or had participants whose mean/median age was ≥ 50 years or aggregated their outcomes of interest by age (including the ≥ 50 age category); (ii) were conducted within SSA; (iii) were empirical and published in a peer-reviewed journal; (iv) reported any of our outcomes of interest (CMDs, cognitive impairment, frailty or HRQoL). We excluded studies that did not aggregate the outcomes of interest by HIV status. We also excluded studies that were published in non-English languages. Two of the authors (PNM and AM) independently screened the titles, abstracts and full articles for eligibility and reached consensus.

2.2. Data extraction and quality assessment. The two authors (PNM and AM) independently extracted the following study characteristics using a similarly designed extraction sheet: (i) first author; (ii) country and the year of publication; (iii) sample description; (iv) how the outcome of interest was measured; (v) results reported; (vi) key findings on the outcome of interest. For studies exclusively carried out among PLWH50+, we computed or extracted the reported percentages of the relevant outcomes. Similarly, for studies with middle-aged participants but whose mean/median age was ≥50 years, we also computed or extracted the reported percentages for the relevant outcomes for the whole sample. For the studies with middle-aged participants (but whose mean/median age was not at least 50 years), we computed percentages for participants who fell within the ≥ 50 category, which was usually provided in the papers. In some studies, it was impossible to calculate these percentages. Hence, the occurrence of a specific outcome was reported in the original effect measure, e.g. odds ratio, median or mean. Any disagreement between the reviewers during data abstraction was also resolved by consensus.

We evaluated the quality of included studies using the modified version of the Newcastle-Ottawa Scale (NOS) [17]. The scale uses five parameters [*sample size, sample*

For peer review only - http://bmjopen.bmj.com/site/about/guidelines.xhtml

representativeness, comparability between respondents and non-respondents, ascertainment of the outcome of interest, and quality of descriptive statistics reporting] to obtain a total score ranging from 0 – 5. A score ≥3 is considered a low risk of bias, while a score of <3 is regarded as a high risk of bias.

2.3. Data Synthesis and Analysis. A narrative synthesis approach was used to compare studies and describe the state of evidence on all reported outcomes; by describing the effect estimates, for instance, proportions, means with standard deviations, and median with their interquartile ranges. Where applicable, ranges were used to summarise the outcomes of interest reported. A meta-analysis could not be done due to a limited number of studies and substantial heterogeneity among studies.

2.4. Patient and Public Involvement Statement. The accompanying manuscript is part of broader doctoral research work for the first author. During the conceptual phase of this work, community engagement through meetings with different stakeholders was carried out to contextualise the issues of aging and HIV at the coast of Kenya (e.g. in terms of priorities and preferences). These stakeholders included the Kilifi and Mombasa counties' departments of Health, sub-county health management teams, and hospital management teams of various health facilities at the study setting. The research team received and incorporated useful feedback from these meetings towards the design and implementation of the project. It also led to further elaborations regarding ongoing community engagement during and after the study, including disseminating any work arising from this project using these forums and others such as publications, hospital departmental meetings, patient groups and conferences as an essential way to discuss the implications of the findings. For this systematic review, patients were not involved in the conduct of the study. However, the results will be disseminated to the relevant patient groups within the HIV clinics through feedback meetings.

3.0 RESULTS

3.1. Characteristics of included studies

We identified 8,741 articles from the 5 online databases and an additional 7 citations from snowballing. Of these, 2,693 were duplicates. Hence, we screened 6,055 titles and abstracts for initial eligibility, out of which 575 articles were identified. Full articles were obtained for these citations, of which 50 met the eligibility criteria (Figure 1). The eligible studies were conducted between 2006 and 2021 among 15 SSA countries of Uganda (n=13), South Africa (n=12), Ethiopia (n=8), Tanzania (n=5), Nigeria (n=3), Cameroon (n=2), Côte d'Ivoire (n=2), Senegal (n=2), Kenya (n=2), Botswana (n=1), Zambia (n=1), Malawi (n=1), Namibia (n=1), Togo (n=1) and Burkina Faso (n=1). However, some of these studies were conducted in multiple countries. Figure 2 shows the geographical distribution of these studies across the SSA region. Virtually all the included studies were cross-sectional, except one that presented data from a randomised trial [18]. Eighteen studies (36%) analysed baseline or follow up data of different cohort studies.

About two-thirds of the included studies *(n=35)* recruited their HIV infected samples from clinical settings [18-52]. The rest obtained their samples from the general population through household surveys and community samples. Additionally, many of the studies included samples of PLWH50+ lower than 100 (*n=22*). Overall, the samples of PLWH50+ per study

For peer review only - http://bmjopen.bmj.com/site/about/guidelines.xhtml

ranged from 19 to 1,048 [36, 53], while those for HIV uninfected older adults ranged from 17 to 4,022 [22, 54].

Common HIV disease markers such as the duration of HIV infection, current/nadir CD4+ T cell count, duration of ART use, viral suppression level among PLWH50+ were reported in a few studies. However, there was extensive heterogeneity in how the information was reported and the level of detail given. In 42% of the included studies, all PLWH50+ were on ART. Six other studies included only ART naïve PLWH50+. Among ART users, viral suppression was reported in 5 studies where it was documented to range from 73% - 90%. Nadir CD4 + T cell count among PLWH50+ was reported in 4 studies where most of the participants (about 60%) had a count lower than 200. The duration of HIV infection among PLWH50+ ranged from 3.5 to 12 years (among the few studies that reported this variable).

Only eight studies examined the health outcomes of interest using an exclusively HIV infected older sample [25, 33, 40, 45-49]. Eighteen other studies used HIV uninfected older adults as comparison groups, while the rest included HIV infected young adults (the ages varied extensively).

Insert Figure 1 – ***Identification of eligible studies*** – here

Insert Figure 2 – ***Geographical distribution of the included studies within SSA*** – here

3.2. Depression and anxiety.

Depression was reported in 25 studies, while anxiety was reported in 2 studies. Notably, depression was assessed using multiple scales with diverse scoring systems. Six of the studies used the 9-item Patient Health Questionnaire – PHQ-9, while a similar number used the Center for Epidemiological Studies Depression scale – CES-D. Five other studies used the MINI International Neuropsychiatric Interview – MINI, while the rest utilized a host of different scales. On the other hand, anxiety was assessed using the Schedule for Clinical Assessment in Psychiatry (SCAN) and from clinical records [30, 39]. Nine of the depression studies [19, 29, 44, 49, 51, 55-58] reported major depressive disorders (MDD), while the remaining sixteen studies reported the prevalence of depressive symptoms. To synthesise and report the available evidence, depression (whenever used in this review) captures both the depressive symptoms (derived from brief screening measures) and Major Depressive Disorder (based on structured diagnostic interviews, e.g. the MINI by specialised professionals or trained non-specialised workers). More information on this outcome is presented in the Supplementary Table 1.

Prevalence of depression and anxiety

Overall, the prevalence of depression ranged from 6% to 59%. We found no clear pattern of overreporting or underreporting of depression prevalence in the included studies, considering the different cut-offs used. For instance, in studies using the PHQ-9, the prevalence of depression ranged from 47% to 49% (with a cut-off of ≥5) and 16% to 45% when using a cut-off of ≥10. We also noted a similar observation when we took the ART status of PLWH into

For peer review only - http://bmjopen.bmj.com/site/about/guidelines.xhtml

account. Specifically, the prevalence of depression ranged from 8% to 59% among studies whose participants were entirely on ART compared to 6% to 54% among studies with mixed or naïve PLWH. However, studies that used structured interviews such as the MINI, CIDI and DSM-IV to assess for depression were noted to frequently report lower prevalence of depression among PLWH50+ (ranging from 6% to 30%) [44, 57] compared to studies that used brief screening scales such as the PHQ-9 (prevalence ranging from 8% to 59%) [27, 59].

Of note, eight studies documented higher prevalence of depression among PLWH50+ than their comparison groups [20, 23, 27, 36, 37, 43, 55, 56]. On the contrary, 11 studies reported a lower prevalence of depression among PLWH50+ than their comparison groups [19, 24, 28, 29, 31, 32, 44, 50, 51, 58, 59]. In comparison, three other studies reported minimal or no differences in depression between PLWH50+ and their comparison groups [34, 42, 57]. In one population-based study conducted in rural South Africa, neither diagnosis nor treatment of HIV was significantly associated with depression when compared with being uninfected [54].

Anxiety was reported in two studies [30, 39]. In one of them [39], PLWH50+ on ART presented with the same proportion of anxiety symptoms with HIV uninfected older adults at 3%. In the other study [30], the prevalence of anxiety disorders among PLWH50+ on ART was 21% compared to 22% in HIV infected young adults (18 – 50 years).

Reported correlates of depression

Correlates of depression among PLWH50+ were reported only in 8 studies [27, 28, 46, 48, 51, 54, 55, 58]. Among sociodemographic variables, older age [27, 54], declining socioeconomic status [55], being unemployed [46], being female [28, 58], HIV status disclosure [48], and urban residency [58] were associated with an elevated risk of depression while being married, employed (full time or part-time work) [54], old age [51], higher educational attainment [48], and belonging to the highest wealth quintile [28] were associated with reduced levels of depression. However, in some studies, household wealth quintile, education levels [51], and age [25] were not significant correlates of depression. In other studies, being a current/former smoker [46], increasing disability scores, decreasing mean handgrip strength, reported back pain, not having hypertension [55], HIV stigma [48], and caregiving (for adult children) [58] were significantly associated with elevated levels of depression. On the other hand, receiving a government grant [58] and resilience [48] were significantly associated with reduced levels of depression. Details of this data are presented in Supplementary Table 1.

3.3. Cognitive and neurological functioning

These outcomes were reported by 13 studies, as highlighted in Table 1. Of these, five studies quantified the prevalence of HIV-associated neurocognitive disorders (HAND) using different measures [25, 35, 38, 40, 49], two studies reported findings on dementia [21, 60] while 5 studies documented findings on cognitive function/impairment [22, 33, 51, 53, 61]. The remaining study reported the proportion of HIV-associated peripheral neuropathy among PLWH50+ [39].

For peer review only - http://bmjopen.bmj.com/site/about/guidelines.xhtml

Prevalence estimates of reported outcomes

The prevalence of symptomatic HAND among PLWH50+ on ART ranged from 19% [38] to 61% [35]. Two of the HAND studies documented elevated proportions of symptomatic HAND among PLWH50+ on ART compared to their younger counterparts [35, 38]. In rural South Africa, Joska and colleagues examined the prevalence of dementia and its associations in a population survey of 1,150 rural elderly participants. They found no significant differences in the prevalence of dementia between PLWH50+ (86% on ART) and HIV uninfected counterparts 18% vs 11%, respectively [60]. In a different study in rural South Africa, a baseline analysis of the Health and Aging in Africa – A Longitudinal Study of an INDEPTH Community (HAALSI), HIV infected older adults (64% on ART) registered a lower prevalence of cognitive impairment (4%) than their uninfected peers (9%) [53]. In a separate analysis of the same baseline data of the HAALSI cohort [61], HIV infected older adults scored on average 0.06 (95% confidence interval (CI) 0.01–0.12) standard deviation (SD) units higher on a conventional cognitive function measure and 0.02 (95%CI − 0.07 to 0.04) SD units lower when using the Oxford Cognitive Screen-Plus (OCS-Plus) measure than their HIV-negative counterparts.

Correlates of cognitive function/impairment

The correlates of cognitive function/impairment were reported in 7 studies. The key predictors of reduced cognitive function were mainly sociodemographic: older age (≥50 years) [21, 40, 53, 60], living alone [25], being illiterate or having low formal education level [25, 53], being older at the time of HIV diagnosis [25], being female, unmarried, and having low socioeconomic status [53] were associated with a higher risk of impairment. However, in some studies, the effects of age, education, and marital status were not significant [25, 60]. Other correlates of cognitive impairment included the presence of depressive symptoms [60], a history of cardiovascular conditions [53], and a history of falls in the past 12 months [33]. HIV specific factors (such as nadir CD4 count, ART use) [25, 61] and vascular risk factors (such as smoking status, body mass index and blood pressure) [25] were not associated with cognitive impairment.

Insert Table 1– ***Summary of prevalence estimates and correlates for cognitive impairment/neurological impairment*** – here

For peer review only - http://bmjopen.bmj.com/site/about/guidelines.xhtml

3.4. Frailty/grip strength.

Prevalence estimates

Frailty was reported in three studies [33, 47, 62]. In the first study, a hospital-based cross-sectional study of 145 PLWH50+ on ART in Tanzania, frailty assessment was completed using 3 measures (the Fried frailty phenotype – FFP, the Clinical Frailty Scale – CFS, and Brief Frailty Instrument for Tanzania – B-FIT 2) [47]. In this study, the authors reported low levels of frailty: 3% (when using the FFP method) and 1% (when using the CFS and BFIT-2 methods). The second study documented a similar prevalence of frailty among 292 PLWH50+ *(ART status not reported)* and 322 HIV uninfected peers in rural South Africa (15% vs 18% respectively), using a modified version of the FFP [62]. In the third study, increasing frailty scores were significantly associated with increasing polypharmacy levels in a sample of 411 PLWH50+ on ART in Uganda [33]. Five other studies [18, 45, 57, 63, 64] reported mean handgrip strength as an indicator of frailty, as shown in Table 2.

Reported correlates of frailty

The correlates of frailty were reported in two studies. Being female, unmarried [47] and older [47, 62] were significantly associated with worse frailty scores, and there were no associations between inflammatory biomarkers with frailty [62]. Factors associated with mean handgrip strength were reported in four of the studies. In one, HIV status was significantly associated with weaker handgrip strength [57]. In another study, being female and divorced/widowed were significantly associated with weaker handgrip strength, while having a secondary education level was significantly associated with better handgrip strength. Table 2 gives details of this outcome.

Insert Table 2– ***Summary of prevalence estimates and correlates for frailty and handgrip strength*** – here

3.5. Health-related quality of life

This outcome was reported in 8 studies. Three-quarters of the studies used the World Health Organization Quality of Life (WHOQOL) measure to assess health-related quality of life. The overall mean quality of life scores (out of 100) ranged from 42 to 83 among PLWH50+ on ART. In two studies, PLWH50+ (partially on ART) reported better quality of life scores than their uninfected counterparts [63, 65]. In contrast, four other studies reported a lower quality of life scores [39, 41, 52, 66] among PLWH50+ compared to HIV uninfected older adults or HIV infected young adults.

The correlates of quality of life were reported in only 1 study [65], where having some source of income and being in the upper two wealth quintiles were significantly associated with better quality of life among PLWH50+. Additionally, being female and previously married were associated with reduced odds of good quality of life in PLWH50+. Table 3 gives more details on this outcome.

For peer review only - http://bmjopen.bmj.com/site/about/guidelines.xhtml

Overall, a significant number of the included studies (about 35%) had a low quality score (≤2 – indicating a high risk of bias) [19-22, 26, 29, 31, 35-41, 48, 51, 66]. Specifically, about two-thirds of the included studies did not report participant response rates. Sixteen studies used non-probability sampling methods or did not report their sampling methods. Close to half of the included studies had substantially small samples of PLWH50+ (≤100). Furthermore, a substantial number of studies (about 40%) provided scarce information regarding their measures' reliability and validity. Details of the quality assessment of all included studies are captured in the Supplementary Table 2.

Insert Table 3– ***Summary of results for health-related quality of life*** – here

For peer review only

4.0 DISCUSSION

4.1 Summary of main findings

Our review shows that the research on depression, anxiety, cognitive function, frailty and HRQoL among PLWH50+ in SSA is still scanty and largely concentrated in Uganda, South Africa and Ethiopia. Overall, PLWH50+ presented with wide-ranging prevalence of depression (6% to 59%), cognitive impairment (4% to 61%) and frailty (3% to 15%). Additionally, the correlates of these outcomes were rarely investigated. Nonetheless, those reported were mainly sociodemographic variables, many of which were inconsistent. We find the current evidence inadequate to characterise the real burden and determinants of these outcomes in the region, partly because of the limited number of well-designed studies, heterogeneous findings, and substantial methodological limitations observed in many included studies. Therefore, any policies and programmes about the wellbeing of PLWH50+ in the region have been made on the basis of minimal evidence. The rising numbers of PLWH50+ in SSA and the potentially elevated burden of age-associated comorbidities in this population warrant more research across the region. Such data will provide the evidence needed to develop appropriate policies, programmes, and interventions for the successful aging of PLWH in SSA.

4.2 Depression and anxiety

Depression was the most frequently reported outcome in about half of the included studies. This finding is consistent with the available literature on HIV, which recognises depression as the most commonly occurring psychiatric complication in this population [67]. However, only two studies investigated anxiety, a common mental disorder with a similar prevalence to those seen in depression [68]. The under investigation of anxiety disorders is of particular concern because accumulating research involving PLWH50+ in HICs has documented elevated anxiety levels, as high as 65% [68]. More research is needed on this outcome in the region.

Our review also noted wide-ranging prevalences of depression and anxiety. This finding corroborates previous review findings on the prevalence of common mental disorders in the region, albeit in younger HIV populations [69]. The observed variation could reflect significant contextual differences across the SSA region, e.g., social-environmental milieu,

For peer review only - http://bmjopen.bmj.com/site/about/guidelines.xhtml

healthcare systems, patterns of the burden of diseases, and mental health resources, which are likely to alter the risk profile of these adults [70]. It could also be attributed to potential differences in study populations, study participants, and the use of diverse measurement tools (including different cut-offs for the same measures). Indeed, in the current review, depression was assessed using at least 10 different scoring systems with diverse cut-off points. Besides, the majority of the studies did not provide information on the reliability and validity of the tools they used. Hence, it is difficult to draw firm conclusions regarding the reliability, validity, and applicability of many of the measures used to report this outcome. The PHQ-9, for instance, was frequently used to report depressive symptoms among the included studies. However, most of the studies relied on previous validation of the tool, the context of which was not given. A recent review examining the validation of brief CMDs screening tools in low and middle-income countries did not support the use of PHQ-9 in these settings. It performed poorly in several clinic populations with lower average education but performed very well in high literate populations [71]. This is especially an important consideration in SSA, where older adults are more likely to have low literacy levels. There is a need for rigorous cultural adaptation of these instruments to generate accurate epidemiological data that will inform proper intervention work among PLWH50+ in SSA.

4.3 HIV-associated cognitive impairment

The neurological involvement in HIV disease progression remains problematic, even in well-controlled PLWH, resulting in poor quality of life. HIV associated cognitive impairment is deemed one of the most prevalent comorbidities in the era of ART. Aging with HIV would appear to impose a synergistic impact, whereby increased comorbidities, such as hypertension, diabetes, or depression, likely contribute to and maintain HIV-associated cognitive impairment. In HICs, PLWH50+ have consistently been shown to have high levels of cognitive impairments, as high as 50%. The present review found a wide-ranging prevalence of HIV-associated cognitive impairment (4% to 61%). Our observation is consistent with the available literature, where the prevalence has been noted to vary widely depending on sources used and appears dependent on the populations studied, and approaches used [72].

Brief cognitive screening tools such as the Mini Mental State Examination and International HIV Dementia Scale (IHDS) were frequently used to document the prevalence of cognitive impairment compared to comprehensive neuropsychological batteries. While the use of the brief screeners in low resource settings is well understood given the resource gap, researchers should exercise caution on the tools they use. Recent reviews evaluating brief screening tools for neurocognitive impairment in HIV/AIDS have indicated that some of the commonly utilized tools, e.g. the IHDS perform poorly, especially in screening milder cognitive impairments [73, 74]. In the current review, information on tool reliability or validity was rarely reported. One of the studies reported a limited diagnostic accuracy of the IHDS. However, the Oxford Cognitive Screen-Plus (OCSPlus), one of the most recent screening tools, is promising. So far, the tool has only been used in South Africa among older adults living with HIV, and its initial evaluation yielded excellent construct and external validity [75]. This tool has the potential to address some of the unique challenges and gaps facing resource-limited settings in screening for HAND, including difficulty in performing long test batteries, limited screening tools and a shortage of clinical staff.

For peer review only - http://bmjopen.bmj.com/site/about/guidelines.xhtml

4.4 Frailty

Only two studies quantified the prevalence of frailty among PLWH50+, ranging from 3% to 15%. One of the studies was exclusively conducted among PLWH50+, yielding a low frequency of frailty (3%). In the other study, conducted in rural South Africa, the prevalence of frailty was similar between PLWH50+ and their uninfected counterparts (15% vs 18%, respectively). This finding supports the growing evidence in SSA, which suggest that older on effective ART have similar or better health profiles than their uninfected counterparts, contrary to what has been observed in HICs. At the global stage, the frailty prevalence in SSA appears relatively lower than that of HICs which ranges from 5% to 29% [76]. The population reaching older age in many SSA settings may be relatively fit/active, still working, which may be true of PLWH, potentially favouring better physical health in these individuals. Nonetheless, given the limited number of studies on this outcome in SSA, these findings remain preliminary and inconclusive and highlight the urgent need for more studies to shed more light on these observations.

4.5 Health-related quality of life

For some time, HRQoL has emerged as a salient indicator of HIV care and an essential target for HIV-related research, aiming to expand the service paradigm continuum [77]. This new quality of life frontier has been proposed as the 'fourth 90' to the original '90-90-90' testing and treatment target championed by UNAIDS: to ensure that 90% of people with viral load suppression have good HRQoL [77]. In the current review, research on HRQoL among PLWH50+ was scanty. Thus, little is known on the risk and protective factors of patient-reported health-related quality of life. Further studies should explore the independent effects of aging-related conditions such as common mental disorders, cognitive impairments and frailty on HRQoL among PLWH50+ in SSA to support healthy aging even in the context of a chronic illness such as HIV infection.

Despite the observed limitations, the evidence of better mental health [28, 44, 50, 51, 54, 58, 59], as well as cognition [53, 61], frailty/handgrip strength [47, 62, 63] and quality of life [65], among PLWH50+ on ART (mainly among the robust studies with low risk of bias) compared to uninfected older adults in the current review was striking. This observation could be part of an emerging body of evidence from SSA showing that the mental and wellbeing outcomes among PLWH are comparable to those without HIV. The mechanisms responsible for this observation are not well established and require further examination across many settings in SSA. However, it could be attributed to a few plausible reasons, including i) the direct impact of ART on viral replication, inflammatory pathways, and downstream disease pathophysiology, the so-called "ART advantage" [78, 79], ii) a survivorship bias among PLWH50+ who access and remain in HIV care; and iii) beneficial spillover effects of ART programs such as enhanced access to and utilisation of primary care services resulting in early identification and management of comorbid conditions [79-81]. This observation could also be related to the fact that PLWH50+ on ART are more likely to utilise biomedical facilities due to better education and higher socioeconomic status than their HIV uninfected peers. More studies, preferably longitudinal ones, will shed more light on this observation. As it stands, however, this finding offers an interesting contrast to the commonly reported findings from high-income countries, which generally show that PLWH50+ present with worse physical and mental health outcomes compared to age-matched HIV uninfected

For peer review only - http://bmjopen.bmj.com/site/about/guidelines.xhtml

people [5, 68, 82]. These findings may suggest context-specific differences in the drivers of physical and mental outcomes, including the demographic and risk factor profile as well as the role of engagement in HIV care – among those with and without HIV, especially when comparing low and middle-income to high-income countries. Future studies should explore the extent to which HIV programs could be promoting the wellbeing of PLWH50+ in SSA compared to HIV uninfected older adults in the general population who are likely to bear the brunt of the prevailing poor health systems and primary care systems in the region.

4.6 The correlates of mental and wellbeing outcomes

The correlates of CMDs, cognitive impairment, frailty and HRQoL have been scarcely investigated in SSA. In the current review, the frequently reported correlates were sociodemographic in nature, many of which (e.g. age, formal education levels and wealth status) were inconsistent. However, being female was consistently associated with declining mental health, cognitive function, HRQoL and frailty status. This finding mirrors the results of a recent review of older women's physical and psychological health residing in SSA, which indicated that women were significantly more likely to report poor health compared with men [83]. This finding highlights the need for adequate representation of both sexes in research studies, incorporating a gender lens in their analyses to understand better the unique health challenges that each gender faces. Further studies are also urgently needed in SSA to identify potential risk and protective factors, especially those that could be modified, to suggest how to best integrate the management of these impairments in HIV care guidelines in SSA. Future studies should explore the influence of HIV-related factors, psychosocial risk (e.g. loneliness, ageism, stigma, social support, living arrangements) and other comorbidities on the health and wellbeing of PLWH50+. To promote healthy aging in the context of chronic conditions, such as HIV infection, the psychological and social domains are especially critical as they could be a way to compensate for physiological limitations (that is, multiple chronic conditions) and allow even in the context of disease and disability, to experience optimal quality of life.

4.7 Strengths and Limitations

To our knowledge, this is the first comprehensive review of CMDs, cognitive impairment, frailty and HRQoL among PLWH50+ living in SSA. Our expanded search of 5 online databases and snowballing techniques minimised the risk of missing relevant studies. However, our review should be considered in the context of important limitations. Despite a thorough search strategy, some articles may have been missed, especially in excluding non-English language studies. However, these were very few. The substantial amount of heterogeneity across the studies in terms of, e.g. study design, operationalisation of age, definitions of outcomes, and measures made comparisons difficult and did not allow a meta-analysis to be done. Furthermore, most of the studies recruited PLWH50+ from HIV clinics whose attendees may represent the less-healthy end of the spectrum of service users leading to overestimation of reported outcomes. Conversely, those at the filter end of the cohort may be more able to attend or be proactive concerning their wellbeing, leading to underestimation of outcomes. Besides, the substantial methodological limitations observed might have biased the reported findings, hence limiting their external validity.

For peer review only - http://bmjopen.bmj.com/site/about/guidelines.xhtml

4.8 Conclusions

Overall, the existing research on the burden and determinants of mental and wellbeing outcomes among PLWH50+ in SSA is scantly addressed. We also noted substantial limitations in many of the included studies, which need to be addressed in future studies. Our findings underscore the need to increase the evidence base of these outcomes in the region. Future studies should: a) explicitly target older persons ≥50 years living with HIV, b) carefully select a comparison group and have sufficient sample sizes for adequate statistical testing, c) expand the geographical distribution of these studies in many SSA settings and use adequately standardised survey tools adapted for the region, d) and generate more data on the risk and determinants of these outcomes using a gender lens. There is also an ongoing need for well-designed longitudinal cohort studies to study the temporal patterns of these outcomes in the region.

Contributors: PNM and AA conceptualised and designed the review. PNM and AM reviewed titles, abstracts and full-text papers for eligibility as well as data extraction. PNM wrote the first draft of the manuscript. AM, CN, RW and AA reviewed and edited the prepared manuscript.

Funding

This work was funded by the Wellcome Trust International Master's Fellowship to PNM (Grant number 208283/Z/17/Z). Further funding supporting this work was from 1) the Medical Research Council (Grant number MR/M025454/1) to AA. This award is jointly funded by the UK Medical Research Council (MRC) and the UK Department for International Development (DFID) under MRC/DFID concordant agreement and is also part of the EDCTP2 program supported by the European Union; 2) DELTAS Africa Initiative [DEL-15-003]. The DELTAS Africa Initiative is an independent funding scheme of the African Academy of Sciences (AAS)'s Alliance for Accelerating Excellence in Science in Africa (AESA) and supported by the New Partnership for Africa's Development Planning and Coordinating Agency (NEPAD Agency) with funding from the Wellcome Trust [107769/Z/10/Z] and the UK government. The funders did not have a role in the design and conduct of the study or interpretation of study findings. The views expressed in this publication are those of the author(s) and not necessarily those of AAS, NEPAD Agency, Wellcome Trust or the UK government. For the purpose of Open Access, the author has applied a CC-BY public copyright license to any accepted manuscript version arising from this submission.

Competing interests: The authors have no potential conflict of interest to declare.

Patient consent: Not required

Data sharing statement: No original data were generated for this study. All data underlying the results are available as part of the article, and no additional source data are required.

Acknowledgement

The authors would like to thank the Director of Kenya Medical Research Institute for granting permission to publish this work

For peer review only

Ethics Statement

This work is part of a PhD project to the first author. The project has obtained ethical clearance from the Kenya Medical Research Institute – Scientific and Ethics Review Unit (KEMRI/SERU/CGMR-C/152/3804).

References

1. United Nations, *World population ageing 2019: Highlights*, in *ST/ESA/SER. A/430*. 2019.
2. Autenrieth, C.S., et al., *Global and regional trends of people living with HIV aged 50 and over: Estimates and projections for 2000–2020.* PloS one, 2018. **13**(11): p. e0207005.
3. O'Brien, K.K., et al., *Research priorities for rehabilitation and aging with HIV: a framework from the Canada-International HIV and Rehabilitation Research Collaborative (CIHRRC).* AIDS Research and Therapy, 2020. **17**: p. 1-13.
4. Sabin, C.A. and P. Reiss, *Epidemiology of ageing with HIV: what can we learn from cohorts?* Aids, 2017. **31**: p. S121-S128.
5. Brennan-Ing, M., J. Ramirez-Valles, and A. Tax, *Aging With HIV: Health Policy and Advocacy Priorities.* Health Education & Behavior, 2021. **48**(1): p. 5-8.
6. Milic, J., et al., *European cohorts of older HIV adults: POPPY, AGE h IV, GEPPO, COBRA and FUNCFRAIL.* European Geriatric Medicine, 2019. **10**(2): p. 247-257.
7. Brothers, T.D. and K. Rockwood, *Frailty: a new vulnerability indicator in people aging with HIV.* European Geriatric Medicine, 2019. **10**(2): p. 219-226.
8. Gustafson, D., et al., *Frailty and constellations of factors in aging HIV-infected and uninfected women-the women's interagency HIV study.* The Journal of frailty & aging, 2016. **5**(1): p. 43.
9. Levett, T., *Frailty Prevalence and Predictors in Older Adults with HIV*. 2017, University of Brighton.
10. Saka, S., F. Oosthuizen, and M. Nlooto, *national policies and older people's healthcare in Sub-Saharan Africa: a scoping review.* Annals of global health, 2019. **85**(1).
11. Nabalamba, A. and M. Chikoko, *Aging population challenges in Africa*. 2011: African Development Bank, Chief Economist Complex.
12. Joint United Nations Programme on HIV/AIDS, *The Gap Report. Geneva, Switzerland: UNAIDS*. 2014.
13. World Health Organization. *Health statistics and information systems: SAGE HIV studies*. 2020 [cited 2020 June 23]; Available from: https://www.who.int/healthinfo/sage/hiv_studies/en/.
14. Gómez-Olivé, F.X., et al., *Cohort profile: health and ageing in Africa: a longitudinal study of an INDEPTH community in South Africa (HAALSI).* International journal of epidemiology, 2018. **47**(3): p. 689-690j.
15. National Institutes of Health. *Ugandan Non-Communicable Diseases and Aging Cohort (UGANDAC)*. 2020 [cited 2020 June 23]; Available from: https://clinicaltrials.gov/ct2/show/NCT02445079.
16. Page, M.J., et al., *PRISMA 2020 explanation and elaboration: updated guidance and exemplars for reporting systematic reviews.* bmj, 2021. **372**.
17. Stang, A., *Critical evaluation of the Newcastle-Ottawa scale for the assessment of the quality of nonrandomized studies in meta-analyses.* European journal of epidemiology, 2010. **25**(9): p. 603-605.
18. Filteau, S., et al., *Nutritional status is the major factor affecting grip strength of African HIV patients before and during antiretroviral treatment.* Trop Med Int Health, 2017. **22**(10): p. 1302-1313.

For peer review only

For peer review only - http://bmjopen.bmj.com/site/about/guidelines.xhtml

19. Akena, D., et al., *The association between aids related stigma and major depressive disorder among HIV-positive individuals in Uganda.* PLoS One, 2012. **7**(11): p. e48671.
20. Asangbeh, S.L., et al., *Predictors of depression among patients on art in a rural health district in North West Cameroon.* AIDS Care, 2016. **28**(2): p. 205-8.
21. Atashili, J., et al., *Prevalence, characteristics and correlates of a positive-dementia screen in patients on antiretroviral therapy in Bamenda, Cameroon: a cross-sectional study.* BMC Neurol, 2013. **13**: p. 86.
22. Cassimjee, N. and P.K. Motswai, *Neuropsychological profiles of adults and older adults with HIV.* South African Journal of Psychology, 2017. **47**(1): p. 35-45.
23. Cholera, R., et al., *Depression and Engagement in Care Among Newly Diagnosed HIV-Infected Adults in Johannesburg, South Africa.* AIDS & Behavior, 2017. **21**(6): p. 1632-1640.
24. Duko, B., et al., *Prevalence and associated factors of depression among patients with HIV/AIDS in Hawassa, Ethiopia, cross-sectional study.* Annals of General Psychiatry, 2018. **17**.
25. Eaton, P., et al., *Risk factors for symptomatic HIV-associated neurocognitive disorder in adults aged 50 and over attending a HIV clinic in Tanzania.* Int J Geriatr Psychiatry, 2020.
26. Harding, R., et al., *Quality of life and wellbeing among HIV outpatients in East Africa: a multicentre observational study.* BMC Infect Dis, 2014. **14**: p. 613.
27. Kaharuza, F.M., et al., *Depression and CD4 cell count among persons with HIV infection in Uganda.* AIDS Behav, 2006. **10**(4 Suppl): p. S105-11.
28. Manne-Goehler, J., et al., *Depressive symptoms before and after antiretroviral therapy initiation among older-aged individuals in rural Uganda.* AIDS and Behavior, 2019. **23**(3): p. 564-571.
29. Musinguzi, K., et al., *Association between major depressive disorder and pro-inflammatory cytokines and acute phase proteins among HIV-1 positive patients in Uganda.* BMC Immunol, 2018. **19**(1): p. 1.
30. Olagunju, A.T., et al., *Factors associated with anxiety disorders among HIV-positive attendees of an HIV clinic in Lagos, Nigeria.* Int J STD AIDS, 2012. **23**(6): p. 389-93.
31. Olley, B.O., et al., *Psychosocial factors predicting severity of depression among treatment-seeking HIV/AIDS patients: A multi-site Nigerian study.* Niger J Clin Pract, 2017. **20**(3): p. 296-302.
32. Shumba, C., et al., *Prevalence of Depressive Symptoms Amongst Highly Active Antiretroviral Therapy (HAART) Patients in AIDSRelief Uganda.* J Public Health Afr, 2013. **4**(2): p. e19.
33. Ssonko, M., et al., *Polypharmacy among HIV positive older adults on anti-retroviral therapy attending an urban clinic in Uganda.* BMC Geriatr, 2018. **18**(1): p. 125.
34. Tesfaw, G., et al., *Prevalence and correlates of depression and anxiety among patients with HIV on-follow up at Alert Hospital, Addis Ababa, Ethiopia.* BMC Psychiatry, 2016. **16**(1): p. 368.
35. Tsegaw, M., et al., *Screening HIV-associated neurocognitive disorders (HAND) among HIV positive patients attending antiretroviral therapy in South Wollo, Ethiopia.* J Psychiatr Res, 2017. **85**: p. 37-41.
36. Berhe, H. and A. Bayray, *Prevalence of depression and associated factors among people living with HIV/AIDS in tigray, North Ethiopia: A cross sectional hospital based study.* International Journal of Pharmaceutical Sciences and Research, 2013. **4**(2): p. 765.
37. Eshetu, D.A., et al., *Prevalence of depression and associated factors among HIV/AIDS Patients attending ART Clinic at Debrebirhan referral hospital, North Showa, Amhara Region, Ethiopia.* Clin Psychiatry, 2015. **1**(1): p. 1-7.
38. Mugendi, A., et al., *Prevalence and Correlates of Neurocognitive Disorders among HIV patients on antiretroviral therapy at a Kenyan Hospital.* Neurology research international, 2019. **2019**.

For peer review only

For peer review only - http://bmjopen.bmj.com/site/about/guidelines.xhtml

39. Obimakinde, A.M., et al., *Going Beyond Giving Antiretroviral Therapy: Multimorbidity in Older People Aging with HIV in Nigeria.* AIDS research and human retroviruses, 2020. **36**(3): p. 180-185.
40. Oumar, G., et al., *Neurocognitive Disorders in People Living with Human Immunodeficiency Virus Aged 50 Years Old and More in Yalgado Ouédraogo Teaching Hospital.* American Journal of Internal Medicine, 2020. **8**(4): p. 177-181.
41. Parcesepe, A.M., et al., *Gender, HIV-related stigma, and health-related quality of life among adults enrolling in HIV Care in Tanzania.* AIDS and Behavior, 2020. **24**(1): p. 142-150.
42. Torgersen, J., et al., *Impact of efavirenz metabolism on loss to care in older HIV+ Africans.* European Journal of Drug Metabolism and Pharmacokinetics, 2019. **44**(2): p. 179-187.
43. Abadiga, M., *Depression and its associated factors among HIV/AIDS patients attending ART clinics at Gimbi General hospital, West Ethiopia, 2018.* BMC Research Notes, 2019. **12**(1): p. 527.
44. Asrat, B., et al., *Major depressive disorder and its association with adherence to antiretroviral therapy and quality of life: cross-sectional survey of people living with HIV/AIDS in Northwest Ethiopia.* BMC psychiatry, 2020. **20**(1): p. 1-13.
45. Bernard, C., et al., *Prevalence and factors associated with physical function limitation in older West African people living with HIV.* PloS one, 2020. **15**(10): p. e0240906.
46. Bernard, C., et al., *Prevalence and factors associated with severe depressive symptoms in older west African people living with HIV.* BMC psychiatry, 2020. **20**(1): p. 1-11.
47. Bristow, C., et al., *Low levels of frailty in HIV-positive older adults on antiretroviral therapy in northern Tanzania.* Journal of NeuroVirology, 2021: p. 1-12.
48. Kalomo, E.N., et al., *HIV stigma, resilience and depressive symptoms among older adults living with HIV in rural Namibia.* African Journal of AIDS Research, 2020. **19**(3): p. 198-205.
49. Kellett-Wright, J., et al., *Screening for HIV-Associated Neurocognitive Disorder (HAND) in Adults Aged 50 and Over Attending a Government HIV Clinic in Kilimanjaro, Tanzania. Comparison of the International HIV Dementia Scale (IHDS) and IDEA Six Item Dementia Screen.* AIDS and Behavior, 2020: p. 1-12.
50. Motumma, A., et al., *Prevalence and associated factors of common mental disorders among adult patients attending HIV follow up service in Harar town, Eastern Ethiopia: a cross-sectional study.* BMC psychology, 2019. **7**(1): p. 1-9.
51. Moucheraud, C., et al., *A Multi-Dimensional Characterization of Aging and Wellbeing Among HIV-Positive Adults in Malawi.* AIDS and Behavior, 2020: p. 1-11.
52. Yaya, I., et al., *Health-related quality of life among people living with HIV/AIDS in Togo: individuals and contextual effects.* BMC Research Notes, 2019. **12**(1): p. 140.
53. Kobayashi, L.C., et al., *Cognitive function and impairment in older, rural south African adults: evidence from "health and aging in Africa: a longitudinal study of an INDEPTH Community in Rural South Africa".* Neuroepidemiology, 2019. **52**(1-2): p. 32-40.
54. Geldsetzer, P., et al., *Depressive symptoms and their relation to age and chronic diseases among middle-aged and older adults in rural South Africa.* The Journals of Gerontology: Series A: Biological Sciences and Medical Sciences, 2019. **74**(6): p. 957-963.
55. Kinyanda, E., et al., *Risk of major depressive disorder among older persons living in HIV-endemic central and southwestern Uganda.* AIDS Care, 2016. **28**(12): p. 1516-1521.
56. Mugisha, J.O., et al., *Chronic disease, risk factors and disability in adults aged 50 and above living with and without HIV: findings from the Wellbeing of Older People Study in Uganda.* Glob Health Action, 2016. **9**: p. 31098.
57. Negin, J., et al., *Prevalence of HIV and chronic comorbidities among older adults.* Aids, 2012. **26 Suppl 1**(0 1): p. S55-63.
58. Nyirenda, M., et al., *Prevalence and correlates of depression among HIV-infected and-affected older people in rural South Africa.* Journal of affective disorders, 2013. **151**(1): p. 31-38.

For peer review only - http://bmjopen.bmj.com/site/about/guidelines.xhtml

59. Rohr, J.K., et al., *HIV treatment cascade for older adults in rural South Africa.* Sexually Transmitted Infections, 2020. **96**(4): p. 271-276.
60. Joska, J.A., et al., *Prevalence of HIV-1 Infection in an elderly rural population and associations with neurocognitive impairment.* Aids, 2019. **33**(11): p. 1765-1771.
61. Asiimwe, S.B., et al., *Cognitive differences associated with HIV serostatus and antiretroviral therapy use in a population-based sample of older adults in South Africa.* Scientific reports, 2020. **10**(1): p. 1-14.
62. Edwards, A., et al., *HIV serostatus, inflammatory biomarkers and the frailty phenotype among older people in rural KwaZulu-Natal, South Africa.* African Journal of AIDS Research, 2020. **19**(3): p. 177-185.
63. Mugisha, J., et al., *Caregiving responsibilities and burden among older people by HIV status and other determinants in Uganda.* AIDS Care, 2013. **25**(11): p. 1341-8.
64. Scholten, F., et al., *Health and functional status among older people with HIV/AIDS in Uganda.* BMC Public Health, 2011. **11**: p. 886.
65. Nyirenda, M., et al., *An investigation of factors associated with the health and well-being of HIV-infected or HIV-affected older people in rural South Africa.* BMC Public Health, 2012. **12**(1): p. 259-259.
66. Maniragaba, F., et al., *Predictors of quality of life of older persons in rural Uganda: A cross sectional study.* AAS Open Res, 2018. **1**: p. 22.
67. Nanni, M.G., et al., *Depression in HIV infected patients: a review.* Current psychiatry reports, 2015. **17**(1): p. 530.
68. Brennan-Ing, M., *Emerging issues in HIV and aging*. 2020.
69. Too, E.K., et al., *Prevalence and factors associated with common mental disorders in young people living with HIV in sub-Saharan Africa: a systematic review.* Journal of the International AIDS Society, 2021. **24**: p. e25705.
70. Nyirenda, M., et al., *Health, wellbeing, and disability among older people infected or affected by HIV in Uganda and South Africa.* Global health action, 2013. **6**(1): p. 19201.
71. Ali, G.-C., G. Ryan, and M.J. De Silva, *Validated screening tools for common mental disorders in low and middle income countries: a systematic review.* PloS one, 2016. **11**(6): p. e0156939.
72. Alford, K. and J. Vera, *Cognitive impairment in people living with HIV in the ART era: a review.* British medical bulletin, 2018. **127**(1): p. 55-68.
73. Zipursky, A.R., et al., *Evaluation of brief screening tools for neurocognitive impairment in HIV/AIDS: a systematic review of the literature.* AIDS (London, England), 2013. **27**(15): p. 2385.
74. Mwangala, P.N., et al., *Screening tools for HIV-associated neurocognitive disorders among adults living with HIV in sub-Saharan Africa: A scoping review.* AAS open research, 2018. **1**.
75. Humphreys, G.W., et al., *Cognitive function in low-income and low-literacy settings: Validation of the tablet-based Oxford Cognitive Screen in the Health and Aging in Africa: A Longitudinal Study of an INDEPTH community in South Africa (HAALSI).* The Journals of Gerontology: Series B, 2017. **72**(1): p. 38-50.
76. Levett, T.J., et al., *Systematic review of prevalence and predictors of frailty in individuals with human immunodeficiency virus.* Journal of the American Geriatrics Society, 2016. **64**(5): p. 1006-1014.
77. Lazarus, J.V., et al., *Beyond viral suppression of HIV–the new quality of life frontier.* BMC medicine, 2016. **14**(1): p. 94.
78. Pala, A.N., et al., *Subtypes of depressive symptoms and inflammatory biomarkers: An exploratory study on a sample of HIV-positive patients.* Brain, behavior, and immunity, 2016. **56**: p. 105-113.
79. Manne-Goehler, J., et al., *The ART advantage: healthcare utilization for diabetes and hypertension in rural South Africa.* Journal of acquired immune deficiency syndromes (1999), 2017. **75**(5): p. 561.

For peer review only - http://bmjopen.bmj.com/site/about/guidelines.xhtml

80. Feinstein, M.J., et al., *Ideal cardiovascular health and carotid atherosclerosis in a mixed cohort of HIV-infected and uninfected Ugandans.* AIDS research and human retroviruses, 2017. **33**(1): p. 49-56.
81. Wouters, E., et al., *Physical and emotional health outcomes after 12 months of public-sector antiretroviral treatment in the Free State Province of South Africa: a longitudinal study using structural equation modelling.* BMC Public Health, 2009. **9**(1): p. 103.
82. Langebeek, N., et al., *Impact of comorbidity and ageing on health-related quality of life in HIV-positive and HIV-negative individuals.* Aids, 2017. **31**(10): p. 1471-1481.
83. Mangipudi, S., T. Cosco, and S. Harper, *A systematic review of physical and psychological health and wellbeing of older women in Sub-Saharan Africa.* Journal of Public Health, 2020. **42**(2): p. 294-303.

For peer review only

For peer review only - http://bmjopen.bmj.com/site/about/guidelines.xhtml

Table 1: Summary of prevalence estimates and correlates for cognitive impairment/neurological impairment

Author, publication year & country	Study design	Sample size	Treatment status of PLWH	Assessment tool used	Cut-off score or diagnosis criteria	Information on local tool validation	Prevalence estimates/other results for PLWH50+	Correlates reported
Studies reporting the prevalence of HIV-associated neurocognitive disorders (HAND) or cognitive impairment								
Kellett-Wright, J., et al. (2020) ; Tanzania [49]	Cross-sectional	235 PLWH50+	95.5% on ART	International HIV Dementia Scale (IHDS) and IDEA (Six Item Dementia Screen)	Frascati criteria with a detailed consensus panel discussion	Diagnostic accuracy (area under the receiver operating characteristic curve: 0.64–0.67 for IHDS and 0.65–0.71 for IDEA	Prevalence of symptomatic HAND (MND and HAD) was 25.3%.	Not reported
Eaton, P., et al. (2020); Tanzania [25]	Cross-sectional	253 PLWH50+	94.8% on ART	Comprehensive NP battery	Frascati criteria for classifying HAND	NR	Prevalence of symptomatic HAND (HAD and MND) was 21.7%.	PLWH were at higher risk of having symptomatic HAND if they lived alone [OR = 2.6], were illiterate (OR= 3.2) or older at the time of HIV diagnosis (OR = 1.1). HIV-specific factors were not related to symptomatic HAND. Vascular risk factors, such as smoking status, were not associated with symptomatic HAND.
Oumar, G., et al. (2020); Burkina Faso [40]	Cross-sectional	102 PLWH50+	All on ART	Mini Mental State Examination	Frascati criteria for classifying HAND	NR	23.5% had neurocognitive disorders	In a multivariate analysis, age (OR=4.6) and educational status (OR=2.6) were associated with neurocognitive disorders.
Mugendi, A., et al. (2019); Kenya [38]	Cross-sectional	64 PLWH50+; 281 PLWH (20-49 years)	All on ART	Montreal Cognitive Assessment (MoCA) and IHDS	Combined scores of the 2 tools: a score of ≤26 on MOCA and ≤10 on the IHDS.	NR	Among PLWH50+, symptomatic HAND was 19.2%	Reported correlates not aggregated by age.
Tsegaw, M., et al. (2017); Ethiopia [35]	Cross-sectional	67PLWH56+; 526 PLWH (18-55 years)	All on ART	International HIV Dementia Scale	≤9.5	NR	61.2% among PLWH56+ had HAND	Reported correlates not aggregated by age.
Kobayashi, L. C., et al. (2019); South Africa [53]	Population survey	1,048 PLWH40+; 4,011 HIV uninfected older adults ≥40 years	63.9% on ART	Brief cognitive battery (orientation, immediate & delayed recall).	≤ 1.5 SDs below the mean composite time orientation and memory score.	Locally adapted	4.0% of PLWH40+ had cognitive impairment compared to 9.0% of uninfected older adults ≥40 years	Independent predictors of lower cognitive score were being older, female, unmarried, not working, having low formal education, low socioeconomic status, and having a history of cardiovascular conditions.
Ssonko, M., et al. (2018); Uganda [33]	Cross-sectional	411 PLWH50+	All on ART	Folstein's Mini-Mental State Examination	NR	NR	Mild cognitive impairment was associated with falls in the previous 12 months.	NR

For peer review only - http://bmjopen.bmj.com/site/about/guidelines.xhtml

Author, publication year & country	Study design	Sample size	Treatment status of PLWH	Assessment tool used	Cut-off score or diagnosis criteria	Information on local tool validation	Prevalence estimates/other results for PLWH50+	Correlates reported
Studies reporting neuropsychological test scores								
Asiimwe, S. B., et al. (2020); South Africa [61]	Population survey	1048 PLWH40+; 3512 HIV uninfected older adults ≥40 years	68.6% on ART	A battery of conventional instruments and the Oxford Cognitive Screen-Plus (OCS-Plus)	Summarised mean cognitive scores between participants with and without HIV on the two measures.	Previously validated in the study setting	PLWH scored on average 0.06 (95% CI 0.01–0.12) standard deviation (SD) units higher on the conventional cognitive function measure and 0.02 (95% CI − 0.07 to 0.04) SD units lower on the OCS-Plus measure than HIV-negative participants.	ART use was non-significantly associated with better cognitive outcomes among PLWH in both instruments.
Moucheraud, C., et al. (2020); Malawi [51]	Cross-sectional	74 PLWH50+ and 60 young PLWH (30-49) years	All on ART	Brief cognitive battery (MMSE)	NR	NR	Mean cognitive scores: 16.0 among PLWH50+ versus 17.4 among young PLWH.	NR
Cassimjee, N. and P. K. Motswai (2017); South Africa [22]	Cross-sectional	33 PLWH50+; 17 HIV uninfected older adults [mean age 52.2]	NR	Dementia Rating Scale-2, the Stroop Colour and Word Test, the Symbol Digits Test and the D-KEFS Trail Making Test.	N/A	NR	PLWH, in comparison to the uninfected group, had significantly poorer performance profiles in global cognitive functioning, memory, executive functioning, psychomotor functioning, and processing speed.	Not reported
Studies reporting dementia								
Joska, J. A., et al. (2019); South Africa [60]	Population survey	55 PLWH50+; 1,095 HIV uninfected older adults ≥50 years	86.1% on ART	Brief Community Screening Instrument for Dementia	NR	Previously validated	18.2% of PLWH50+ had dementia compared to 10.7 % of the uninfected older adults ≥50 years	Dementia status was associated with HIV infection (OR= 2.2, CI=1–4.5), older age (OR =1.0, CI=1.0–1.1) and f depressive symptoms (OR 2.7, CI=1.8–3.9).
Atashili, J., et al. (2013); Cameroon [21]	Cross-sectional	400 HIV infected adults (20-53 years old)	All on ART	International HIV Dementia Scale (IHDS)	≤10 on IHDS	Not validated	The odds of having a positive dementia screen increased progressively with increasing participants' age,	Not aggregated by age
Studies reporting HIV-associated neuropathy								
Obimakinde, A. M., et al. (2020); Nigeria [39]	Cross-sectional	62 PLWH60+; 162 HIV uninfected older adults ≥60 years	All on ART	Extracted from participants medical notes	Clinical notes	NR	19.0% in PLWH60+ had peripheral neuropathy	NR

Notes: ART – *Antiretroviral treatment*; **HAD** – *HIV-associated* dementia; **HAND** – *HIV-associated neurocognitive disorders*; **IDEA –** Item Dementia Screen**; IHDS** – *International HIV dementia scale*; **MMSE** – *Mini-mental Status Examination*; **MND** –*Mild neurocognitive* disorder; **NA** – *Not* applicable; **NR** – *Not* reported; SD – *Standard* deviation; **OR –** *Odds ratio*; **PLWH –** *People living with HIV*; **PLWH40+ -** *People living with HIV ≥40* years; **PLWH50+ –** *People living with HIV ≥50 years old*

For peer review only - http://bmjopen.bmj.com/site/about/guidelines.xhtml

Table 2: Summary of prevalence estimates and correlates for frailty and handgrip strength

Author, publication year & country	Study design	Sample size	Treatment status of PLWH	Assessment tool used	Cut-off score	Tool validation	Prevalence estimates/other results for PLWH50+	Correlates reported
**Studies that assessed frailty comprehensively**								
Bristow, C., et al. (2021); Tanzania. [47]	Cross-sectional	145 PLWH50+	99.3% on ART	Fried frailty phenotype (FFP), Clinical Frailty Scale (CFS) and B-FIT 2).	**FFP:** A score of 1–2 was pre-frail, and a score of ≥ 3 was frail; **B-FIT** (A score of 8/20); **CFS** (a score of 5–9)	BFIT and FFP previously validated in Tanzania.	2.8% (95% CI 0.09–5.4%) when using **the FFP.** 0.68% (CI − 0.66–2.04%) when using **the CFS or B-FIT 2).**	FFP frailty was associated with female gender (p = 0.006), marital status (p = 0.007) and age (p = 0.038). Weight loss was the most common FFP domain failure.
Edwards, A., et al. (2020); South Africa [62]	Cross-sectional	292 PLWH50+ and 322 HIV uninfected adults (≥50) years.	NR	Fried Frailty Phenotype (FFP)	Presence of ≥3 of the 5 FFP phenotypes	Adapted in the local population	Frailty was similar for HIV negative and PWH (17.7% vs 14.7%, p = 0.72) respectively as was pre-frailty status (66.5% vs 63.4%).	The prevalence of frailty increased with age. There was no association between any of the inflammatory biomarkers and frailty and pre-frailty.
Ssonko, M., et al. (2018); Uganda [33]	Cross-sectional	411 PLWH50+	All on ART	Self-report 40 -item frailty index questionnaire	NR	NR	Frailty index scores of 5 to 6 (PR = 10.6, 95% C.I. 1.4, 78, p = 0.02), and 7 or more (PR = 17.4, 95% C.I. 2.4, 126.5, p = 0.005) were associated with polypharmacy.	Increasing Frailty score was associated with increasing levels of polypharmacy.
Studies that assessed handgrip strength								
Bernard, C., et al. (2020); Coˆte d'Ivoire & Senegal [45]	Cross-sectional	333 PLWH50+	All on ART	Calibrated Jamar hydraulic hand dynamometer	A performance less to the median obtained in each gender group (male/female)	Not reported	43.2% of women had low grip strength. 42.5% of men had low grip strength.	NR
Filteau, S., et al. (2017); Zambia and Tanzania [18]	Randomized trial	PLWH50+: 148 at baseline; 72 at 12wks, and 50 at 2-3 years ART	ART naïve at baseline	Digital handgrip dynamometer	NA	NR	Mean grip strength change: 18.7 kgs at bassline, 20.4 kgs at 12 weeks and 23.1 kgs at 2-3 years.	Reported correlates not aggregated by age.
Mugisha, J., et al. (2013); Uganda [63]	Cross-sectional	198 PLWH50+; 311 HIV uninfected adults ≥50 years	51.0% on ART	Digital handgrip dynamometer	NA	NR	25.7 among PLWH50+; and 22.1 among the uninfected older adults ≥50 years.	NR

For peer review only - http://bmjopen.bmj.com/site/about/guidelines.xhtml

Author, publication year & country	Study design	Sample size	Treatment status of PLWH	Assessment tool used	Cut-off score	Tool validation	Prevalence estimates/other results for PLWH50+	Correlates reported
Negin, J., et al. (2012); South Africa [57]	Cross-sectional	142 PLWH50+; 2,722 HIV uninfected older adults ≥50 years	NR	Digital handgrip dynamometer	NA	Locally validated	Mean Grip strength in PLWH50+ (33.5 kg) was weaker than HIV-uninfected individuals (38.3 kg).	When controlling for age and sex among those aged 50 years and older, HIV status was significantly associated with weaker grip strength.
Scholten, F., et al. (2011); Uganda [64]	Cross-sectional	189 PLWH50+; 2017 HIV affected 50+; and 104 HIV uninfected older adults ≥50 years	51.0% on ART	Digital handgrip dynamometer	NA	Locally adapted	25.8 among PLWH50+; and 25.3 among HIV affected 50+ and 25.2 among the uninfected older adults ≥50 years.	Women scored poorly than men. Married respondents had significantly better scores than widowed and divorced respondents. Respondents with at least secondary education had significantly better scores.

Notes: ART – *Antiretroviral treatment*; **B-FIT 2** – *Brief Frailty Instrument of Tanzania*; **CFS** – *Clinical Frailty Scale*; **FFP** – *Fried Frailty Phenotype*; **NA** – *Not* applicable; **NP** – Neuropsychological; **NR** – *Not* reported; **PLWH** – *People living with HIV*; **PLWH50+** – *People living with HIV ≥50 years old*; **PR** – *Prevalence ratio*

For peer review only - http://bmjopen.bmj.com/site/about/guidelines.xhtml

Table 3: Summary of results for health-related quality of life

Author, publication year & country	Study design	Sample size	Treatment status of PLWH	Assessment tool used	Cut-off score	Information on local tool validation	Prevalence estimates/other results	Correlates reported
**Studies that utilized different versions of the World Health Organization Quality-of-Life Scale (WHOQOL)**								
Obimakinde, A. M., et al. (2020); Nigeria [39]	Cross-sectional	62 PLWH60+; 124 HIV uninfected older adults ≥60 years	All on ART	8-item WHOQoL	NA	NR	Mean QoL (SD): 82.7 (8.9) among PLWH50+; 86.2 (6.5) in the uninfected adults ≥50 years.	NR
Mugisha, J., et al. (2013); Uganda [63]	Cross-sectional	198 PLWH50+; 311 HIV uninfected older adults ≥50 years	51.0% on ART	8-Item WHOQoL	NA	NR	Mean QoL scores: 57.2 among PLWH50+; and 45.3 in the uninfected older adults ≥50 years	NR
Nyirenda, M., et al. (2013); Uganda and South Africa [70]	Cross-sectional	400 PLWH50+; 732 HIV uninfected older adults ≥50 years	About 22% on ART	8-Item WHOQoL	NA	Locally adapted and validated	Older people in South Africa were more likely to have a better quality of life (aOR 2.15, 95% CI 1.60–2.90) than in Uganda.	NR
Nyirenda, M., et al. (2012); South Africa [65]	Cross-sectional	203 PLWH50+; 219 HIV uninfected older adults ≥50 years	23.7% on ART	8-Item WHOQoL	NA	Locally adapted	Mean QoL scores: 62.5 among PLWH50+; and 59.4 in the uninfected older adults ≥50 years.	Being female and having been previously married was associated with lower odds of good QoL in PLWH50+
Yaya, I., et al. (2019) ; Togo [52]	Cross-sectional	154 PLWH50+; 726 PLWH (15-49 years)	90.5% on ART	31- Item WHOQOL-HIV BREF	≥77.3 was considered good quality of life.	NR	72.7% of PLWH50+ had a good global quality of life compared to 77.3% in the younger PLWH.	Correlates not aggregated by age
Maniragaba, F., et al. (2018) ; Uganda [66]	Cross-sectional	28 PLWH50+; 884 HIV uninfected older adults ≥60 years	NR	An adapted version of the WHOQOL-OLD	An index (good, fair and poor quality of life) .	NR	PLWH had poor quality of life (OR=0.45; CI=0.22-0.93) compared to those who were HIV uninfected.	Not aggregated by HIV status
Studies that used other measures of quality of life								
Parcesepe, A. M., et al. (2020); Tanzania [41]	Cross-sectional	82 PLWH50+; 824 PLWH (18-49 years)	ART naïve	HIV/AIDS-Targeted Quality of Life Scale	NA	Previously validated in other SSA settings	Mean QoL score: 68 among PLWH50+, and 72.4 among PLWH (18-49 years)	Reported correlates not aggregated by age.
Harding, R., et al. (2014); Kenya and Uganda [26]	Cross-sectional	1,337 adults aged 18-70 years (*number of PLWH50+ NR)*	49.9% on ART	Medical Outcome Scale -HIV (MOS-HIV)	NA	Previously validated in other SSA setting	**Mean Mental Health score:** 46.4 among PLWH50+; 46.2 in PLWH (18-49 years) **Physical health score**: 42.9 among PLWH50+; and 44.8 in PLWH (18-49 years)	Reported correlates not aggregated by age.

Notes: ART – *Antiretroviral treatment*; **AOR –** *Adjusted odds ratio*; **OR –** *Odds ratio*; **CI –** *Confidence interval*; **NA –** *Not* applicable; **NR –** *Not reported*; **PLWH –** *People living with HIV*; **PLWH50+ –** *People living with HIV ≥50 years old*; **QoL –** *Quality of* life; **SSA** – *sub-Saharan* Africa; **SD –** *Standard deviation*; **WHOQOL** – *World Health Organization Quality of life measure*

For peer review only - http://bmjopen.bmj.com/site/about/guidelines.xhtml

Figure 1: Identification of eligible studies

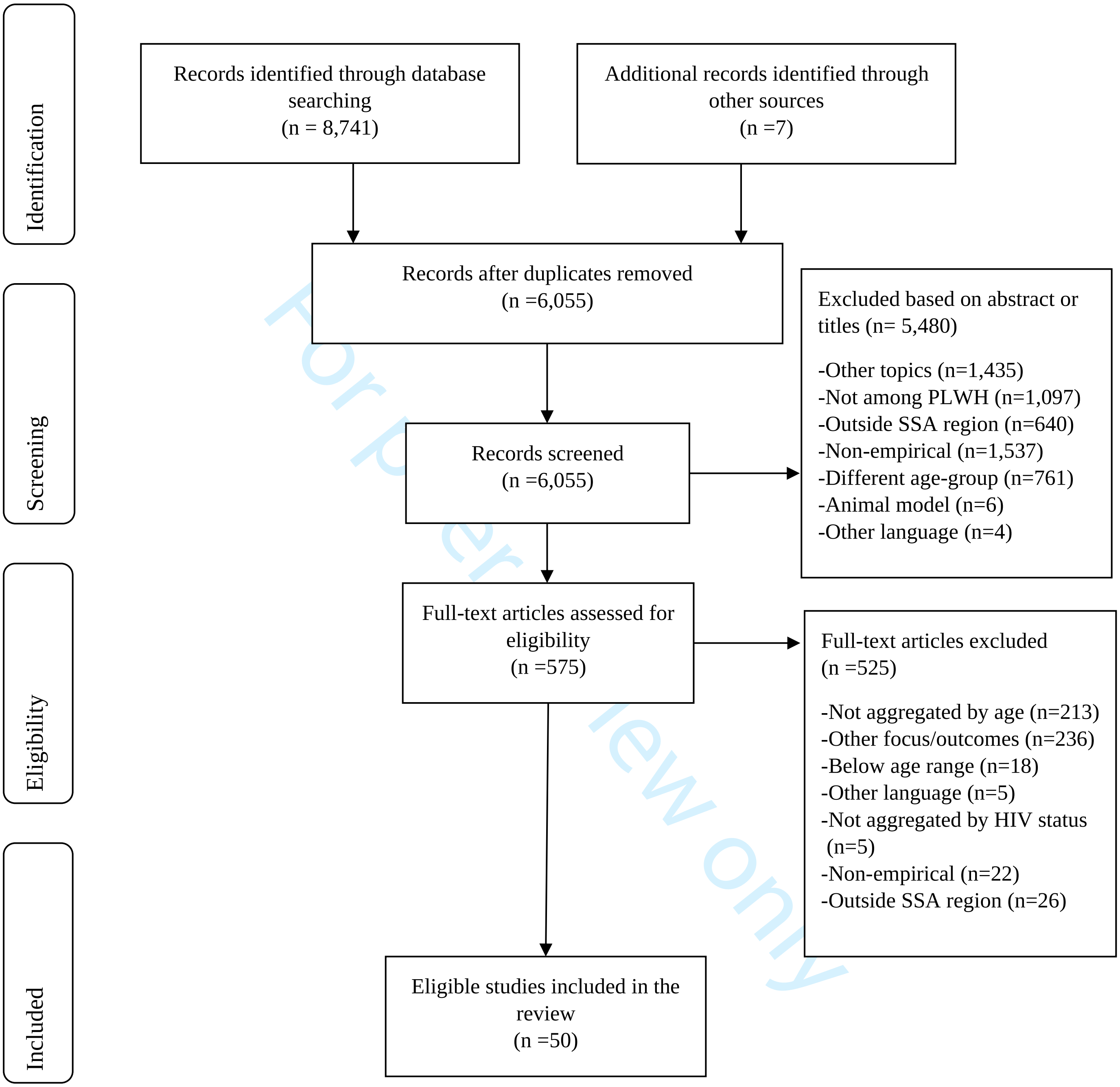

Figure 2: Geographical distribution of the included studies within SSA

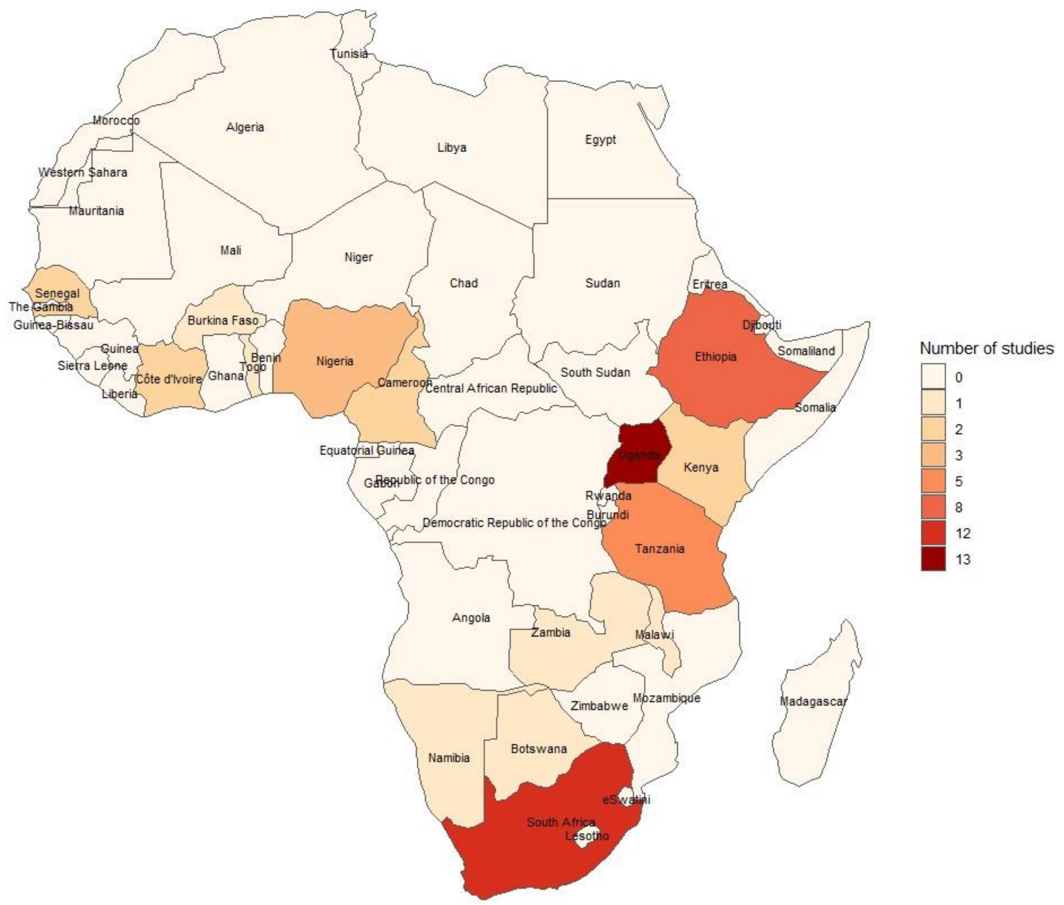

Supplementary Files – Mental health and Wellbeing of older adults living with HIV in sub-Saharan Africa: a systematic review

Supplementary file 1. Search strategy

We adopted a stepwise approach that involves first combining shorter keywords for each part of the PICOS criteria with “OR” and later checking history for the list of searches and combining them with “AND.” No filters were applied.

The following search terms were included:

- HIV OR HIV-1 OR HIV/AIDS OR HIV infections
- adult OR older adult OR older people OR older individual OR elderly
- Africa OR sub-Saharan Africa OR Africa South of the Sahara
- cognitive impairment OR neurocognitive impairment OR neurological complication OR HIV-associated neurocognitive disorder
- common mental disorder OR depression OR depressive symptoms OR depressive disorder OR anxiety OR anxiety disorder
- quality of life OR health-related quality of life
- grip strength OR hand strength OR frailty
-

<u>PubMed search</u> – 13/01/2021:

((((((HIV) OR (HIV-1)) OR (HIV/AIDS)) OR (HIV infections)) AND (((((Adult) OR (older adult)) OR (older people)) OR (older individual)) OR (elderly))) AND (((Africa) OR (sub-Saharan Africa)) OR (Africa South of the Sahara))) AND ((((((((((((((cognitive impairment) OR (neurocognitive impairment)) OR (neurological complication)) OR (HIV-associated neurocognitive disorder)) OR (common mental disorder)) OR (depression)) OR (depressive symptoms)) OR (depressive disorder)) OR (anxiety)) OR (anxiety disorder)) OR (quality of life)) OR (health-related quality of life)) OR (grip strength)) OR (hand strength)) OR (frailty))

Number of hits = 2,090

<u>PsycINFO search – 13/01/2021</u>:

((HIV or HIV-1 or HIV AIDS or HIV infections) and (adult or older adult or older people or older individual or elderly) and (Africa or sub-Saharan Africa or Africa South of the Sahara) and (cognitive impairment or neurocognitive impairment or neurological complication or HIV-associated neurocognitive disorder or common mental disorder or depression or depressive symptoms or depressive disorder or anxiety or anxiety disorder or quality of life or health-related quality of life or grip strength or hand strength or frailty)).af.

Number of hits = 3,691

For peer review only - http://bmjopen.bmj.com/site/about/guidelines.xhtml

Supplementary Files – Mental health and Wellbeing of older adults living with HIV in sub-Saharan Africa: a systematic review

Embase search – 13/01/2021:

((HIV or HIV-1 or HIV AIDS or HIV infections) and (adult or older adult or older people or older individual or elderly) and (Africa or sub-Saharan Africa or Africa South of the Sahara) and (cognitive impairment or neurocognitive impairment or neurological complication or HIV-associated neurocognitive disorder or common mental disorder or depression or depressive symptoms or depressive disorder or anxiety or anxiety disorder or quality of life or health-related quality of life or grip strength or hand strength or frailty)).af.

Number of hits = 1284

Scopus search – 13/01/2021:

TITLE-ABS-KEY ((((((((hiv) OR (hiv-1)) OR (hiv/aids)) OR (hiv AND infections)) AND (((((adult) OR (older AND adult)) OR (older AND people)) OR (older AND individual)) OR (elderly))) AND (((africa) OR (sub-saharan AND africa)) OR (africa AND south AND of AND the AND sahara))) AND ((((((((((((((cognitive AND impairment) OR (neurocognitive AND impairment)) OR (neurological AND complication)) OR (hiv-associated AND neurocognitive AND disorder)) OR (common AND mental AND disorder)) OR (depression)) OR (depressive AND symptoms)) OR (depressive AND disorder)) OR (anxiety)) OR (anxiety AND disorder)) OR (quality AND of AND life)) OR (health-related AND quality AND of AND life)) OR (grip AND strength)) OR (hand AND strength)) OR (frailty)))

Number of hits = 1295

CINAHL search – 13/01/2021:

((HIV OR HIV-1 OR HIV AIDS OR HIV infections)) AND ((adult OR older adult OR older people OR older individual OR elderly)) AND ((Africa OR sub-Saharan Africa OR Africa South of the Sahara)) AND ((cognitive impairment OR neurocognitive impairment OR neurological complication OR HIV-associated neurocognitive disorder OR common mental disorder OR depression OR depressive symptoms OR depressive disorder OR anxiety OR anxiety disorder OR quality of life OR health-related quality of life OR grip strength OR hand strength OR frailty))

Number of hits = 381

Total number of hits across the databases = 8,741 articles

For peer review only - http://bmjopen.bmj.com/site/about/guidelines.xhtml

Supplementary Files – Mental health and Wellbeing of older adults living with HIV in sub-Saharan Africa: a systematic review

Supplementary Table 1: Summary of prevalence estimates and correlates for common mental disorders

Author, publication year & country	Study design	Sample size	Treatment status of PLWH	Cut-off score of the tool used	Information on local tool validation	Prevalence estimates/other results for PLWH50+	Correlates reported
Studies that used the 9-item Patient Health Questionnaire (PHQ-9) to assess depression							
Abadiga, M. (2019); Ethiopia	Cross-sectional	55 PLWH48+; 338 PLWH (18-48 years)	All on ART	≥5	Previously validated in Ethiopia	49.1%	Reported correlates not aggregated by age.
Eshetu, D. A., et al. (2015) ; Ethiopia	Cross-sectional	60 PLWH50+; 356 PLWH (20-49 years)	56.2% on ART	≥5	Sensitivity of 88% and Specificity of 88%	46.7%	Reported correlates not aggregated by age
Torgersen, J., et al. (2019); Botswana	Cross-sectional	125 PLWH50+; 789 PLWH (21-49 years)	ART naïve	≥10	NR	16.0%	NR
Cholera, R., et al. (2017); South Africa	Cross-sectional	28 PLWH50+; 312 PLWH (18-49 years)	ART naïve	≥10	Previously validated against the MINI yielding 78.7% sensitivity and 83.4% specificity in the study population	46.4%	NR
Asangbeh, S. L., et al. (2016) ; Cameroon	Cross-sectional	62 PLWH50+; 140 PLWH (21-49 years)	All on ART	≥10	Validated in a previous study in Cameroon	37.0%	Reported correlates not aggregated by age.
Duko, B., et al. (2018); Ethiopia	Cross-sectional	31 PLWH50+; 352 PLWH (18-54 years)	93.2% on ART	≥11	Previously validated yielding 86% sensitivity and 67% specificity	45.2%	Reported correlates not aggregated by age.
Studies that used the Center for Epidemiological Studies Depression scale (CES-D) to assess depression							
Bernard, C., et al. (2020); Coˆte d'Ivoire & Senegal	Cross-sectional	334 PLWH50+	All on ART	A total score ≥ 17 for men and ≥ 23 for women	Relied on previous validation of the tool from other African countries (Uganda, South Africa and Zambia)	17.9% (95% CI: 13.8-22).	PLWH with severe depressive symptoms were more likely to be **unemployed** (AOR = 2.8; 95% CI: 1.4–5.7); **current or former tobacco smokers** (AOR = 2.6; 95% CI: 1.3–5.4) but **were less likely to be overweight or obese** (AOR = 0.4; 95% CI: 0.2–0.8).
Rohr, J. K., et al. (2020); South Africa	Population survey	1048 PLWH40+; 3512 HIV uninfected older adults ≥40 years	68.6% on ART	≥4 symptoms	NR	8.0% among PLWH40+ versus 10.0% among HIV uninfected older adults ≥40 years.	NR
Moucheraud, C., et al. (2020); Malawi	Cross-sectional	74 PLWH50+ and 60 young PLWH [30-49] years.	All on ART	Mild/major depression symptoms = ≥16 CESD scores; Major depression symptoms = ≥27	NR	Symptoms of mild/major depression: 32.4% among PLWH50+; Major depression symptoms: 10.8% among PLWH50+	Older adults reported less few depressive symptoms (mild depression: AOR 0.23 p = 0.002; major depression: AOR 0.16, p = 0.004)

For peer review only - http://bmjopen.bmj.com/site/about/guidelines.xhtml

Supplementary Files – Mental health and Wellbeing of older adults living with HIV in sub-Saharan Africa: a systematic review

Author, publication year & country	Study design	Sample size	Treatment status of PLWH	Cut-off score of the tool used	Information on local tool validation	Prevalence estimates/other results for PLWH50+	Correlates reported
Geldsetzer, P., et al. (2019); South Africa	Population-based survey	1,037 PLWH40+; 4,022 HIV uninfected older adults ≥40 years	63.9% on ART	NR	Not validated previously. The current version underwent intensive cultural adaptation	None of the chronic conditions was significantly associated with depressive symptoms in multivariable regressions.	Older age was positively associated with depression, whereas being married and employment was negatively associated.
Olley, B. O., et al. (2017) ; Nigeria	Cross-sectional	44 PLWH50+; 458 PLWH (18-50 years)	All on ART	≥15	Cronbach's alpha = 0.79. Item-total correlation coefficient ranged between 0.65 and 0.87	20.5%	Not aggregated by age
Kaharuza, F. M., et al. (2006) ; Uganda	Cross-sectional	94 PLWH50+; 923 PLWH (18-50 years)	ART naïve	≥23	Internally consistent (alpha = 0.9)	58.5%	Older age (>50 years) was positively associated with depressive symptoms; AOR 1.93 (95% CI: 1.09, 3.42).
Studies that used the Mini International Neuropsychiatric Interview (MINI) to assess depression							
Asrat, B., et al. (2020); Ethiopia	Cross-sectional	54 PLWH50+; 337 PLWH (18-49 years)	All on ART	NR	Previously adapted in Ethiopia	29.6%	Reported correlates not aggregated by age.
Musinguzi, K., et al. (2018); Uganda	Cross-sectional	21 PLWH50+; 180 PLWH (18-49 years)	ART naïve	NR	NR	14.3%	Reported correlates not aggregated by age.
Kinyanda, E., et al. (2016); Uganda	Cross-sectional	244 PLWH50+; 224 HIV affected but uninfected older adults ≥50 years	9.8% on ART	NR	NR	11.9% among PLWH50+; 6.3% in HIV affected but uninfected older adults ≥50 years	MDD was significantly associated with declining SES, increasing disability scores, decreasing mean grip strength, reported back pain, and not having hypertension.
Mugisha, J. O., et al. (2016) ; Uganda	Cross-sectional	244 PLWH50+; 227 HIV uninfected older adults ≥50 years	90.6% on ART	NR	Locally adapted	9.5% among PLWH50+; 5.8% in the uninfected older adults ≥50 years	NR
Akena, D., et al. (2012); Uganda	Cross-sectional	51 PLWH50+; 317 PLWH (18-49 years)	NR	≥5 of the 9 DSM-IV-TR symptoms for major depression	Not locally validated	11.8%	Reported correlates not aggregated by age.

For peer review only - http://bmjopen.bmj.com/site/about/guidelines.xhtml

Supplementary Files – Mental health and Wellbeing of older adults living with HIV in sub-Saharan Africa: a systematic review

Author, publication year & country	Study design	Sample size	Treatment status of PLWH	Cut-off score of the tool used	Information on local tool validation	Prevalence estimates/other results for PLWH50+	Correlates reported
Studies that used the geriatric depression scale to assess depression							
Kalomo, E. N., et al. (2020); Namibia	Cross-sectional	147 PLWH50+	All on ART	≥3 symptoms	Cronbach alpha of 0.73 in the current study	46.1%	There was a positive association between HIV stigma (negative self-perceptions) and depression and a negative relationship between resilience and depression. Also, being female and higher education were protective factors and disclosure of HIV status was a risk factor
Eaton, P., et al. (2020); Tanzania	Cross-sectional	253 PLWH50+	94.8% on ART	≥5 symptoms	NR	17.4%	Not reported
Studies that used the Composite International Diagnostic Interview (CIDI) to assess depression							
Nyirenda, M., et al. (2013); South Africa	Cross-sectional	203 PLWH50+; 219 HIV uninfected older adults ≥50 years	23.7% on ART	ICD 10-DCR criteria	Locally adapted	14.8% among PLWH50+; 30.1 % in the uninfected older adults ≥50 years	Being female (aOR 3.04, 95% CI 1.73–5.36), receiving a government grant (aOR 0.34, 95% CI 0.15–0.75), urban residency (aOR 1.86, 95% CI 1.16–2.96) and adult caregiving (aOR 2.37, 95% CI 1.37–4.12) were significantly associated with any depressive episode
Negin, J., et al. (2012); South Africa	Cross-sectional	142 PLWH50+; 2,722 HIV uninfected older adults ≥50 years	NR	NR	Locally adapted	6.0% among PLWH50+; 4.8% in the uninfected older adults ≥50 years	NR
Studies that used other methods to assess depression							
Kellett-Wright, J., et al. (2020); Tanzania	Cross-sectional	235 PLWH50+	95.5% on ART	DSM-IV; cut-off NR	NR	16.6%	NR
Motumma, A., et al. (2019); Ethiopia	Cross-sectional	122 PLWH40+; 298 HIV infected young adults (18-40) years	All on ART	A score of ≥7 of Self-Reporting Questionnaire (SRQ-20)	Previously validated in Ethiopia, with high sensitivity (85.7%) and specificity (75.6%).	23.0%	Correlates not aggregated by age
Manne-Goehler, J., et al. (2019); Uganda	Cross-sectional	154 PLWH40+; 142 HIV uninfected older adults ≥40 years	All on ART	Mean score of ≥1.75 on the 15-Item Hopkins Checklist (HSCL)	Previously validated in the same population	21.4% among PLWH40+; 33.8% in uninfected older adults ≥40 years	PLWH on ART and those falling in the highest wealth quartile had a lower prevalence of probable depression. In comparison, women had a significantly higher prevalence of depression than men. Education and age were not significantly associated with depression.

For peer review only - http://bmjopen.bmj.com/site/about/guidelines.xhtml

Supplementary Files – Mental health and Wellbeing of older adults living with HIV in sub-Saharan Africa: a systematic review

Author, publication year & country	Study design	Sample size	Treatment status of PLWH	Cut-off score of the tool used	Information on local tool validation	Prevalence estimates/other results for PLWH50+	Correlates reported
Tesfaw, G., et al. (2016); Ethiopia	Cross-sectional	59 PLWH50+; 358 PLWH (19-49 years)	All on ART	A cut-off ≥8 on the 7-item Hospital Anxiety and Depression Scale	Previously validated	39.0%	Not aggregated by age
Shumba, C., et al. (2013); Uganda	Cross-sectional	91 PLWH50+; 584 PLWH (19-49 years)	All on ART	A cut-off of ≥1 on a locally adapted 5-item symptom questionnaire	Cronbach's alpha of 0.87	53.9%	NR
Berhe, H. and A. Bayray (2013); Ethiopia	Cross-sectional	19 PLWH54+; 250 PLWH (18-54 years)	NR	A score of ≥8 on the 21-Item Hamilton Depression Scale	NR	57.9%	Reported correlates not aggregated by age.
Studies reporting anxiety							
Obimakinde, A. M., et al. (2020) ; Nigeria	Cross-sectional	62 PLWH60+; 162 HIV uninfected older adults ≥60 years	All on ART	Extracted from participants clinical notes	NR	3.2% among PLWH50+; 3.2% in HIV uninfected older adults	NR
Olagunju, A. T., et al. (2012) ; Nigeria	Cross-sectional	24 PLWH50+; 276 PLWH (18-50 years)	All on ART	Clinical Assessment in Neuropsychiatry (SCAN);	NR	20.8%	Not aggregated by age

Notes: ART – *Antiretroviral treatment*; **AOR** – *Adjusted odds ratio*; **CES-D** – *Center for Epidemiologic Studies Depression Scale*; **CI** – *Confidence interval*; **CIDI** – *Composite International Diagnostic Interview*; **DSM-IV**– *Diagnostic and Statistical Manual of Mental Disorders, fourth edition;* **HSCL** – *Hopkins Symptoms Checklist*; **MDD** – *Major depressive disorder*; **MINI** – *Mini International Neuropsychiatric Interview*; **NR** – *Not reported*; **PLWH** – *People living with HIV*; **PLWH50+** – *People living with HIV ≥50 years old*; **SCAN** – *Clinical Assessment in Neuropsychiatry*

For peer review only - http://bmjopen.bmj.com/site/about/guidelines.xhtml

Supplementary Files – Mental health and Wellbeing of older adults living with HIV in sub-Saharan Africa: a systematic review

Supplementary Table 2. Modified Newcastle-Ottawa Risk of Bias Assessment

Author	Representativeness of the sample	Sample size	Comparability between respondents and non-respondents	Outcome ascertainment	Quality of Statistics reporting	Total quality score
**Abadiga, M. (2019).**	**1**	**0**	**1**	**1**	**1**	**4**
Akena, D., et al. (2012).	1	0	0	0	1	2
Asangbeh, S. L., et al. (2016)	0	0	0	1	1	2
Asiimwe, S. B., et al. (2020).	1	1	1	1	1	5
Asrat, B., et al. (2020).	1	0	1	1	1	4
Atashili, J., et al. (2013)	0	0	1	0	1	2
Berhe, H. and A. Bayray (2013)	0	0	0	0	1	1
Bernard, C., et al. (2020).	1	1	0	0	1	3
Bernard, C., et al. (2020).	1	1	0	0	1	3
Bristow, C., et al. (2021).	1	1	1	1	1	5
Cassimjee, N. and P. K. Motswai (2017)	0	0	0	1	1	2
Cholera, R., et al. (2017).	1	0	1	1	1	4
Duko, B., et al. (2018).	1	0	0	1	1	3
Eaton, P., et al. (2020)	1	1	0	1	1	4
Edwards, A., et al. (2020).	1	1	1	0	1	4
Eshetu, D. A., et al. (2015).	0	0	0	0	1	1
Filteau, S., et al. (2017).	1	1	0	1	1	4
Geldsetzer, P., et al. (2019).	1	1	1	0	1	4
Harding, R., et al. (2014).	0	0	0	1	1	2
Joska, J. A., et al. (2019).	1	1	0	0	1	3
Kaharuza, F. M., et al. (2006)	1	0	0	1	1	3
Kalomo, E. N., et al. (2020)	0	1	0	0	1	2
Kellett-Wright, J., et al. (2020)	1	1	0	1	1	4
Kinyanda, E., et al. (2016)	1	1	0	1	1	4
Kobayashi, L. C., et al. (2019)	1	1	1	1	1	5
Maniragaba, F., et al. (2018)	1	0	0	0	1	2
Manne-Goehler, J., et al. (2019).	0	1	0	1	1	3
Motumma, A., et al. (2019).	1	1	1	1	1	5
Moucheraud, C., et al. (2020)	0	0	0	1	1	2
Mugendi, A., et al. (2019).	0	0	0	0	1	1
Mugisha, J., et al. (2013)	1	1	0	1	1	4
Mugisha, J. O., et al. (2016).	1	1	0	1	1	4
Musinguzi, K., et al. (2018).	1	0	0	0	1	2
Negin, J., et al. (2012)	1	1	0	1	1	4
Nyirenda, M., et al. (2012).	1	1	0	1	1	4
Nyirenda, M., et al. (2013).	1	1	0	1	1	4
Nyirenda, M., et al. (2013)	1	1	0	1	1	4
Obimakinde, A. M., et al. (2020).	0	0	1	0	1	2
Olagunju, A. T., et al. (2012).	1	0	0	1	1	3
Olley, B. O., et al. (2017).	0	0	0	1	1	2
Oumar, G., et al. (2020).	0	1	0	0	1	2
Parcesepe, A. M., et al. (2020).	0	0	0	1	1	2
Rohr, J. K., et al. (2020)	1	1	1	0	1	4
Scholten, F., et al. (2011).	1	1	0	1	1	4
Shumba, C., et al. (2013).	1	0	0	1	1	3
Ssonko, M., et al. (2018).	1	1	0	0	1	3
Tesfaw, G., et al. (2016)	1	0	1	1	1	4
Torgersen, J., et al. (2019).	0	1	1	0	1	3
Tsegaw, M., et al. (2017)	0	0	1	0	1	2
Yaya, I., et al. (2019).	1	1	1	0	1	4

For peer review only

For peer review only - http://bmjopen.bmj.com/site/about/guidelines.xhtml

Research Checklist– Mental health and Wellbeing of older adults living with HIV in sub-Saharan Africa: a systematic review

PRISMA 2020 Checklist

Section and Topic	Item #	Checklist item	Location where item is reported
**TITLE**			
Title	1	Identify the report as a systematic review.	Page 1
**ABSTRACT**			
Abstract	2	See the PRISMA 2020 for Abstracts checklist.	Page 2
**INTRODUCTION**			
Rationale	3	Describe the rationale for the review in the context of existing knowledge.	Page 3
Objectives	4	Provide an explicit statement of the objective(s) or question(s) the review addresses.	Page 4
**METHODS**			
Eligibility criteria	5	Specify the inclusion and exclusion criteria for the review and how studies were grouped for the syntheses.	Page 4
Information sources	6	Specify all databases, registers, websites, organisations, reference lists and other sources searched or consulted to identify studies. Specify the date when each source was last searched or consulted.	Page 4
Search strategy	7	Present the full search strategies for all databases, registers and websites, including any filters and limits used.	Page 4 and supplementary table 1
Selection process	8	Specify the methods used to decide whether a study met the inclusion criteria of the review, including how many reviewers screened each record and each report retrieved, whether they worked independently, and if applicable, details of automation tools used in the process.	Page 4
Data collection process	9	Specify the methods used to collect data from reports, including how many reviewers collected data from each report, whether they worked independently, any processes for obtaining or confirming data from study investigators, and if applicable, details of automation tools used in the process.	Page 4
Data items	10a	List and define all outcomes for which data were sought. Specify whether all results that were compatible with each outcome domain in each study were sought (e.g. for all measures, time points, analyses), and if not, the methods used to decide which results to collect.	Page 4 and 5
	10b	List and define all other variables for which data were sought (e.g. participant and intervention characteristics, funding sources). Describe any assumptions made about any missing or unclear information.	N/A
Study risk of bias assessment	11	Specify the methods used to assess risk of bias in the included studies, including details of the tool(s) used, how many reviewers assessed each study and whether they worked independently, and if applicable, details of automation tools used in the process.	Page 4 and 5
Effect measures	12	Specify for each outcome the effect measure(s) (e.g. risk ratio, mean difference) used in the synthesis or presentation of results.	Page 4
Synthesis methods	13a	Describe the processes used to decide which studies were eligible for each synthesis (e.g. tabulating the study intervention characteristics and comparing against the planned groups for each synthesis (item #5)).	N/A
	13b	Describe any methods required to prepare the data for presentation or synthesis, such as handling of missing summary	N/A

For peer review only

Research Checklist– Mental health and Wellbeing of older adults living with HIV in sub-Saharan Africa: a systematic review

Section and Topic	Item #	Checklist item	Location where item is reported
		statistics, or data conversions.	
	13c	Describe any methods used to tabulate or visually display results of individual studies and syntheses.	Page 5
	13d	Describe any methods used to synthesize results and provide a rationale for the choice(s). If meta-analysis was performed, describe the model(s), method(s) to identify the presence and extent of statistical heterogeneity, and software package(s) used.	Page 5
	13e	Describe any methods used to explore possible causes of heterogeneity among study results (e.g. subgroup analysis, meta-regression).	N/A
	13f	Describe any sensitivity analyses conducted to assess robustness of the synthesized results.	N/A
Reporting bias assessment	14	Describe any methods used to assess risk of bias due to missing results in a synthesis (arising from reporting biases).	N/A
Certainty assessment	15	Describe any methods used to assess certainty (or confidence) in the body of evidence for an outcome.	N/A
**RESULTS**			
Study selection	16a	Describe the results of the search and selection process, from the number of records identified in the search to the number of studies included in the review, ideally using a flow diagram.	Page 5 and 6 and figure 1
	16b	Cite studies that might appear to meet the inclusion criteria, but which were excluded, and explain why they were excluded.	N/A
Study characteristics	17	Cite each included study and present its characteristics.	Page 20 to 24 and supplementary Table 1
Risk of bias in studies	18	Present assessments of risk of bias for each included study.	Supplementary table 2
Results of individual studies	19	For all outcomes, present, for each study: (a) summary statistics for each group (where appropriate) and (b) an effect estimate and its precision (e.g. confidence/credible interval), ideally using structured tables or plots.	Pages 20 to 24 and supplementary table 1
Results of syntheses	20a	For each synthesis, briefly summarise the characteristics and risk of bias among contributing studies.	Supplementary table 2
	20b	Present results of all statistical syntheses conducted. If meta-analysis was done, present for each the summary estimate and its precision (e.g. confidence/credible interval) and measures of statistical heterogeneity. If comparing groups, describe the direction of the effect.	N/A
	20c	Present results of all investigations of possible causes of heterogeneity among study results.	N/A
	20d	Present results of all sensitivity analyses conducted to assess the robustness of the synthesized results.	N/A
Reporting biases	21	Present assessments of risk of bias due to missing results (arising from reporting biases) for each synthesis assessed.	N/A

For peer review only

Research Checklist– Mental health and Wellbeing of older adults living with HIV in sub-Saharan Africa: a systematic review

Section and Topic	Item #	Checklist item	Location where item is reported
Certainty of evidence	22	Present assessments of certainty (or confidence) in the body of evidence for each outcome assessed.	N/A
**DISCUSSION**			
Discussion	23a	Provide a general interpretation of the results in the context of other evidence.	Pages 10-14
	23b	Discuss any limitations of the evidence included in the review.	Page 13-14
	23c	Discuss any limitations of the review processes used.	Page 13-14
	23d	Discuss implications of the results for practice, policy, and future research.	Page 14
**OTHER INFORMATION**			
Registration and protocol	24a	Provide registration information for the review, including register name and registration number, or state that the review was not registered.	Page 2 and 4
	24b	Indicate where the review protocol can be accessed, or state that a protocol was not prepared.	Page 4
	24c	Describe and explain any amendments to information provided at registration or in the protocol.	N/A
Support	25	Describe sources of financial or non-financial support for the review, and the role of the funders or sponsors in the review.	Page 14
Competing interests	26	Declare any competing interests of review authors.	Page 14
Availability of data, code and other materials	27	Report which of the following are publicly available and where they can be found: template data collection forms; data extracted from included studies; data used for all analyses; analytic code; any other materials used in the review.	Page 14

For peer review only
